# Development of in vitro enteroids derived from bovine small intestinal crypts

**DOI:** 10.1186/s13567-018-0547-5

**Published:** 2018-07-03

**Authors:** Carly A. Hamilton, Rachel Young, Siddharth Jayaraman, Anuj Sehgal, Edith Paxton, Sarah Thomson, Frank Katzer, Jayne Hope, Elisabeth Innes, Liam J. Morrison, Neil A. Mabbott

**Affiliations:** 1 The Roslin Institute & Royal (Dick) School of Veterinary Sciences, University of Edinburgh, Easter Bush, Midlothian, EH25 9RG UK; 2 College of Medical, Veterinary and Life Sciences, University of Glasgow, 5/20 Sir Graeme Davies Building, 120 University Place, Glasgow, G12 8TA UK; 3 Moredun Research Institute, Pentlands Science Park, Bush Loan, Midlothian, EH26 0PZ UK

## Abstract

Cattle are an economically important domestic animal species. In vitro 2D cultures of intestinal epithelial cells or epithelial cell lines have been widely used to study cell function and host–pathogen interactions in the bovine intestine. However, these cultures lack the cellular diversity encountered in the intestinal epithelium, and the physiological relevance of monocultures of transformed cell lines is uncertain. Little is also known of the factors that influence cell differentiation and homeostasis in the bovine intestinal epithelium, and few cell-specific markers that can distinguish the different intestinal epithelial cell lineages have been reported. Here we describe a simple and reliable procedure to establish in vitro 3D enteroid, or “mini gut”, cultures from bovine small intestinal (ileal) crypts. These enteroids contained a continuous central lumen lined with a single layer of polarized enterocytes, bound by tight junctions with abundant microvilli on their apical surfaces. Histological and transcriptional analyses suggested that the enteroids comprised a mixed population of intestinal epithelial cell lineages including intestinal stem cells, enterocytes, Paneth cells, goblet cells and enteroendocrine cells. We show that bovine enteroids can be successfully maintained long-term through multiple serial passages without observable changes to their growth characteristics, morphology or transcriptome. Furthermore, the bovine enteroids can be cryopreserved and viable cultures recovered from frozen stocks. Our data suggest that these 3D bovine enteroid cultures represent a novel, physiologically-relevant and tractable in vitro system in which epithelial cell differentiation and function, and host–pathogen interactions in the bovine small intestine can be studied.

**Electronic supplementary material:**

The online version of this article (10.1186/s13567-018-0547-5) contains supplementary material, which is available to authorized users.

## Introduction

The mucosal surface that lines the mammalian gastrointestinal tract is continuously exposed to commensal and pathogenic microorganisms. Throughout the intestine a single layer of epithelial cells sealed by tight-junctions acts to restrict access of these microorganisms, food macromolecules and solutes to the underlying tissues. The intestinal epithelium is self-renewing and replaced approximately every 5–7 days. The crypts of Lieberkühn in the small and large intestines contain leucine-rich repeat-containing G protein-coupled receptor 5 (LGR5)-expressing intestinal stem cells [1]. These actively dividing LGR5^+^ intestinal stem cells produce highly proliferative transit-amplifying daughter cells that can differentiate into all of the distinct epithelial cell lineages that are present within the lining of the small intestine, including: enterocytes, goblet cells, enteroendocrine cells, tuft cells, and Paneth cells [1]. The differentiated cells then migrate along the villus epithelium where they perform their physiological roles before being shed into the lumen via apoptosis as they reach the villus tip. In Peyer’s patches subsequent stimulation via the cytokine receptor activator of NF-κB ligand (RANKL) mediates the differentiation of RANK-expressing enterocytes into antigen-sampling M cells [2, 3]. The Paneth cells, in contrast, are long-lived and reside within the crypt base nestled amongst the LGR5^+^ intestinal stem cells. Paneth cells release antimicrobial products which protect the crypt from bacterial infection [4, 5], as well as providing important homeostatic factors including epidermal growth factor (EGF), Notch ligand Dll4, transforming growth factor—(TGF-)α and Wnt-signalling molecules which help maintain the LGR5^+^ intestinal stem cells [6]. Paneth cells are absent in the large intestine, where regenerating islet-derived family member 4 (REG4)-expressing deep secretory cells play a similar role in the maintenance of LGR5+ intestinal stem cells in colonic crypts [7].

In vitro cultures of 2D monolayers of intestinal epithelial cells or epithelial cell lines have been widely used to study cell function and host–pathogen interactions in the mammalian intestine. For example, immortalized [8, 9] or cloned [10] intestinal epithelial cell lines have been developed from the bovine intestine.

However, these cultures lack the cellular diversity encountered in the intestinal epithelium and the physiological relevance of monocultures of transformed cell lines is uncertain. Monolayers of the Caco-2 human enterocyte cell line co-cultivated with B cells have also been used as an in vitro system to study M cells, and appear to reproduce their antigen-sampling properties [11]. Whether differentiated Caco-2 cells accurately reflect the in vivo characteristics of M cells is unclear, as transcriptional analysis shows they lack expression of many important M-cell marker genes [12].

The ability of LGR5^+^ stem cells to generate all the epithelial cell lineages within the intestine [1] has been exploited through the development of exciting and physiologically-relevant, in vitro models of the intestinal epithelium [13]. Intestinal crypts or individual LGR5^+^ intestinal stem cells are cultivated in a laminin-rich Matrigel matrix in the presence of the essential homeostatic support factors EGF, Noggin and R-spondin 1 [13]. The crypts in these cultures initially seal over to form enterospheres which then undergo multiple crypt budding events similar to crypt fission in vivo [13, 14]. As further expansion occurs, 3D enteroids or “mini guts” are formed, comprising a single layer of intestinal epithelial cells surrounding a central closed lumen with many crypt bud domains [13]. Importantly, these enteroid cultures maintain much of the cellular diversity present within the intestinal epithelium in vivo [6, 13, 15–18]. Enteroid cultures have been used in many laboratories to study cell differentiation and function in the intestinal epithelium [3, 6, 13, 15–23]. The central closed lumen of enteroids also enables the uptake of particulate antigens [19] and host–pathogen interactions at the mucosal surface [24–26] to be studied.

Cattle are an economically important domestic animal species in many countries worldwide. Little is known of the factors which influence cell differentiation and maintenance in the bovine intestinal epithelium. Furthermore, few cell-specific markers which reliably distinguish individual bovine intestinal epithelial cell populations have been described. Cattle are also natural reservoirs for a range of important enteric pathogens, including *Salmonella*, *Escherichia coli* O157:H7, *Mycobacterium avium* subspecies *paratuberculosis* and *Cryptosporidium parvum*. The effective control of these and other pathogens in cattle has significant economic implications, and is important for food security as many also have zoonotic potential. Few physiologically-relevant in vitro systems are available to accurately study the interactions between enteric pathogens and the bovine intestinal epithelium, and most data have been derived from the analysis of 2D monolayers of epithelial cell lines or primary epithelial cells [27, 28]. In the current study we describe the development of a 3D in vitro culture system that is more representative of the cellular diversity within the bovine intestinal epithelium. Enteroid cultures were prepared from bovine small intestinal (ileal) crypts, and histological and transcriptional analyses suggested that they comprised a mixed population of intestinal epithelial cell lineages. These bovine enteroids could be maintained for long periods in culture through multiple serial passages, and be cryopreserved for later use. Our data suggest that these bovine small intestinal crypt-derived enteroid cultures represent a useful physiologically-relevant in vitro system to study epithelial cell differentiation and function, and host–pathogen interactions in the bovine small intestine.

## Materials and methods

### Animals

All tissues used in this study were obtained from healthy male British Holstein–Friesian (*Bos taurus*) calves (< 1 month old).

### Ethics statement

All studies and regulatory licences were approved by both The Roslin Institute’s and University of Edinburgh’s ethics committees.

### Isolation of intestinal crypts

The ileum was removed from calves at post-mortem and approximately 10 cm portions collected into sterile ice-cold phosphate-buffered saline (PBS) containing 25 μg/mL gentamicin and 100 U/mL penicillin/streptomycin (Sigma-Aldrich, Poole, UK). The ileum was opened longitudinally and the mucus layer gently scraped off using a glass slide and discarded. The remaining sub-mucosal layer was removed by scraping, collected into a 50 mL falcon tube and suspended in Hank’s Balanced Salt Solution (HBSS; Gibco, ThermoFisher Scientific, UK) containing 25 µg/mL gentamicin and 100 U/mL penicillin/streptomycin. The sealed tube was then shaken vigorously and washed at 400 × *g* for 2 min until the supernatant was clear. The supernatant was then removed and the pellet re-suspended in 25 mL of Dulbecco’s Modified Eagle’s Medium (DMEM; Gibco) containing 1.0% foetal calf serum (TCS Biosciences, UK), 25 μg/mL gentamicin, 100 U/mL penicillin/streptomycin, 75 U/mL collagenase type1-A (C2674; Sigma-Aldrich) and 20 µg/mL dispase I (Roche, Germany). Crypts were then liberated from the tissues by incubation at 37 °C with constant shaking at 200 rpm for 40 min. Following digestion, the integrity of bovine intestinal crypts was visually assessed using a Zeiss Axiovert-25 microscope. The supernatant containing the intestinal crypts was then washed by centrifugation at 400 × *g* for 2 min. The crypts were then finally re-suspended in advanced DMEM/F12 medium (Gibco) containing 1X B27 supplement minus vitamin A (ThermoFisher Scientific), 25 μg/mL gentamicin and 100 U/mL penicillin/streptomycin.

### Enteroid cultivation

To prepare the enteroids 200 bovine intestinal crypts in 100 µL advanced DMEM/F12 medium were added to 150 μL of BD Growth Factor Reduced Matrigel Matrix (BD Biosciences, UK) and 50 μL droplets added to wells of a pre-warmed 24-well plate (Nunc, ThermoFisher Scientific). The Matrigel was then allowed to polymerise by incubation for 10 min at 37 °C in a 5% CO_2_/air atmosphere for 10 min. The crypts/enteroids were then maintained in 700 μL of IntestiCult Organoid Growth Medium (Mouse) (STEMCELL Technologies, UK) containing 50 μg/mL gentamicin at 37 °C in a 5% CO_2_/air atmosphere, and the fresh medium replaced every 2–3 days. Where indicated, the enteroid cultures were supplemented with 100 mM Y-27632 (Cambridge Bioscience, UK), 5 mM LY2157299 (Cambridge Bioscience) and 100 mM SB202190 (Enzo Life Sciences, UK).

### Enteroid passage

To passage the enteroids the growth medium was removed and 1 mL ice-cold advanced DMEM/F12 medium containing 1X B27 supplement minus vitamin A, 25 μg/mL gentamicin and 100 U/mL penicillin/streptomycin was added directly to the Matrigel plug. The resulting suspension containing the enteroids was then collected into a Pyrex FACS tube (Corning, Wycombe, UK) and allowed to settle. The supernatant was discarded and the enteroids re-suspended in approximately 500 μL of medium and transferred to a 1.5 mL Eppendorf tube. The enteroids were then mechanically disrupted by vigorous pipetting using a 200 µL pipette tip bent at a 90° angle. The dissociated crypts were allowed to settle by gravity, the supernatant was removed and the crypts re-suspended in fresh DMEM/F12 medium containing 1X B27 supplement minus vitamin A, 25 μg/mL gentamicin and 100 U/mL penicillin/streptomycin. The number of crypts was counted and enteroid cultures established as described above.

### Enteroid cryopreservation

To cryopreserve the enteroids the growth medium was removed and 1 mL ice-cold advanced DMEM/F12 medium containing 1X B27 supplement minus vitamin A, 25 μg/mL gentamicin and 100 U/mL penicillin/streptomycin was added directly to the Matrigel plug. The resulting enteroid suspension was then pelleted by centrifugation at 290 × *g* for 5 min at 4 °C. The enteroids were then resuspended in Cryostor CS10 cryopreservation medium (STEMCELL Technologies) at 1000 enteroids/mL. Cryovials were stored overnight in a Mr Frosty freezing container (ThermoFisher Scientific) at −80 °C, and then transferred to −155 °C for long-term storage.

To resuscitate the enteroids, the cryovials were thawed in a water bath at 37 °C. The enteroid suspension was then transferred to a 15 mL falcon tube containing 2 mL advanced DMEM/F12 medium containing 1X B27 supplement minus vitamin A, 25 μg/mL gentamicin and 100 U/mL penicillin/streptomycin and 1% BSA. The cryovial and lid were also washed twice with 1 mL of the above medium, and added to the enteroid suspension. The enteroids were then pelleted by centrifugation at 290 × *g* for 5 min at 4 °C, and cultivated as above.

### mRNA extraction

To extract mRNA from the enteroid cultures the growth medium was first removed and 1.0 mL ice-cold advanced DMEM/F12 medium containing 1X B27 supplement minus vitamin A, 25 μg/mL gentamicin and 100 U/mL penicillin/streptomycin was added directly to the Matrigel plug. The resulting suspension containing the enteroids was then collected into a Pyrex FACS tube, allowed to settle by gravity and washed twice with 1.0 mL ice-cold PBS. The supernatant was discarded and the enteroids re-suspended in 1.0 mL Corning Cell Recovery Solution (Corning) and incubated for 1 h at 4 °C with constant shaking at 120 rpm. The suspension was then centrifuged at 1200 × *g* for 5 min, and the pellet washed twice in 1.0 mL ice-cold PBS. The pellet was then lysed in 600 µL of the RLT buffer (Qiagen, Germany) containing 10 µg/mL 2-mercaptoethanol (Sigma-Aldrich, UK), and homogenized using a Qiashredder homogenizer (Qiagen). Total RNA was then extracted using a RNeasy mini kit (Qiagen) according to the manufacturer’s instructions which included a DNase digestion step to remove genomic DNA. The RNA was eluted into RNase-free water and the quality and concentration assessed using a TapeStation instrument (Agilent, Stockport, UK). RNA was stored at −80 °C before subsequent use.

### mRNA-seq analysis

From each sample 3 µg of total RNA was used. Seven RNA-seq libraries were prepared by Edinburgh Genomics (Edinburgh Genomics, Edinburgh, UK) using the TruSeq stranded mRNA-seq library preparation kit (Illumina, San Diego, USA) with one round of RiboZero Gold treatment. The individual libraries were then pooled, and the pool sequenced on three lanes of an Illumina HiSeq 4000 sequencing platform (Illumina). The libraries were sequenced with 150 base paired end reads at a depth of 147–191 M paired end reads/sample. Sequenced reads were checked for read quality using FastQC [29]. Pseudoaligner Kallisto (v 0.43.1; [30]) was used to measure transcript abundance level by aligning the reads against transcripts from the *Bos taurus* UMD3.1.1 assembly [31]. The transcript abundance value in transcripts per million (TPM) was aggregated for each sample and log_2_ transformed for downstream analysis.

### Data availability

The mRNA-seq analysis data sets are available via the following accession code in the Gene Expression Omnibus data base (GEO): GSE112674.

### Network analysis

The individual, annotated, crypts and enteroids mRNA-seq TPM data sets were combined, saved as an “.expression” file and imported into the tool Miru (Kajeka, Edinburgh, UK) [32–34]. This file format contains a unique identifier for each probe set on the array (gene symbol: target ID), followed by columns of gene annotation information and finally the non-log transformed data values (TPM) for each sample (each column of data being derived from a different sample). A sample-to-sample correlation matrix was first calculated from these non-log transformed gene expression data. A pairwise Pearson correlation matrix was calculated which comprised an all vs. all comparison of the expression profile of each probe set on the array. A graph was then plotted using all sample-to-sample relationships ≥ 0.96. In this graph all the nodes represent individual data sets (cells) and the edges that link these data sets represent Pearson correlation coefficients of *r* ≥ 0.96.

A pairwise transcript (gene)-to-transcript Pearson correlation matrix was then calculated based on each probe set’s profile across each of the samples. A Pearson correlation coefficient cut-off threshold of *r* ≥ 0.99 was selected and an undirected network graph of these data was generated. In this graph the nodes represent individual probe sets (genes/transcripts) and the edges between them Pearson correlation coefficients ≥ 0.99. The network was then clustered into groups of probe sets (genes) sharing similar profiles using the built-in Markov clustering algorithm using an inflation value (which controls the granularity of clustering) set to 2.2.

Genes in clusters of interest were then assessed for cellular functions and activities using a combination of literature review and bioinformatics. Significantly over-represented gene ontologies (GO) within clusters of interest were identified using the Molecular Signatures Database [35, 36] and GOstat [37]. For each GO term, the probability was calculated that the observed counts occurred by the random distribution of this GO term between the cluster of interest and the reference group (all genes). The Benjamini and Hochberg correction was used to control the false discovery rate of errors expected from multiple testing. Over-represented GO terms with *P* values < 0.05 calculated using the hypergeometric test, and false discovery rate < 0.05, were accepted as significant. Groups of genes often shared several GO terms that were indicative of the same biological process, molecular function or cellular compartment. In these instances the most informative GO terms within the top 20 identified are presented.

### Histology and immunohistochemistry (IHC)

To harvest the enteroids the growth medium was first removed and 1.0 mL ice-cold advanced DMEM/F12 medium containing 1x B27 supplement minus vitamin A, 25 μg/mL gentamicin and 100 U/mL penicillin/streptomycin was added directly to the Matrigel plug. The enteroids were then carefully collected into a Pyrex FACS tube using a glass pipette bent at a 90° angle. For whole mount staining, enteroids were fixed in 4% paraformaldehyde (PFA) for 45 min at 4 °C, permeabilised for 20 min in PBS containing 0.5% BSA/0.1% saponin, and then immunostained overnight at 4 °C with rabbit polyclonal anti-Ki67 (Abcam, Cambridge, UK). The enteroids were subsequently immunostained with Alexa Fluor 488-conjugated goat anti-rabbit IgG Ab (Invitrogen). To prepare cryosections, the enteroids were washed three times in ice-cold PBS, fixed in 4% PFA for 45 min at 4 °C, washed three more times in ice-cold PBS and suspended in 30% sucrose for 2 h. The enteroids were then embedded in optimal cutting temperature medium (VWR, Leighton Buzzard, UK) and stored at −80 °C until use. Frozen Sects. (8 µm in thickness) were cut using a cryostat and fixed in ice-cold methanol for 10 min, permeabilised with PBS/0.3% Triton X for 30 min and washed twice with PBS. To detect villin the sections were then immunostained with mouse anti-villin monoclonal antibody (clone 1D2C3, Santa Cruz Biotechnology Inc., Heidelberg, Germany). Where indicated the enteroids were also stained with Texas Red-conjugated phalloidin (ThermoFisher Scientific) to detect F-actin, or DAPI or Hoescht 33342 (ThermoFisher Scientific) to detect cell nuclei. Enteroids were then mounted with fluorescent mounting medium (Thermo Fisher Scientific) and examined using a Zeiss LSM710 confocal microscope (Zeiss, Welwyn Garden City, UK).

### Transmission electron microscopy (TEM)

Enteroids were fixed in 3.0% glutaraldehyde in 0.1 M sodium cacodylate buffer, pH 7.3, for 2 h then washed in three 10 min changes of 0.1 M sodium cacodylate buffer. The enteroids were then post-fixed in 1% osmium tetroxide in 0.1 M sodium cacodylate buffer for 45 min, and then washed in three 10 min changes of 0.1 M sodium cacodylate buffer. The enteroids were then dehydrated in 50, 70, 90 and 100% ethanol (×3) for 15 min each, then in two 10-min changes of propylene oxide and embedded in 812 resin. Sections 1 µm in thickness were then cut on an ultramicrotome, stained with toluidine blue, and viewed under a light microscope to select suitable areas for investigation. Ultrathin sections, 60 nm in thickness were then cut from the selected areas, stained in uranyl acetate and lead citrate and viewed using a JEOL JEM-1400 Plus TEM.

## Results

### In vitro cultivation and passage of bovine enteroids

**Figure 1:**
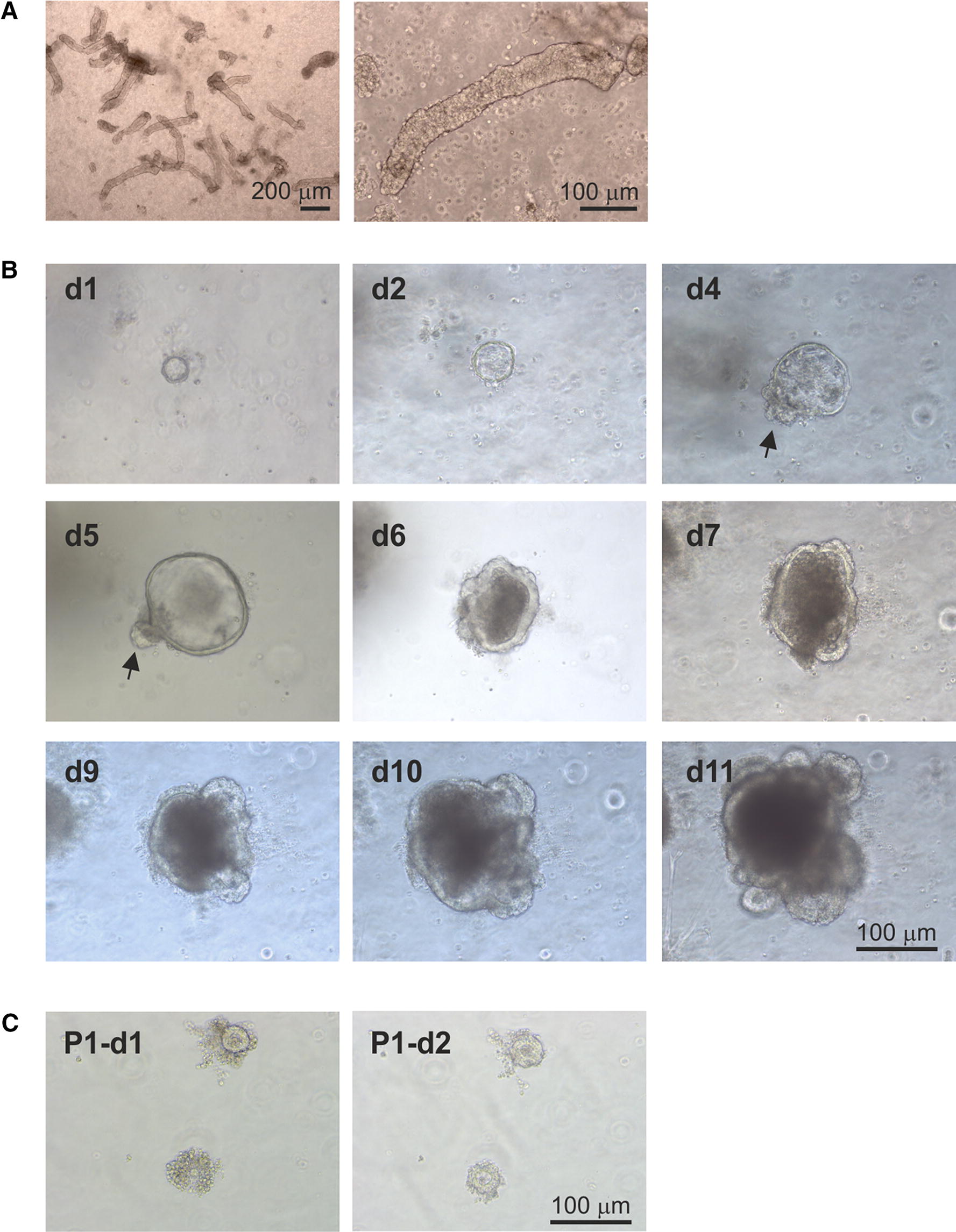
**Bovine enteroids cultivated in Intesticult medium alone. A** Morphology of freshly isolated bovine small intestinal crypts. **B** Representative images showing the growth and development of an individual enteroid cultivated in Intesticult medium alone during 11 days of culture. Arrows, crypt buds. **C** Enteroids cultivated in Intesticult medium alone did not survive after subsequent passage (P1: passage 1; d1: day 1 of culture).

Small intestinal crypts were isolated from the ileums of healthy < 1 month old calves (Figure 1A), embedded in Matrigel and cultivated in Intesticult medium using similar conditions to those which have been established for the maintenance of murine and human enteroids [38]. Within 24 h of culture the upper opening of the crypts had sealed over (Figure 1B). These enterosphere-like structures persisted until approximately day 4 of culture when occasional budding events were apparent (Figure 1B, arrows). This suggested division and expansion of the crypt domains, and the formation of enteroid-like structures with a central lumen. The size of the enteroids gradually increased with the increasing duration of the culture period (Figure 1B).

Many studies have shown that enteroid cultures prepared from mice and humans crypts can be maintained for many weeks/months through serial rounds of passage. Here, after 11 days of cultivation the enteroids were mechanically dissociated and the resulting crypt-like domains re-introduced into culture as above. Although the crypts sealed and formed enterospheres within 24 h, they did not survive extended culture (Figure 1C).

**Figure 2:**
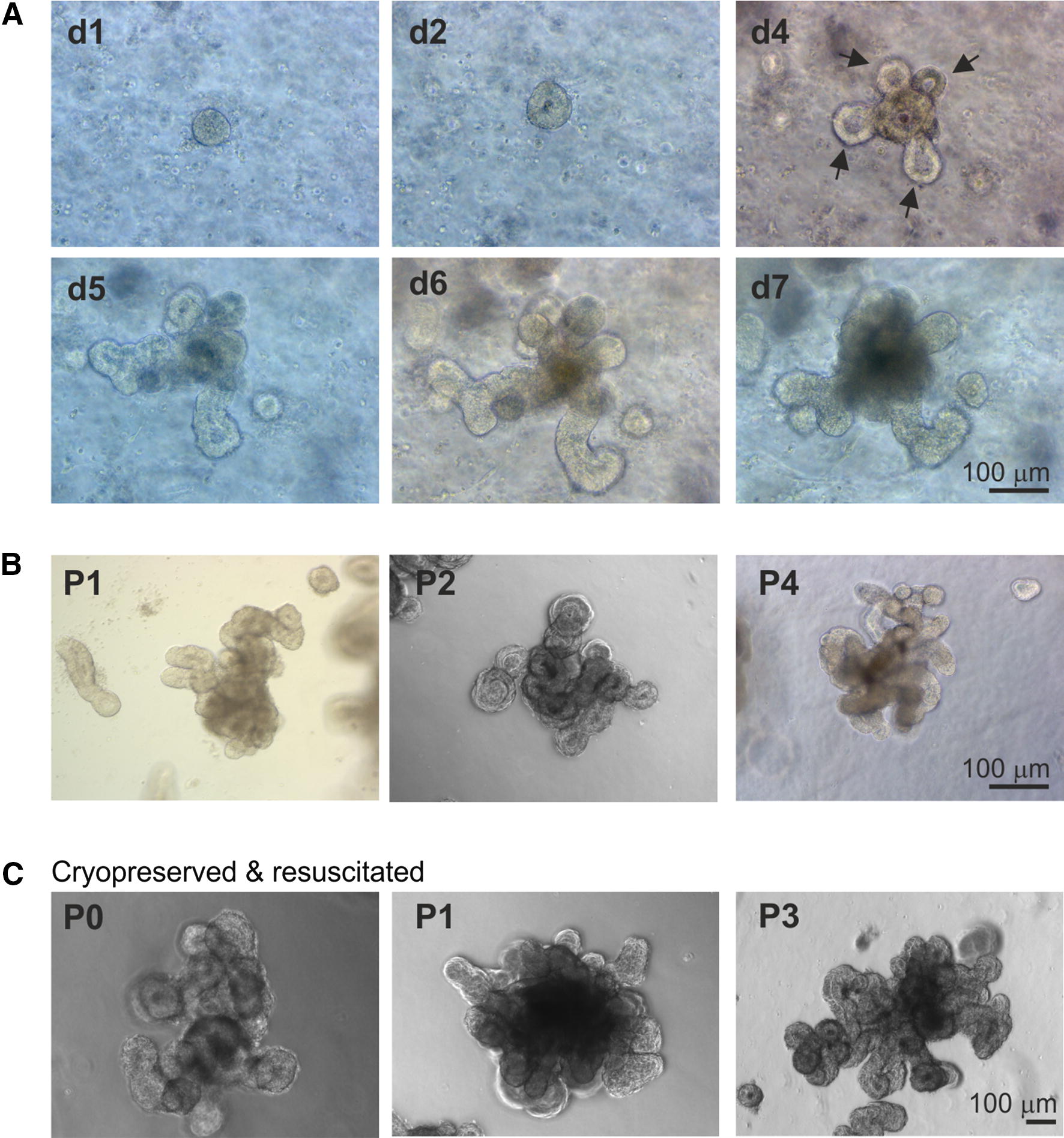
**Bovine enteroids cultivated in the presence of Rho kinase, p38 MAP kinase and TGFβR inhibitors. A** Representative images showing the growth and development of an individual enteroid cultivated in the presence of Rho kinase, p38 MAP kinase and TGFβR inhibitors. Arrows, crypt buds. **B** Representative images showing the development of enteroids following one (P1), two (P2) or four (P4) rounds of serial passage and culture. **C** Representative images showing the development of cryopreserved enteroids following resuscitation and 11 days of culture, and after subsequent rounds of serial passage.

Next, we compared the formation and growth of enteroids in medium containing supplements reported to improve the cultivation of those prepared from human and porcine intestinal crypts [15, 39, 40]. Bovine intestinal crypts cultivated in the presence of Rho-associated kinase inhibitor (Y27632), p38 mitogen-activated protein (MAP) kinase inhibitor (SB202190) and TGF-βR inhibitor (LY2157299) similarly sealed over and formed enterospheres within 24 h, and by day 4 had developed numerous crypt bud-like structures surrounding a central lumen (Figure 2A, arrows). The morphology of the enteroids cultivated in the presence of these inhibitors was distinct from those cultivated in medium alone, as they typically possessed many long and slender crypt domains (Figure 2A). The size of the enteroids and the frequency of the crypt buds increased with the increasing culture duration. Importantly, these enteroids could be serially-passaged after mechanical dissociation and re-plating at a 1:4 density every 7 days (Figure 2B). Using this method, the serial cultivation of enteroids from crypts derived from individual donor calves was achieved for at least 5–8 consecutive passages for a period of up to 2 months without any observable changes to their growth characteristics or morphology.

The positive influence of the inhibitors on the enteroid cultures indicated that Rho-associated kinase, p38MAP kinase and TGF-βR signalling negatively regulate the prolonged maintenance of bovine intestinal epithelial cells. The addition of the Rho-associated kinase inhibitor has been shown to promote the in vitro survival of stem cells by preventing Rho-associated kinase-dependent apoptosis [13, 41–44]. Epithelial–mesenchymal transition (EMT) is an important process during embryonic development and tumour progression during which epithelial cells acquire mesenchymal fibroblast-like properties, and display reduced intercellular adhesion and increased motility [45]. This reduced intercellular adhesion induces apoptosis in the epithelial cells (anoikis). Cultivation in the laminin-rich Matrigel matrix [13] and blockade of p38MAP kinase pathway-mediated EMT in human enteroid cultures help to protect the cells within them against anoikis [15, 46, 47]. Stimulation of enteroid cultures with TGF-β inhibits cell proliferation [48] and its blockade is also essential for the long-term cultivation of human and porcine enteroids [15, 39, 40]. Since signaling via TGF-β inhibits also induces EMT [47], the inhibitors LY2157299 and SB202190 act synergistically in enteroid cultures to block EMT.

### Cryopreservation of bovine organoids

Enteroids were prepared from bovine crypts as above and cultivated in the presence of the Rho kinase, p38 MAP kinase and TGFβR inhibitors. On day 7 of culture whole enteroids were cryopreserved and stored at −155 °C. Afterwards, the cryopreserved enteroid suspensions were then thawed and cultured as above in the presence of inhibitors. Viable enteroids were successfully recovered from the cryopreserved stocks (Figure 2C). Furthermore, the cryopreserved enteroids could also be serially cultivated for at least four consecutive passages (Figure 2C).

### Enteroid morphology

**Figure 3:**
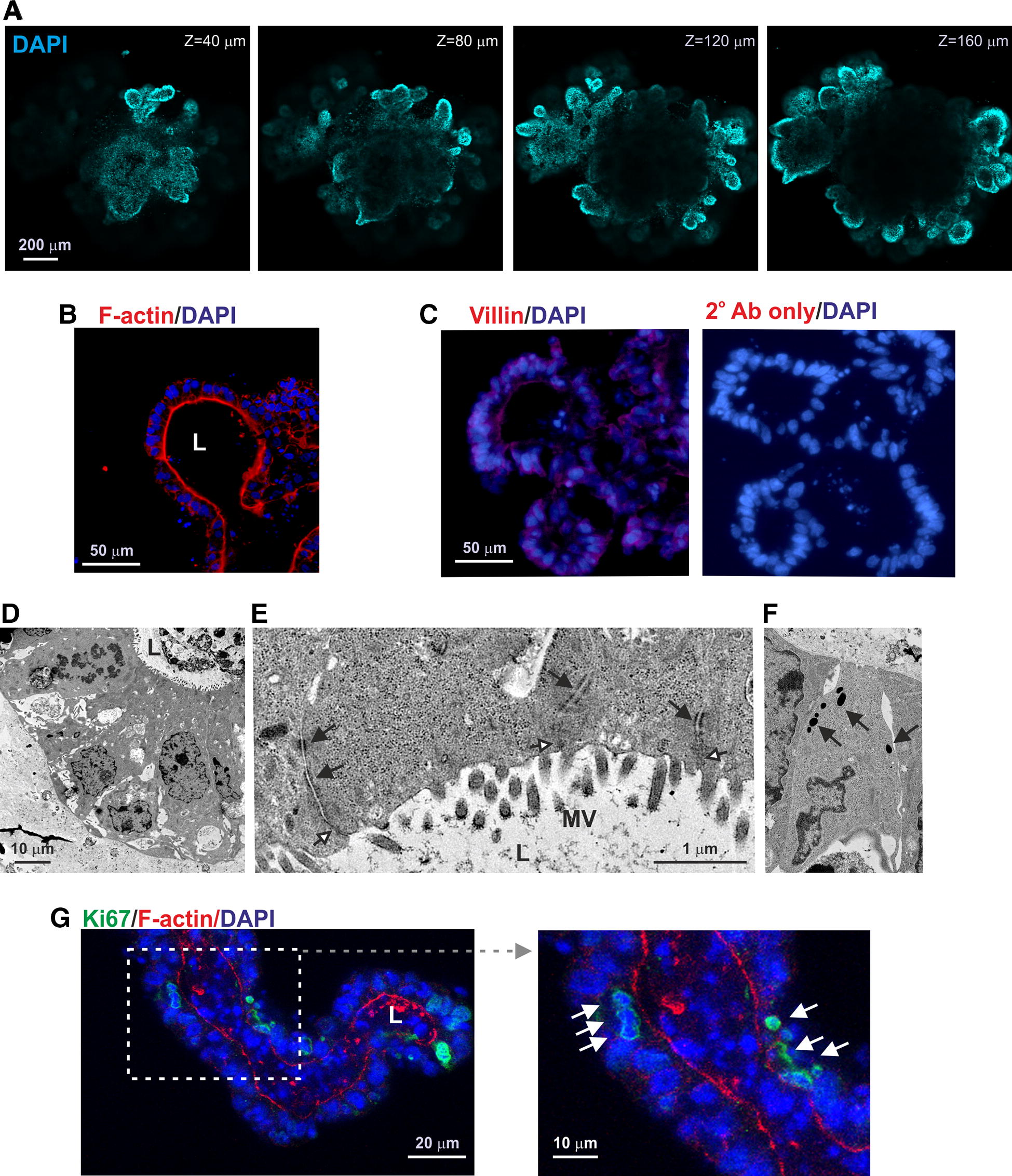
**Histological analysis of bovine enteroids. A** Representative Z axis-projections of enteroids whole-mount stained to detect cell nuclei (DAPI, blue). **B** Detection of F-actin-expressing brush borders at the enteroid lumenal surface (L). **C** IHC analysis of villin expression in bovine enteroids. **D**–**F**, Ulstrastructural analyses of bovine enteroids. **E** Ultrastructural analyses shows the presence of microvilli (MV) on the apical surface of the enterocytes. The enterocytes are sealed by tight junctions (open arrows) and connected by desmosomes (closed arrows). **F** Detection of occasional cells containing dense cytoplasmic vesicles indicative of anti-microbial factor secreting Paneth cells. **G** IHC detect of abundant Ki67^+^ proliferating cells (green).

Histological and ultrastructural analyses confirmed that the enteroids comprised of a single layer of epithelial cells surrounding a continuous lumen (Figures 3A and B; Additional file 1). The lumen of the enteroids was lined with polarized enterocytes with F-actin- and villin-expressing brush borders (Figures 3B and C) and abundant microvilli on their apical surfaces (Figures 3D and E). Ultrastructural analyses revealed that the apical surfaces between the enterocytes were sealed by tight junctions and connected by desmosomes (Figure 3E, open arrows and closed arrows, respectively). Additional analyses revealed the presence of occasional cells containing dense cytoplasmic vesicles indicative of anti-microbial factor secreting Paneth cells (Figure 3F) and abundant Ki67^+^ proliferating cells (Figure 3G).

### Transcriptional analysis of bovine enteroids

**Figure 4:**
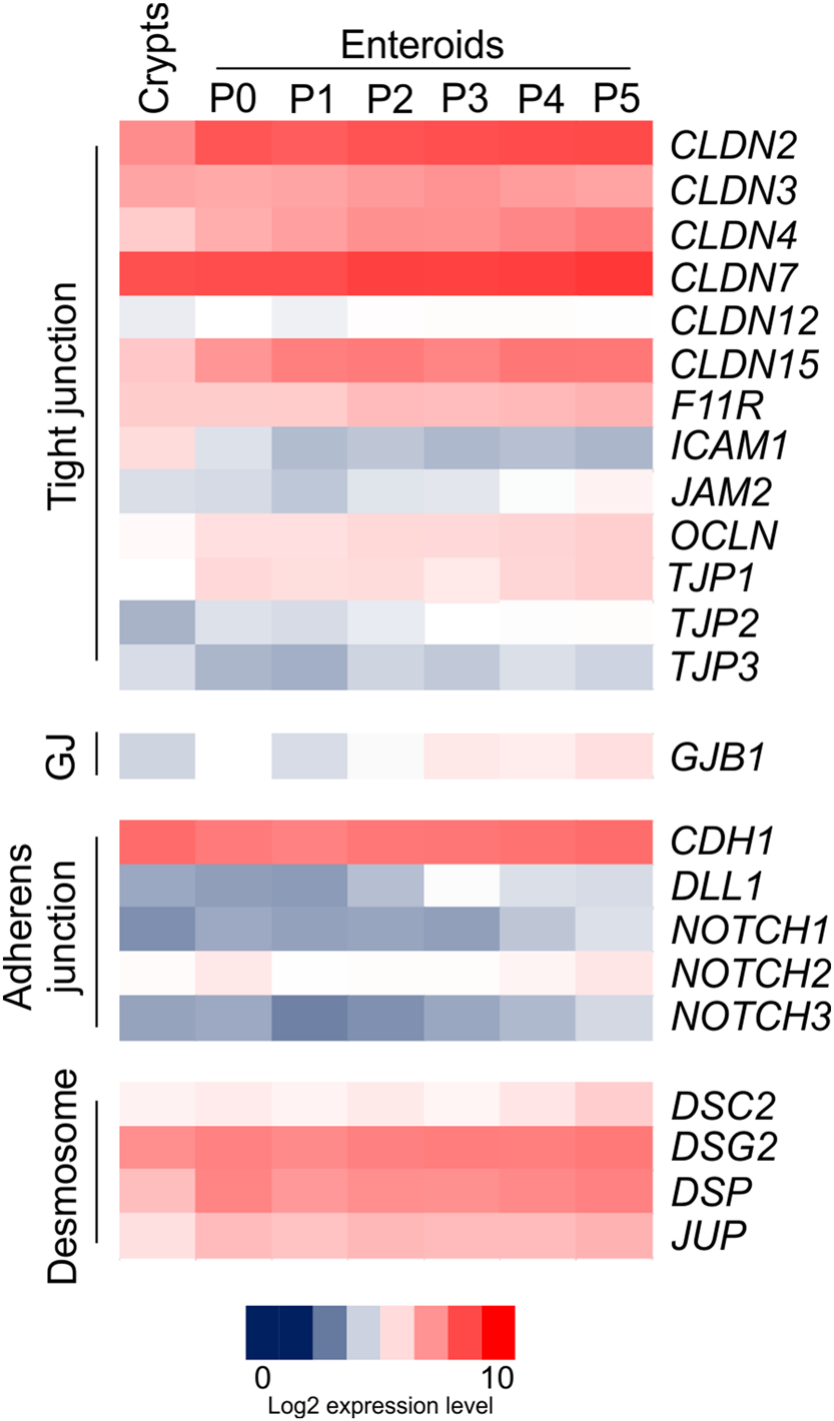
**Expression of mRNA encoding cell junction-related genes in isolated bovine intestinal crypts and enteroids.** Gene expression in isolated crypts and enteroids was compared by mRNA-seq analysis. Heat maps show the relative expression levels (log_2_ transcripts/million reads) of a range of epithelial cell junction-related genes. GJ: gap junction. P: enteroid passage number.

We next extracted mRNA from isolated small intestinal crypts, freshly prepared enteroids (P0) and enteroids after multiple serial passages (P1–5) and compared gene expression in these samples by mRNA-seq. Expression of a wide range of genes encoding components of tight junctions, gap junctions, adherens junctions and desmosomes was detected in the isolated crypts and enteroids data sets (Figure 4), consistent with the detection of these structures by TEM (Figure 3E). The expression of these genes remained similar in the enteroids after serial rounds of passage.

**Figure 5:**
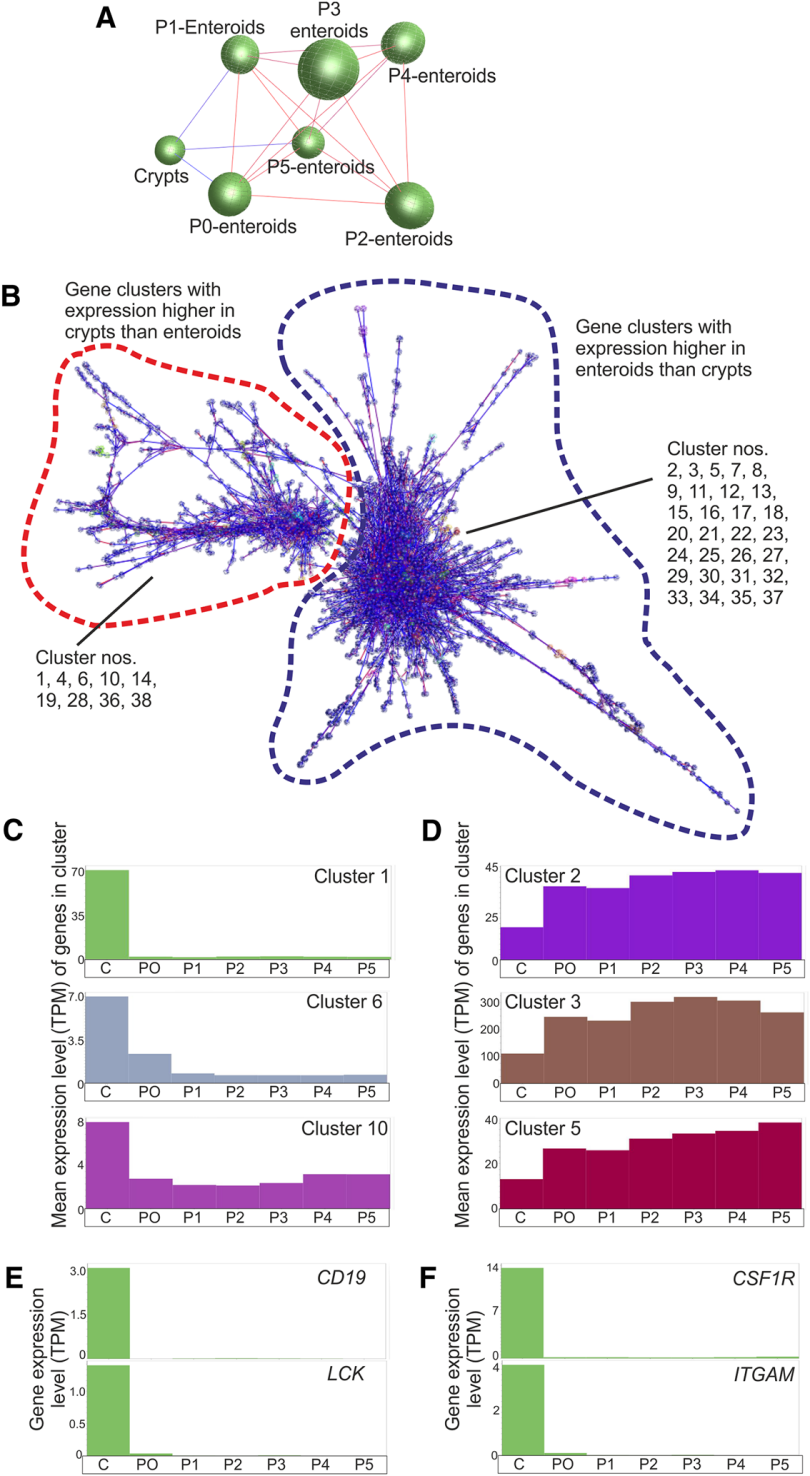
**Network analysis of mRNA-seq expression data derived from isolated bovine intestinal crypts and enteroids. A** Clustering of samples based on their global gene expression profile. A Pearson correlation matrix was prepared by comparing data derived from all seven data sets samples. A network graph was then constructed using sample-to-sample Pearson relationships greater than r ≥ 0.96. Here, the nodes represent individual mRNA-seq data sets. The edges represent the connections between data sets and are coloured according to the strength of the correlation (red, r = 1.0; blue, r ≥ 0.96). P, enteroid passage number. **B** Main component of the network graph derived from all seven data sets samples. Here, the nodes represent transcripts (genes) and the edges represent correlations between individual expression profiles above r ≥ 0.99. Red broken line, region of network graph containing clusters of genes predominantly expressed at higher levels in isolated intestinal crypts. Blue broken line, region of network graph containing clusters of genes predominantly expressed at higher levels in enteroids. **C**, **D** The mean expression profiles of the genes in selected clusters across the seven data sets. The *x* axis shows the samples ordered as follows: C, isolated intestinal crypts; P0, freshly prepared enteroids; P1, passage 1 enteroids, etc. The *y* axis shows the mean expression intensity (transcripts/million reads, TPM) for the cluster. **C** Clusters with mean expression high crypts compared to enteroids. **D** Clusters with mean expression high enteroids compared to crypts. **E**, **F** Comparison of *CD19*, *CSF1R*, *LCK* and *ITGAM* expression across the individual data sets.

The mRNA-seq data sets were next analysed using Miru, a bioinformatics tool which enables the visualisation of network graphs from multiple gene expression data sets, allowing the identification of the relationships between these data sets and the sets of genes that are robustly co-expressed across them [32–34]. The global gene expression profiles of the individual data sets were first compared by performing a sample-to-sample correlation using a Pearson correlation threshold of *r* ≥ 0.96. This analysis showed that all the data sets were included in a single cluster, irrespective of their origin (Figure 5A), suggesting they each share highly similar overall transcriptional profiles. Importantly, this analysis also indicated that the overall transcriptomes of the enteroid cultures remained similar over serial rounds of passage.

Next, a full transcript-to-transcript correlation was performed using a Pearson correlation threshold of *r* ≥ 0.99. This analysis generated a network graph comprising 130 distinct clusters containing ≥ 15 transcripts (Figure 5B). Details of the genes found in each cluster are listed in Additional file 2, and the mean gene expression profiles and representative GO term enrichment annotations for the largest 50 clusters are provided in Additional files 3, 4, respectively. The graph’s structure is derived from the grouping of genes that are correlated in their expressing profiles of *r* ≥ 0.99, and are connected by a large number of edges forming cliques within the network. Clusters of genes with similar expression profiles typically occupied similar regions of the network graph. For example, those clusters which containing genes that were predominantly expressed at higher levels in isolated crypts than in enteroids (e.g. clusters 1, 4, 6, 10, 14) were situated adjacent to each other in the same region of the network graph (Figure 5B, within red broken line; Figure 5C). The clusters that contained genes which were predominantly expressed at higher levels in enteroids (e.g. clusters 2, 3, 5, 7) were similarly situated adjacent to each other in a distinct region of the network graph (Figure 5B, within blue broken line; Figure 5D).

The mean expression profile of the genes within cluster 1 indicated that they were predominantly expressed in isolated intestinal crypts (Figure 5C). Many of the genes present within this cluster are known to be expressed by lymphocytes and leukocytes (GO:Immune response, *P* < 7.83 × 10^−181^; Additional file 4). For example, numerous lymphocyte-related genes such as *CD2*, *CD19*, *LAT*, *LCK* and *ZAP70* (Figure 5E), mononuclear phagocyte-related genes such as *CSF1R*, *ITGAM*, *MAFB* and *SPI1*, as well as several genes encoding the major histocompatibility complex, class II, were represented in this cluster (Figure 5F). Since these genes are not expressed in gut epithelial cells, the expression profiles of these genes across the data sets suggested the presence of low numbers of lymphocytes/leukocytes in the crypt preparations. The expression of these genes was lost upon enteroid cultivation, as shown previously (Figures 5E and F; [49]).

Consistent with the proliferative activity of the crypt buds within the enteroid cultures, many of the enteroid-related clusters were enriched with genes involved in transcriptional regulation and the cell cycle (e.g. cluster 2, GO:RNA binding, *P* < 2.75 × 10^−160^; cluster 3, GO:Ribosomal subunit, *P* < 6.66 × 10^−122^; cluster 15, GO:Cell cycle, *P* < 5.99 × 10^−9^; Additional file 4). Cluster 16, in contrast, was enriched in genes encoding components of the cytoskeleton (GO:Cytoskeleton, *P* < 3.67 × 10^−7^; Additional file 4), whereas clusters 17 and 20 were enriched in mitochondrion-related genes (cluster 17, GO:Mitochondrion, *P* < 1.71 × 10^−7^; cluster 20, GO:Mitochondrial part, *P* < 5.02 × 10^−11^; Additional file 4). Comparison of the mean expression profiles of the genes within the enteroid-related clusters revealed that the expression level of the genes within them was not influenced by serial passage (Figure 5D). This provided additional evidence that the overall transcriptome of the enteroids remained similar during the long-term culture period. Further scrutiny of the enteroid data sets indicated that there was no significant evidence of modulation of the expression level of a range of stress-related genes [13] during the subsequent rounds of passage (Additional file 5).

### Expression of gut epithelial cell sub-population specific genes in bovine enteroid cultures

Little is known of the transcriptomes of the major differentiated cell populations within the bovine intestine, and few cell-specific markers have been identified. mRNA-seq analyses of hundreds of single cells derived from murine crypts and enteroids have been used to characterise the transcriptomes of the distinct cell populations found within the small intestinal epithelium [16–18]. These analyses have helped to identify novel cell markers even for rarer cell types such as enteroendocrine cells [16]. We therefore used the murine intestinal cell lineage-specific gene sets reported in the study by Grün et al. [16], to help gain further insights into the cellular composition of the bovine enteroids. Where annotated homologous bovine genes could be identified in our mRNA-seq data sets, their expression levels in isolated bovine small intestinal crypts and enteroids were compared.

**Figure 6:**
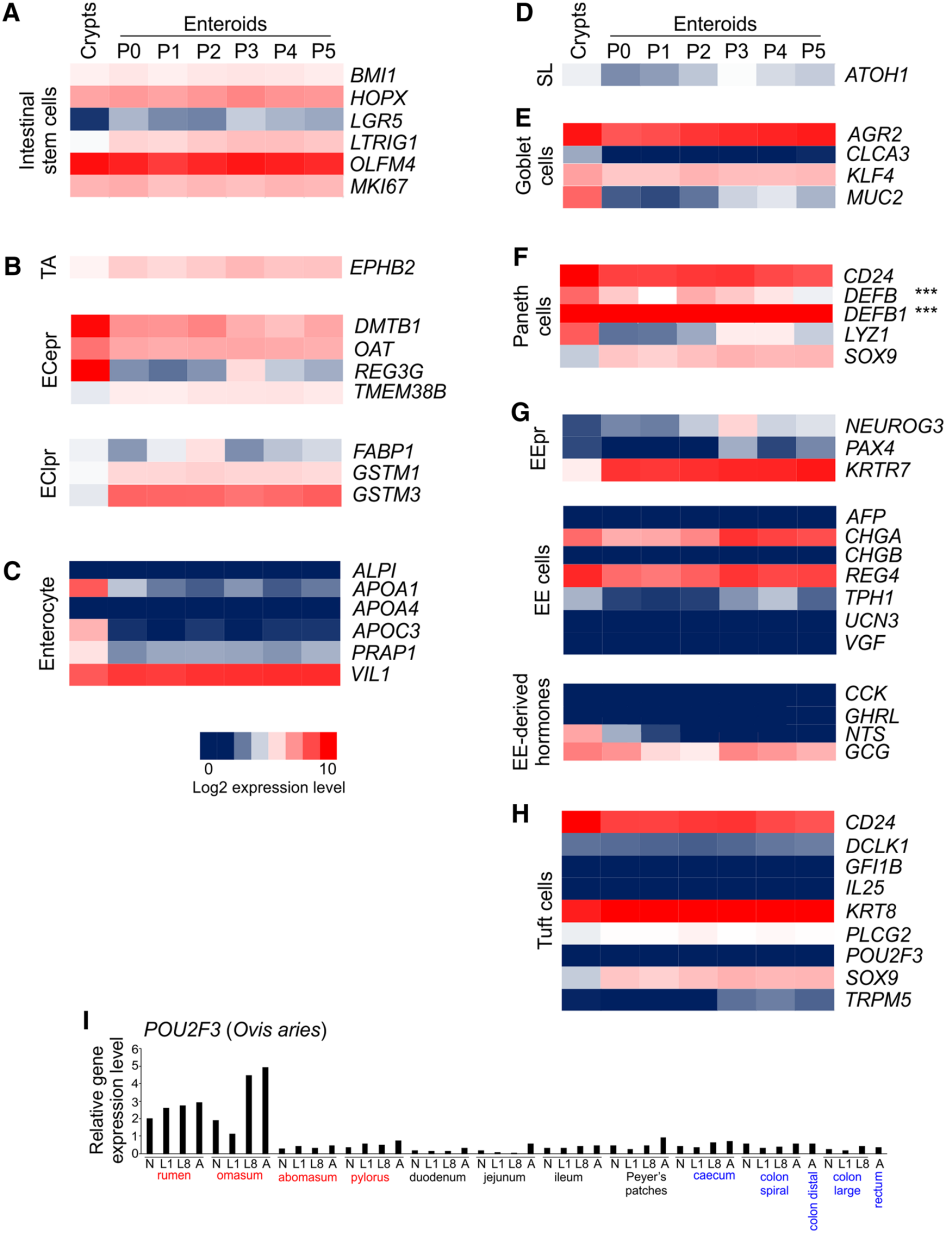
**Expression of intestinal epithelial cell lineage-specific genes in bovine small intestinal crypts and enteroids. A**–**H** Heat maps showing the relative expression levels (log_2_ transcripts/million reads, TPM) of cell lineage-specific genes: **A** intestinal stem cell; **B** transit-amplifying (TA) cells, early enterocyte precursor (ECepr) cells, late enterocyte precursor (EClpr) cells; **C** enterocytes; **D** SL, secretory lineage; **E** goblet cells; **F** Paneth cells; **G** enteroendocrine precursor (EEpr) cells, enteroendocrine (EE) cells, enteroendocrine cell derived hormones; **H** tuft cells. P, enteroid passage number; ***α-defensin genes are not represented in the bovine genome. **I** Expression of *POU2F3* in mRNA-seq data sets derived from distinct regions of the sheep gastrointestinal tract [59]. N, neonate; L1, 1 week old lamb; L8, 8 week old lamb; A, adult.

The enteroid bud structures contain intestinal stem cells that continuously differentiate into all the distinct epithelial cell lineages [1, 13]. Our analyses confirmed abundant expression of the intestinal stem cell-related genes *BMI1*, *HOPX*, *LGR5*, *LTRIG1*, *OLFM4* and *MKI67* in mRNA prepared from our bovine enteroid cultures (Figure 6A). Genes indicative of the presence of transit-amplifying cells, early enterocyte precursor cells and late enterocyte precursor cells were also expressed (Figure 6B). However, although abundant expression of enterocyte-related *VIL1* (encoding villin) was observed in crypts and enteroids (consistent with our IHC analysis; Figure 3C), the expression of a range of other enterocyte-related genes was low or absent (Figure 6C). Whether these differences are due to species-specific differences between bovine and murine enterocytes, or are a consequence of the effects of in vitro cultivation are uncertain.

In mice and humans expression of the transcription factor ATOH1 is required for the differentiation of epithelial progenitor cells into secretory lineages including goblet cells, Paneth cells, enteroendocrine cells and tuft cells [50, 51]. The bovine homologue of *ATOH1* was also expressed in the crypt and enteroid data sets (Figure 6D). In addition to this, the subsequent expression of KLF4 is required for goblet cell maturation [52]. The expression of *KLF4*, *AGR2* and *MUC2* (encoding mucin 2) in the enteroid data sets implied presence of mucin-secreting goblet cells in these cultures (Figure 6E).

Murine studies show that expression of the transcription factor SOX9 is important for Paneth cell maturation [53, 54]. Paneth cells within the base of murine and human intestinal crypts release antimicrobial products such as lysozyme which help to prevent bacterial translocation across the gut epithelium to protect the crypts from bacterial infection [6]. The expression of *SOX9*, *CD24* and *LYZ1* in the crypt and enteroids suggested the presence of Paneth cells (Figure 6F). Murine Paneth cells also express α-defensins which have selective antimicrobial activity against pathogenic bacteria [4]. Although the bovine genome does not contain α-defensin genes to our knowledge, a range of β-defensin genes (e.g. *DEFB*, *DEFB1*) were expressed in the crypt and enteroid data sets implying that they may play a similar role in the bovine intestine.

Enteroendocrine cells are a heterogeneous population of cells that help to control metabolism through the secretion of distinct hormones [16, 55–57]. The maturation of enteroendocrine cells in mice is dependent upon the expression of neurogenin 3 [58], and the expression of *NEUROG3* in the enteroid cultures implied the presence of enteroendocrine precursor cells (Figure 6G). Furthermore, abundant expression of the enteroendocrine cell-related genes *CHGA* (encoding chromogranulin A) and *REG4* was also detected. Individual enteroendocrine cells differ in the range of hormones they express. Analysis of genes encoding several enteroendocrine cell-derived hormones revealed specific expression of glucagon (*GCG*) in the bovine crypts and enteroids. Expression of neurotensin (*NTS*) was detected in the crypts which was lost upon enteroid cultivation suggesting that the culture conditions were unable to support the differentiation of neurotensin-secreting enteroendocrine cells.

Tuft cells are a rare epithelial cell population which help initiate type 2 mucosal immunity to helminth parasites through secretion of interleukin (IL)-25 [20]. In mice the differentiation of tuft cells is dependent upon expression of the transcription factor POU2F3 [20]. However, the crypt and enteroid cultures lacked detectable expression of *POU2F3* and other tuft cell-related genes including *DCLK1*, *GFI1B*, *IL25* and *TRPM5* [20] suggesting little if any representation of these cells (Figure 6H). The density of tuft cells is very low in the intestinal epithelium in the steady-state, but their abundance significantly increases upon helminth infection. These cells are similarly rare in the epithelium of murine enteroids, but expand upon stimulation with IL-4 and/or IL-13 [20]. Whether tuft cells can be also be induced in bovine enteroids remains to be determined. Differences between ruminant and monogastric species in the representation of these cells in the ileum also cannot be excluded. A comparison of *POU2F3* expression in mRNA-seq data derived from distinct regions of the sheep gastrointestinal tract [59] shows relatively high expression of this gene in the rumen and omasum, but little if any expression throughout the small and large intestines (Figure 6I).

## Discussion

Here we describe a simple and reliable procedure to establish in vitro enteroid cultures from bovine small intestinal (ileal) crypts. Furthermore, we show that these enteroid cultures can be successfully maintained long-term through multiple serial passages with little influence on their growth characteristics, morphology or transcriptome. The ability to serially cultivate and expand these enteroid cultures has important ethical implications as they reduce the reliance upon tissues from multiple donor calves to establish additional batches of enteroids for individual sets of experiments. We also demonstrate that it is possible to cryopreserve these bovine enteroids and recover viable cultures from frozen stocks after resuscitation, as has been shown in other host species [39, 43, 60]. Thus large numbers of enteroids could be prepared and expanded from the intestine of a single calf and then cryopreserved for future use in many independent studies.

Substantial progress has been made in the identification of genes and markers that can identify specific murine and human intestinal cell lineages. Unfortunately the limited availability of useful IHC reagents such monoclonal or polyclonal antibodies with specificity to bovine cells has hindered the identification of similar markers in the bovine intestine. However, independent research groups have shown how detailed mRNA-seq analyses of the transcriptomes of individual enteroid cells and cell lineages in the murine intestine, can help identify novel epithelial cell markers [16–18]. Gene expression analyses of our bovine enteroid cultures indicated that they comprised a mixed population of intestinal epithelial cell lineages including intestinal stem cells, enterocytes, Paneth cells, goblet cells and enteroendocrine cells.

Enteroid cultures have been shown to have useful practical application in the study of the signalling pathways which modulate the differentiation and maintenance of distinct epithelial cell lineages in the murine intestine including Lgr5^+^ intestinal stem cells [6, 61], tuft cells [20] and antigen-sampling M cells [3, 19, 21, 22]. The 3D nature of the enteroids with a layer of epithelial cells surrounding a central, closed, lumen has also been shown to be an effective system in which to model mucosal permeability [62] and the interactions of mucosally-acquired pathogenic bacteria [24], viruses [25] and protozoa [26] with the gut epithelium. Our data suggest that these 3D bovine enteroid cultures represent a novel, physiologically-relevant and tractable in vitro system in which epithelial cell differentiation and function, and host–pathogen interactions in the bovine small intestine can be studied.

## Additional files

**Additional file 1.** **Video projection showing Z axis-projection of an enteroid whole-mount stained to detect cell nuclei.** Video projection showing Z axis-projection of an enteroid whole-mount stained to detect cell nuclei. DAPI, blue.

**Additional file 2.** **Details of the genes found in each co-expression cluster derived from the mRNA-seq analysis data.** Table providing details of the genes found in each co-expression cluster in the network graph derived from the mRNA-seq analysis data.

**Additional file 3.** **Mean expression profiles of the genes in each of the largest 50 co-expression clusters.** Individual mean expression profiles of the genes in each of the largest 50 co-expression clusters derived from the network graph. The *x* axis shows the samples ordered as follows: C, isolated intestinal crypts; P0, freshly prepared enteroids; P1, passage 1 enteroids, etc. The *y* axis shows the mean expression intensity (transcripts/million reads, TPM) for the cluster.

**Additional file 4.** **Representative GO term enrichment annotations for the genes in the largest 50 co-expression clusters.** Table listing the representative GO term enrichment annotations for the genes in the largest 50 co-expression clusters derived from the network graph.

**Additional file 5.** **Comparison of stress-related gene expression in bovine enteroid cultures.** Table comparing the relative expression level of a range of stress-related genes [13] in the enteroid cultures during serial subsequent rounds of passage. P0, freshly prepared enteroids; P1, passage 1 enteroids, etc.

## Abbreviations

EGF: epidermal growth factor
EMT: epithelial–mesenchymal transition
GO: gene ontology
IHC: immunohistochemistry
LGR5: leucine-rich repeat-containing G protein-coupled receptor 5
MAP: mitogen-activated protein
RANK: cytokine receptor activator of NF-κB
RANKL: receptor activator of NF-κB ligand
REG4: regenerating islet-derived family member 4
TEM: transmission electron microscopy
TGF: transforming growth factor
TPM: transcripts/million reads
