# Supplementary material for: Development of in vitro enteroids derived from bovine small intestinal crypts
Source: Vet Res. 2018 Jul 3;49:54. doi: 10.1186/s13567-018-0547-5 (PMC6029049; doi:10.1186/s13567-018-0547-5)
Supplement: Supplementary file 3 — Additional file 3. Mean expression profiles of the genes in each of the largest 50 co-expression clusters. Individual mean expression profiles of the genes in each of the largest 50 co-expression clusters derived from the network graph. The x axis shows the samples ordered as follows: C, isolated intestinal crypts; P0, freshly prepared enteroids; P1, passage 1 enteroids, etc. The y axis shows the mean expression intensity (transcripts/million reads, TPM) for the cluster. [file 13567_2018_547_MOESM3_ESM.pdf]

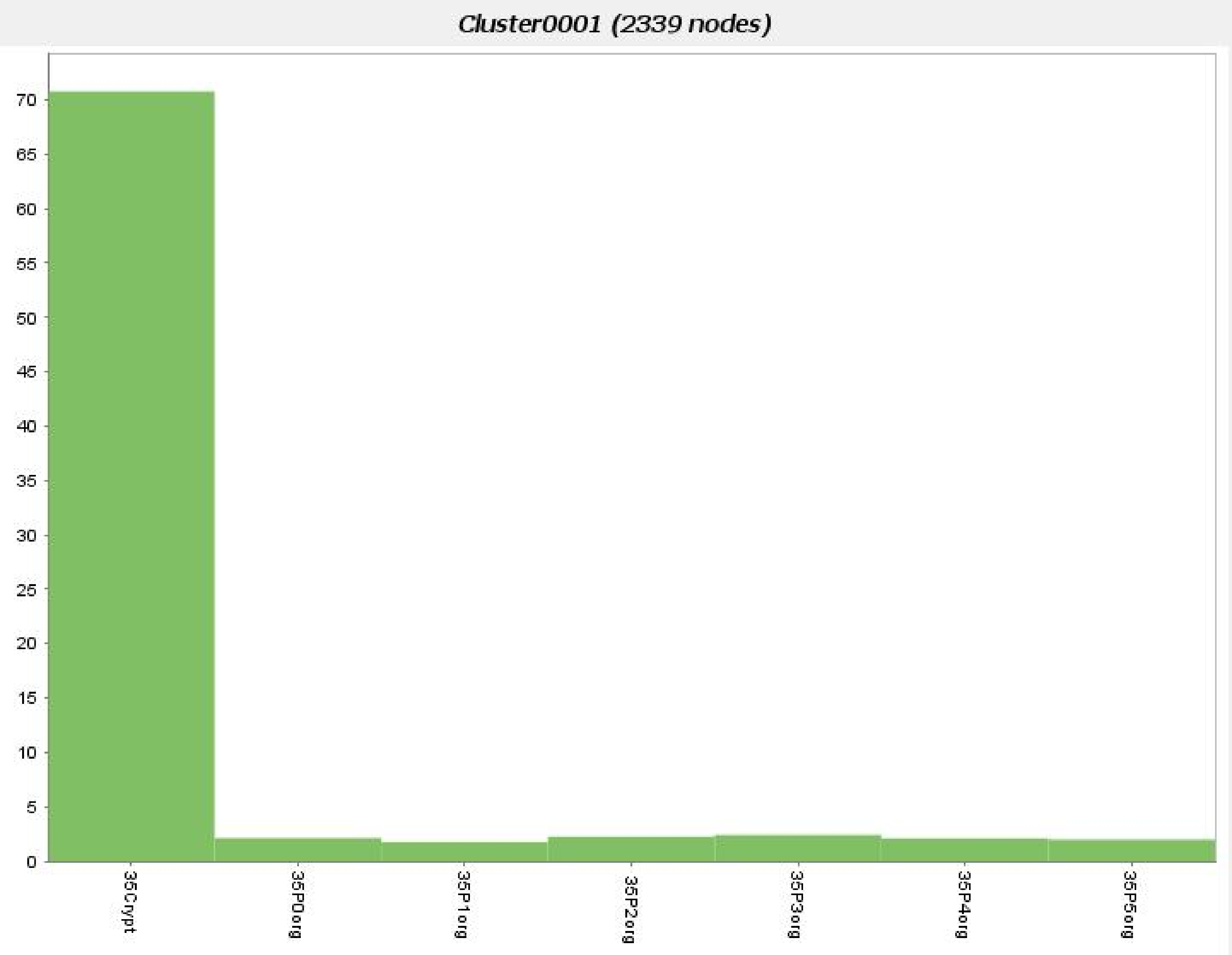
Cluster0001 (2339 nodes)
70
65
60
55
50
45
40
35
30
25
20
15
10
5
0
35Crypt
35P0org
35P1org
35P2org
35P3org
35P4org
35P5org

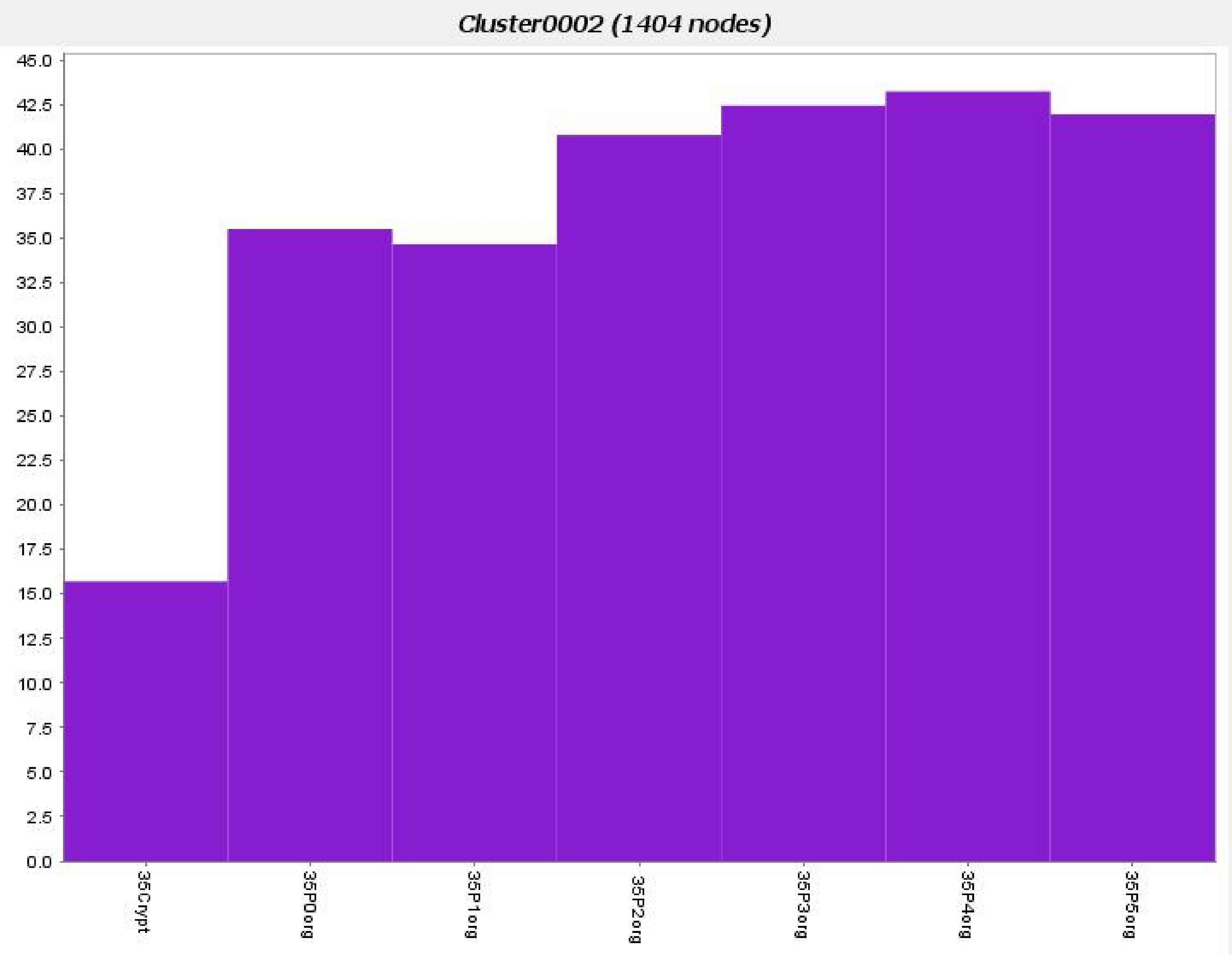
Cluster0002 (1404 nodes)
45.0
42.5
40.0
37.5
35.0
32.5
30.0
27.5
25.0
22.5
20.0
17.5
15.0
12.5
10.0
7.5
5.0
2.5
0.0
35Crypt
35P0org
35P1org
35P2org
35P3org
35P4org
35P5org

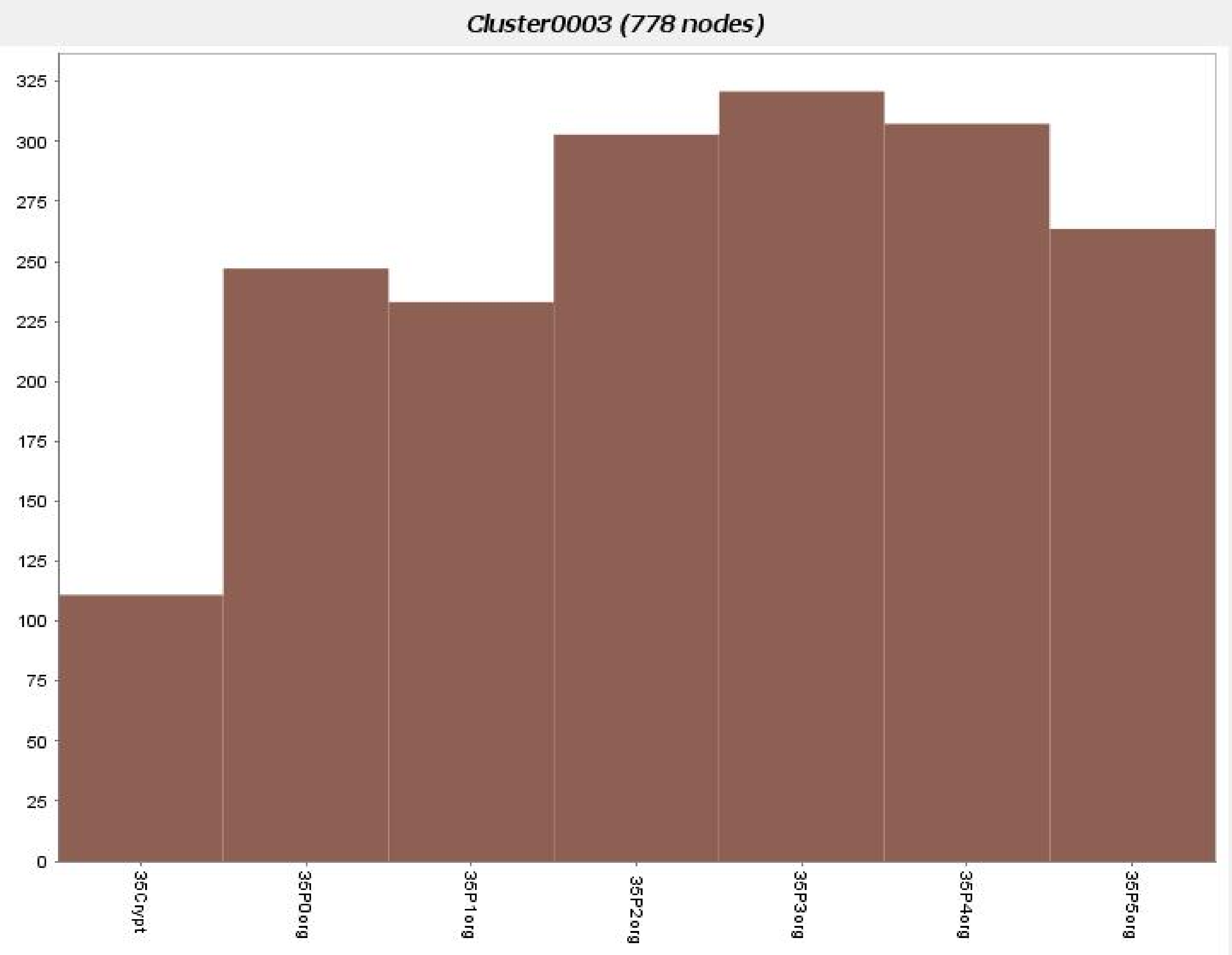

Cluster0003 (778 nodes)
325
300
275
250
225
200
175
150
125
100
75
50
25
0
35Crypt
35P0org
35P1org
35P2org
35P3org
35P4org
35P5org

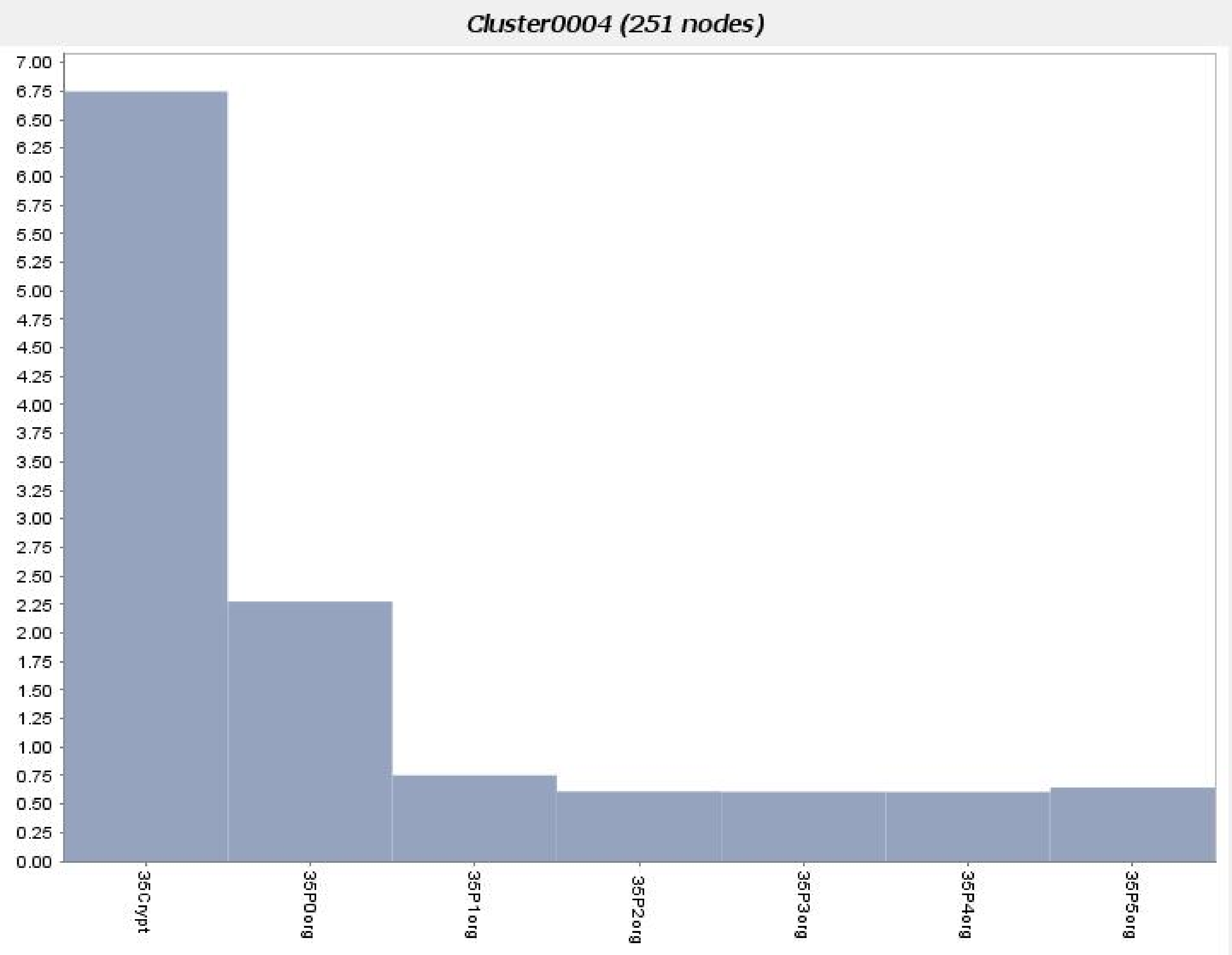
Cluster0004 (251 nodes)
7.00
6.75
6.50
6.25
6.00
5.75
5.50
5.25
5.00
4.75
4.50
4.25
4.00
3.75
3.50
3.25
3.00
2.75
2.50
2.25
2.00
1.75
1.50
1.25
1.00
0.75
0.50
0.25
0.00
35Crypt
35P0org
35P1org
35P2org
35P3org
35P4org
35P5org

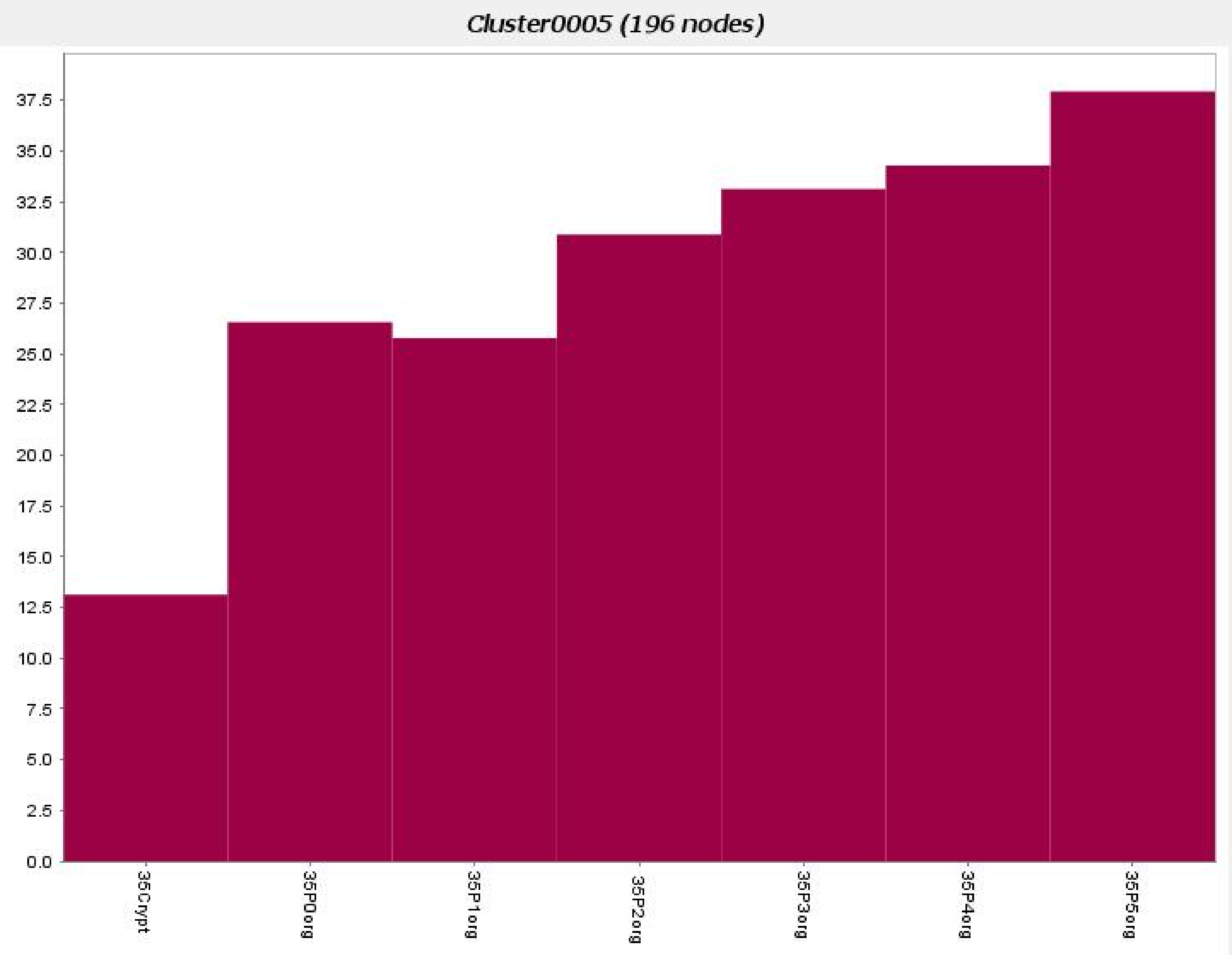
Cluster0005 (196 nodes)
37.5
35.0
32.5
30.0
27.5
25.0
22.5
20.0
17.5
15.0
12.5
10.0
7.5
5.0
2.5
0.0
35Crypt
35P0org
35P1org
35P2org
35P3org
35P4org
35P5org

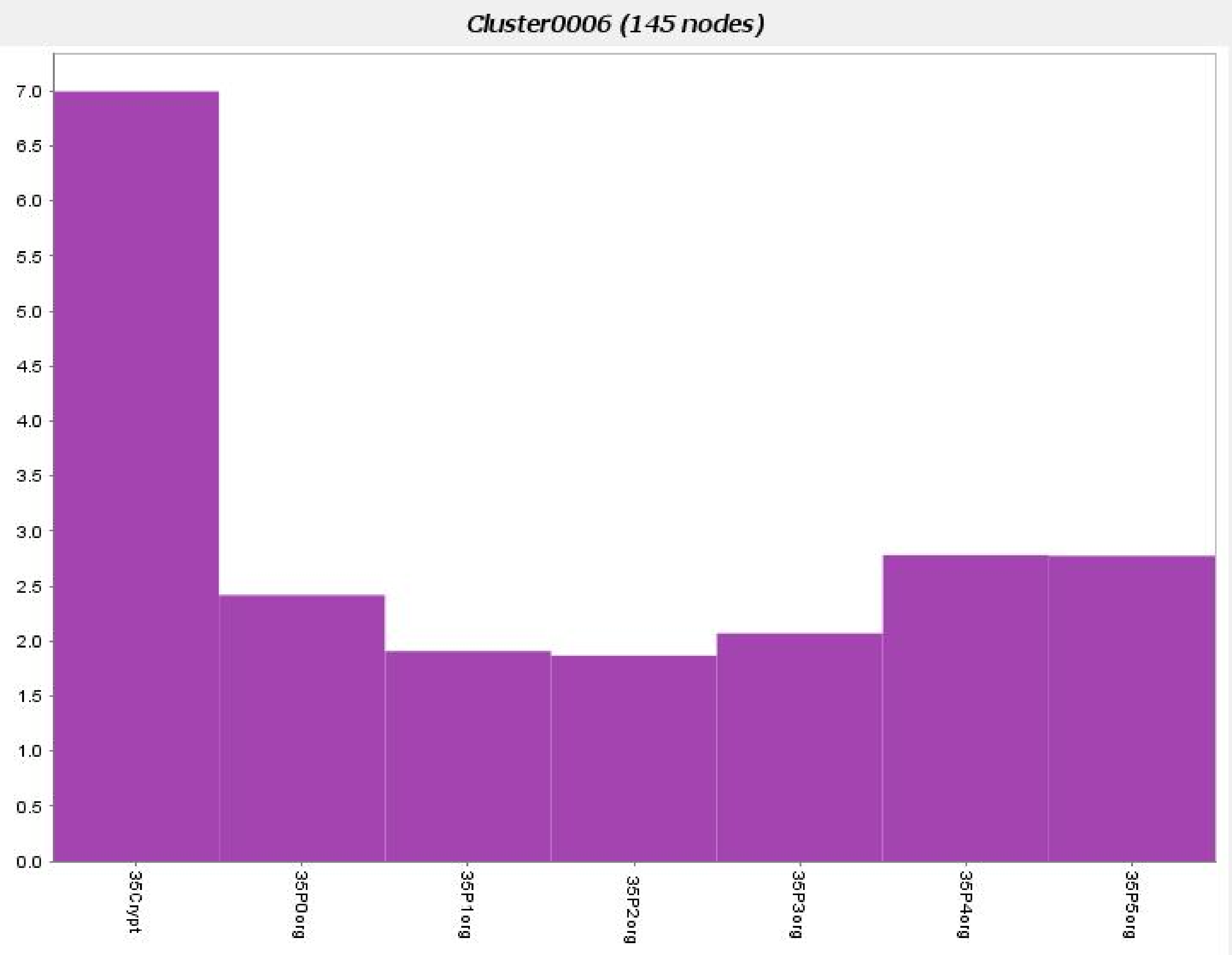
Cluster0006 (145 nodes)
7.0
6.5
6.0
5.5
5.0
4.5
4.0
3.5
3.0
2.5
2.0
1.5
1.0
0.5
0.0
35Crypt
35P0org
35P1org
35P2org
35P3org
35P4org
35P5org

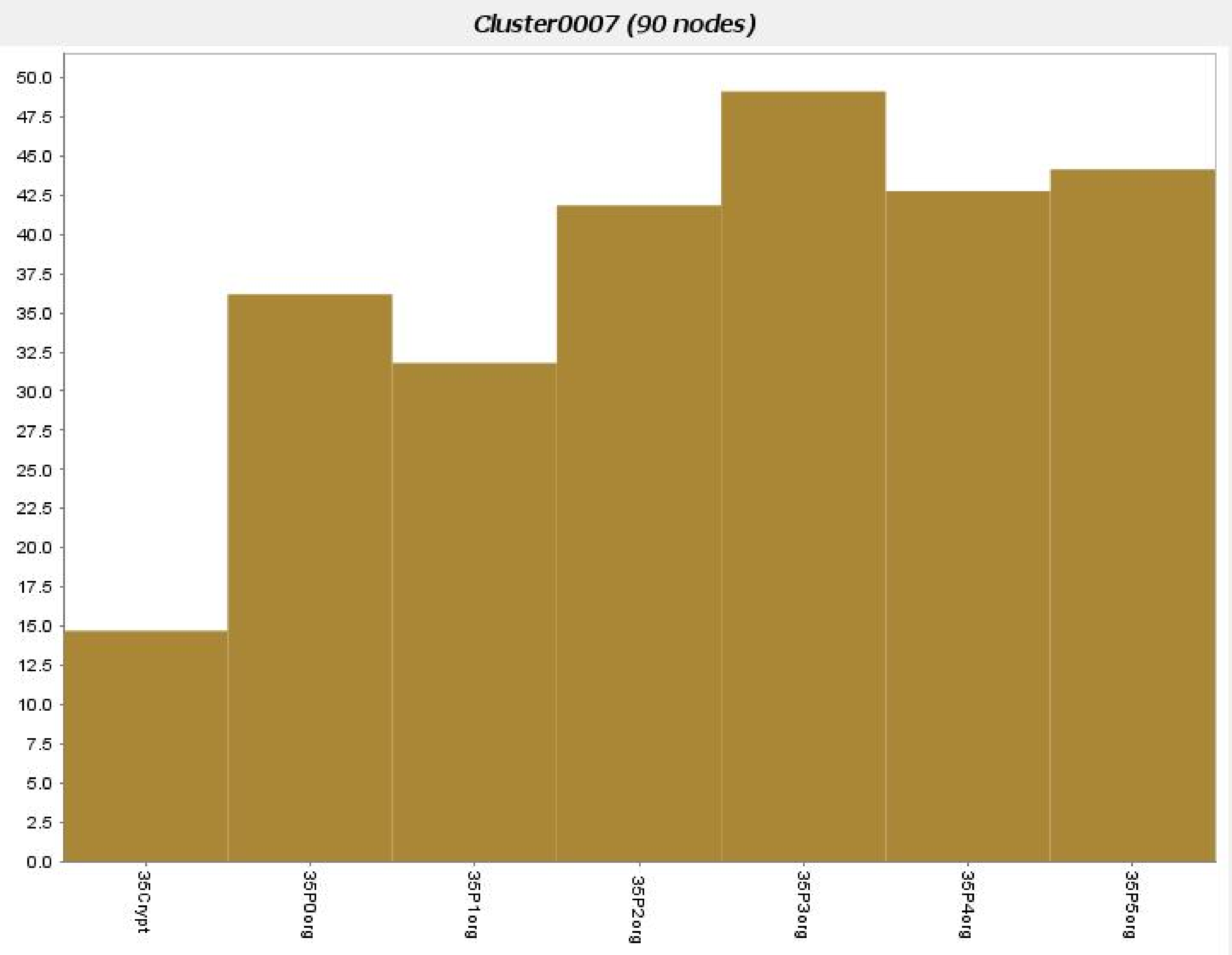
Cluster0007 (90 nodes)
50.0
47.5
45.0
42.5
40.0
37.5
35.0
32.5
30.0
27.5
25.0
22.5
20.0
17.5
15.0
12.5
10.0
7.5
5.0
2.5
0.0
35Crypt
35P0org
35P1org
35P2org
35P3org
35P4org
35P5org

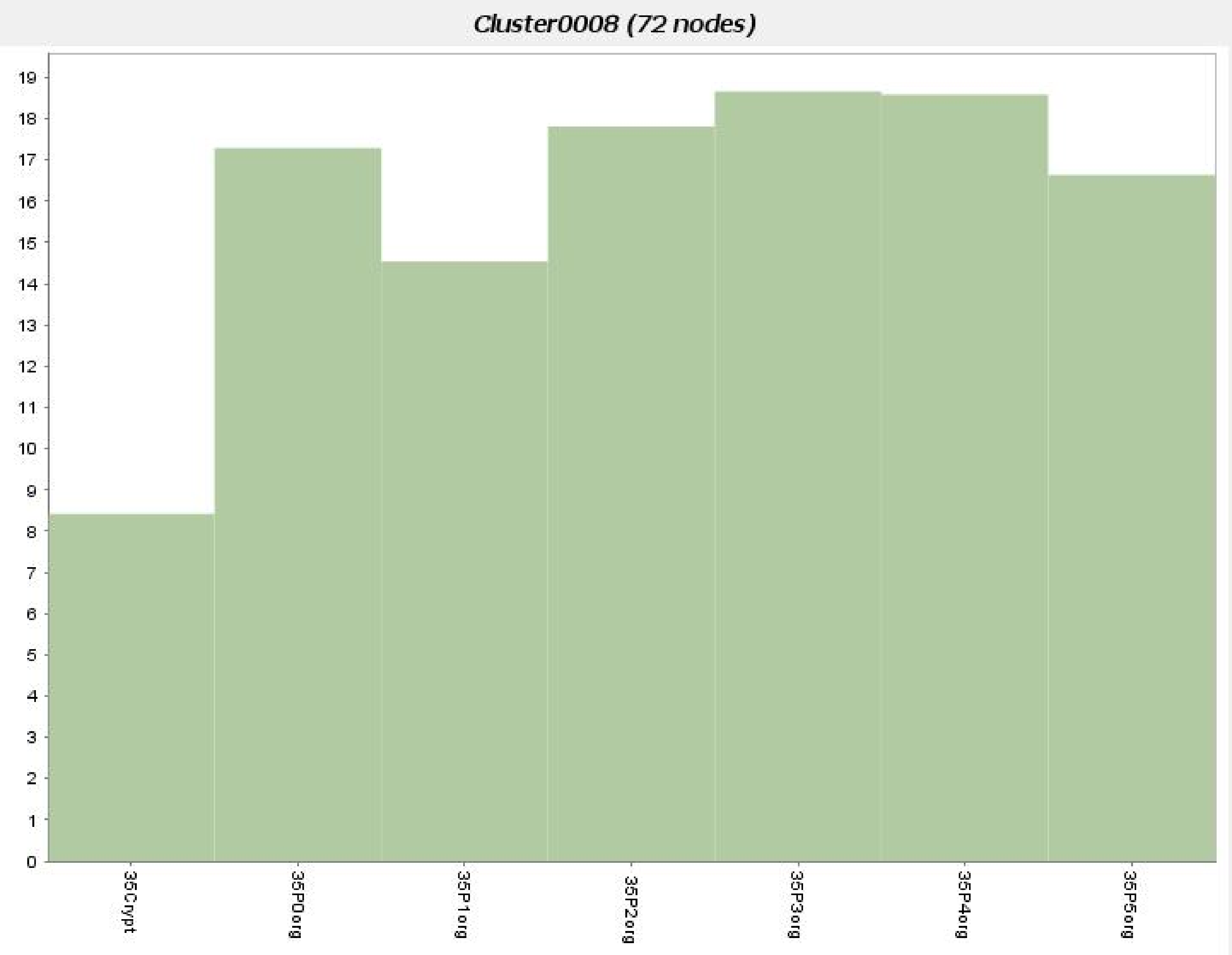

Cluster0008 (72 nodes)
19
18
17
16
15
14
13
12
11
10
9
8
7
6
5
4
3
2
1
0
35Crypt
35P0org
35P1org
35P2org
35P3org
35P4org
35P5org

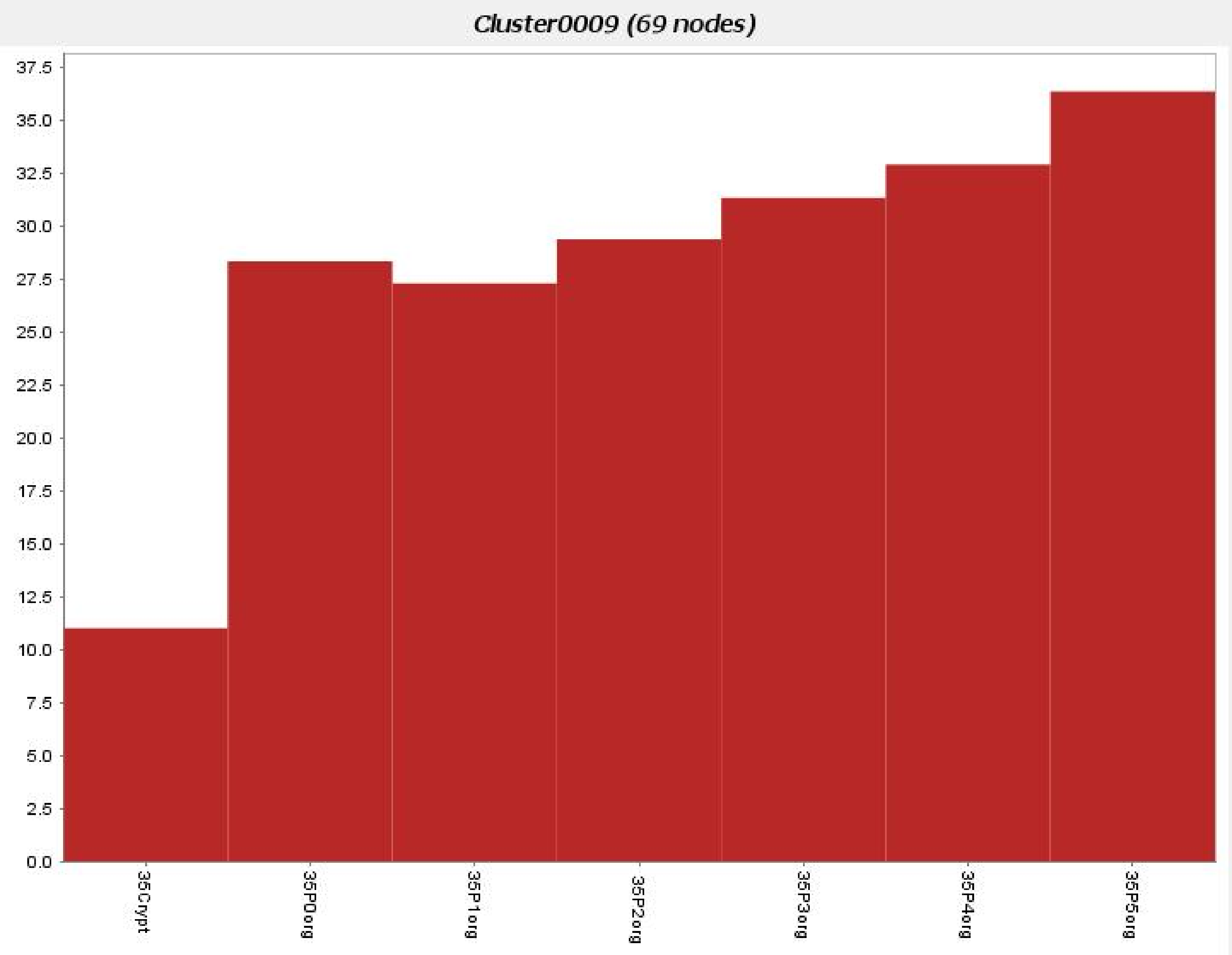

Cluster0009 (69 nodes)
37.5
35.0
32.5
30.0
27.5
25.0
22.5
20.0
17.5
15.0
12.5
10.0
7.5
5.0
2.5
0.0
35Crypt
35P0org
35P1org
35P2org
35P3org
35P4org
35P5org

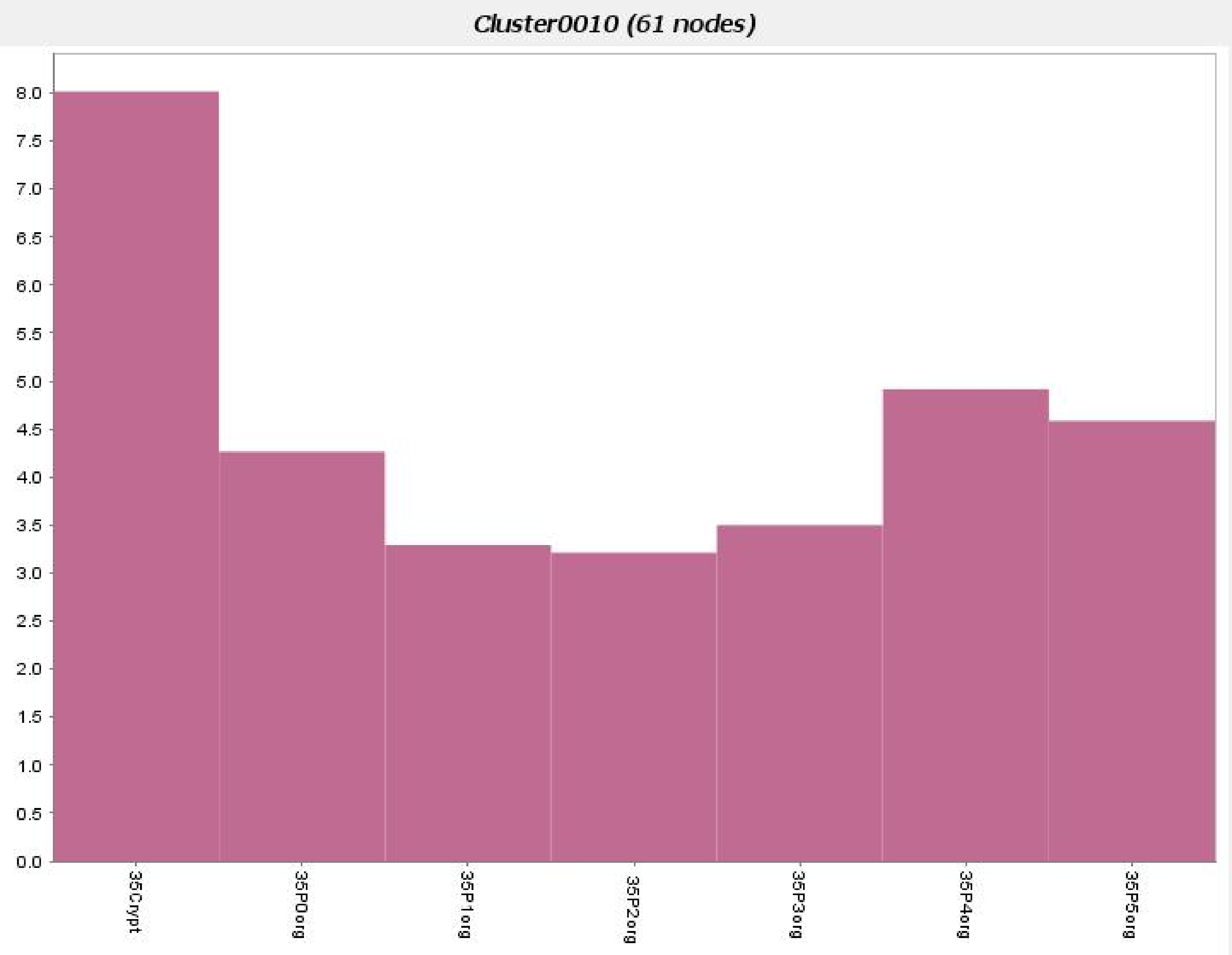

Cluster0010 (61 nodes)
8.0
7.5
7.0
6.5
6.0
5.5
5.0
4.5
4.0
3.5
3.0
2.5
2.0
1.5
1.0
0.5
0.0
35Crypt
35P0org
35P1org
35P2org
35P3org
35P4org
35P5org

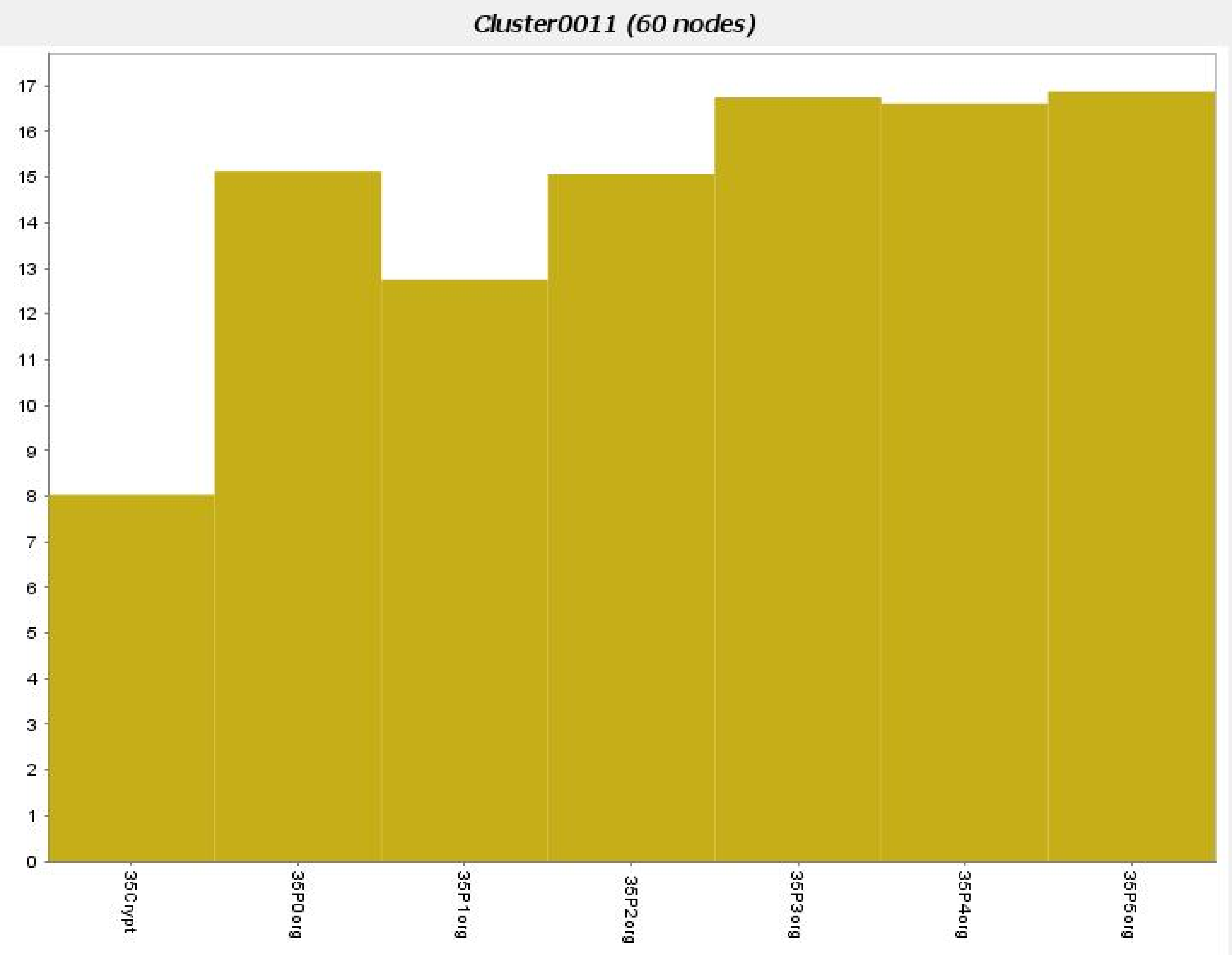

Cluster0011 (60 nodes)
17
16
15
14
13
12
11
10
9
8
7
6
5
4
3
2
1
0
35Crypt
35P0org
35P1org
35P2org
35P3org
35P4org
35P5org

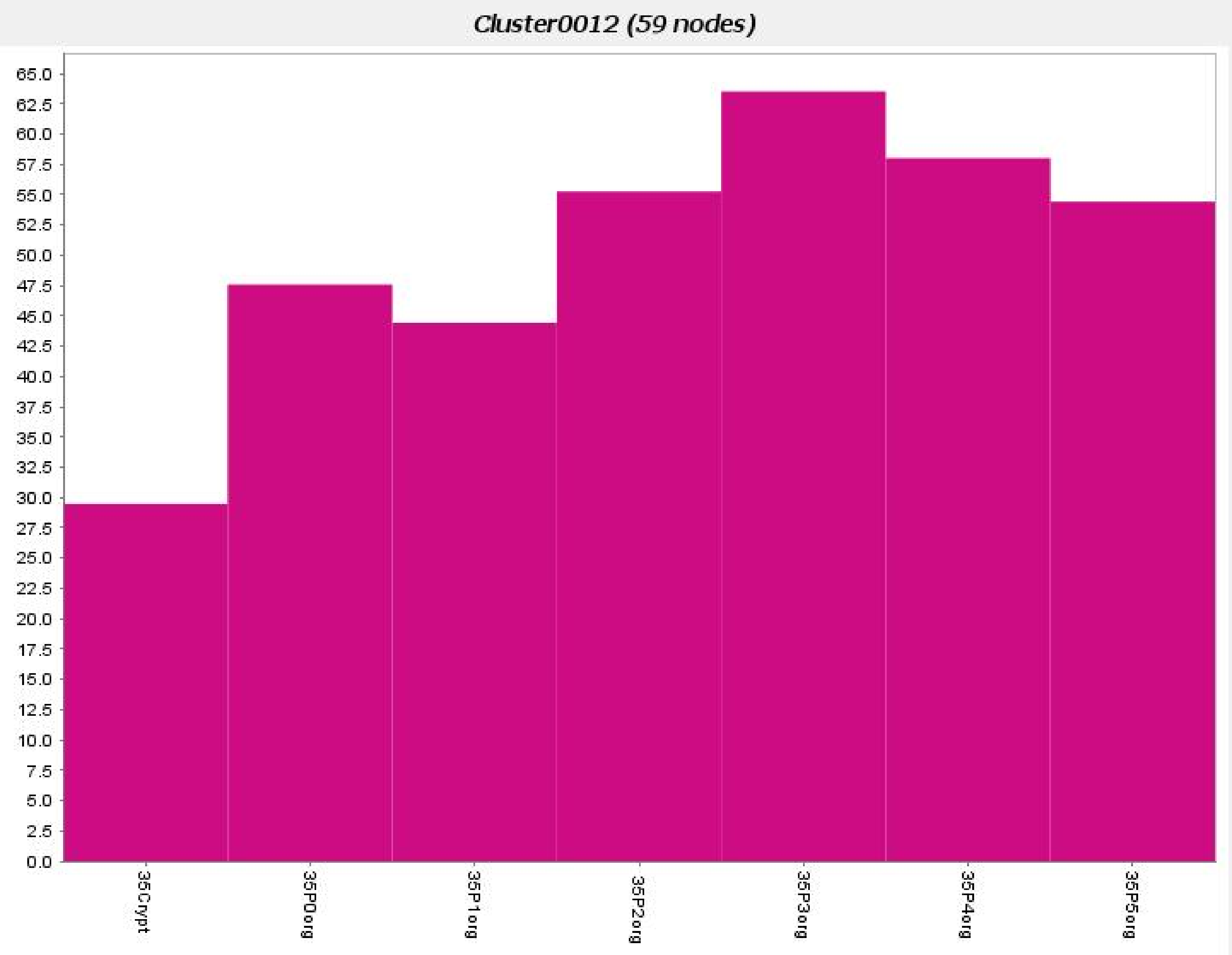
Cluster0012 (59 nodes)
65.0
62.5
60.0
57.5
55.0
52.5
50.0
47.5
45.0
42.5
40.0
37.5
35.0
32.5
30.0
27.5
25.0
22.5
20.0
17.5
15.0
12.5
10.0
7.5
5.0
2.5
0.0
35Crypt
35P0org
35P1org
35P2org
35P3org
35P4org
35P5org

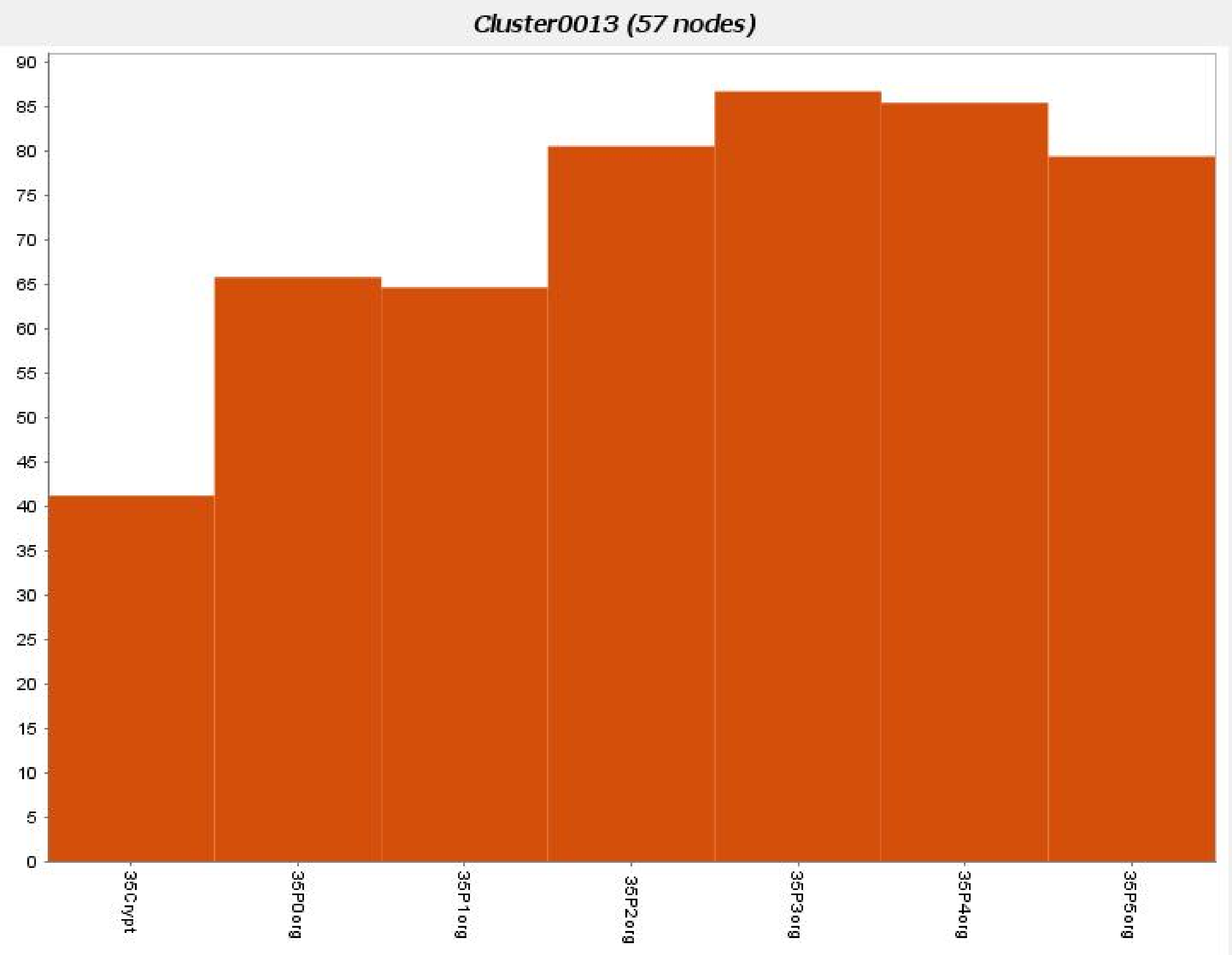
Cluster0013 (57 nodes)
90
85
80
75
70
65
60
55
50
45
40
35
30
25
20
15
10
5
0
35Crypt
35P0org
35P1org
35P2org
35P3org
35P4org
35P5org

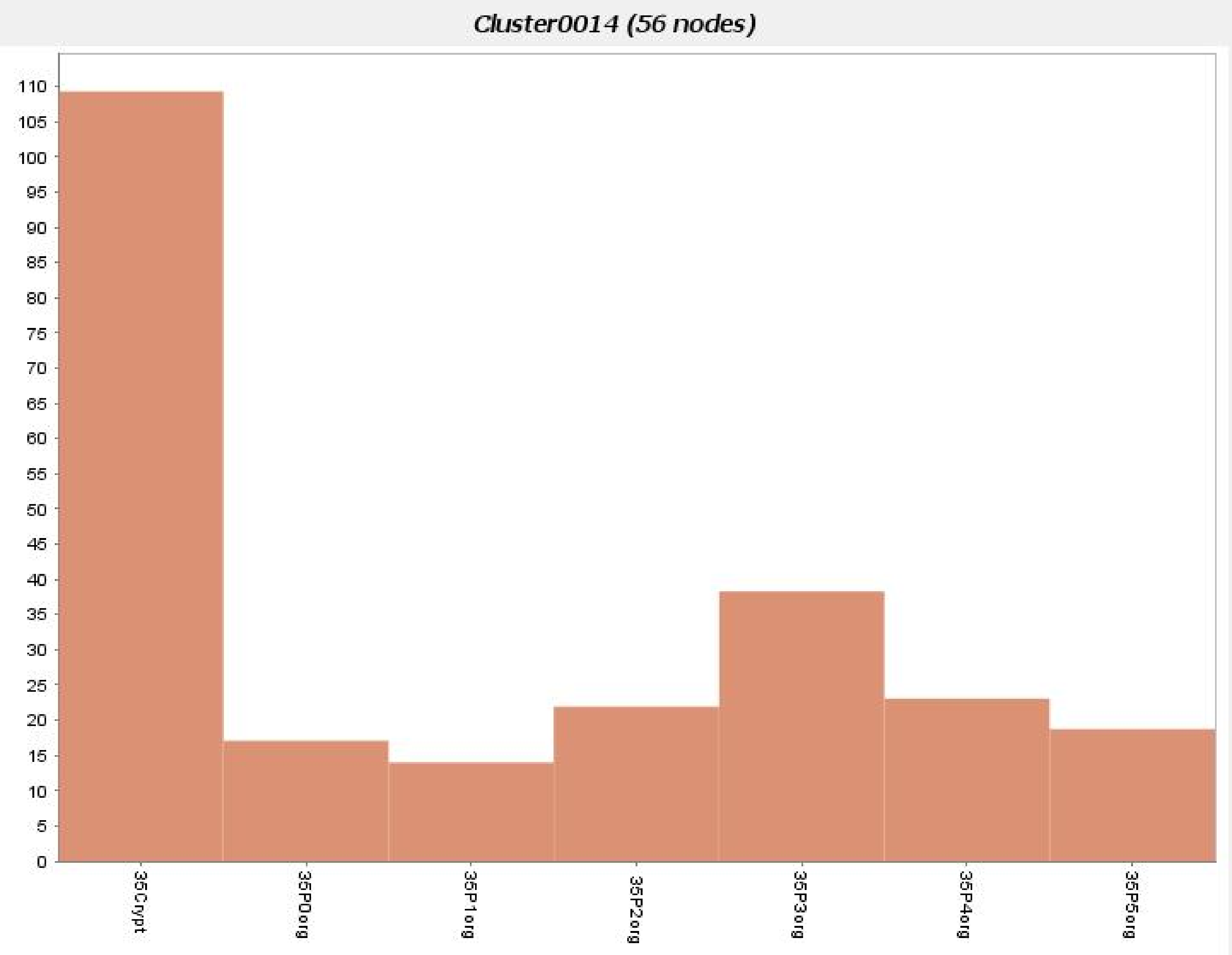
Cluster0014 (56 nodes)
110
105
100
95
90
85
80
75
70
65
60
55
50
45
40
35
30
25
20
15
10
5
0
35Crypt
35P0org
35P1org
35P2org
35P3org
35P4org
35P5org

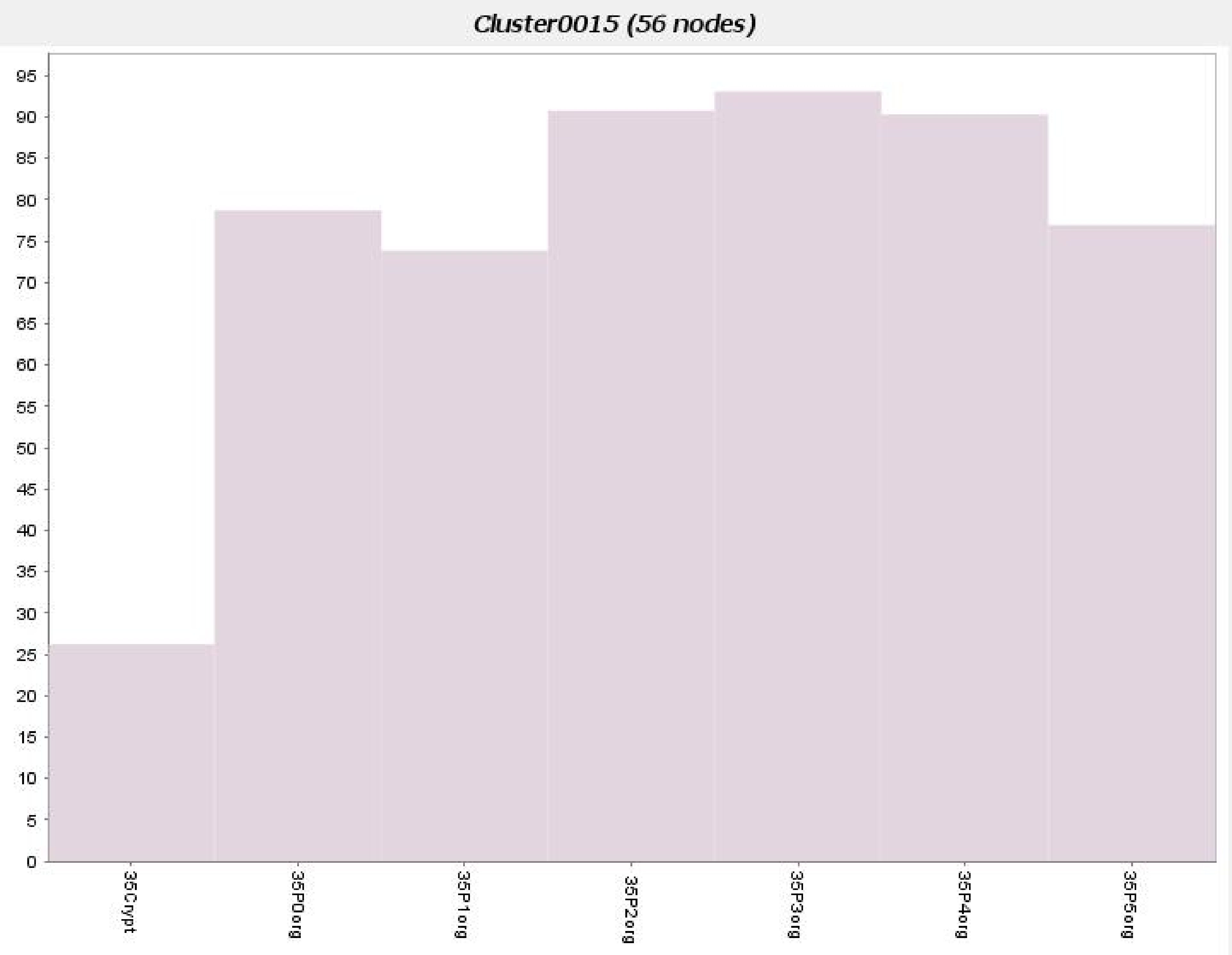

Cluster0015 (56 nodes)
95
90
85
80
75
70
65
60
55
50
45
40
35
30
25
20
15
10
5
0
35Crypt
35P0org
35P1org
35P2org
35P3org
35P4org
35P5org

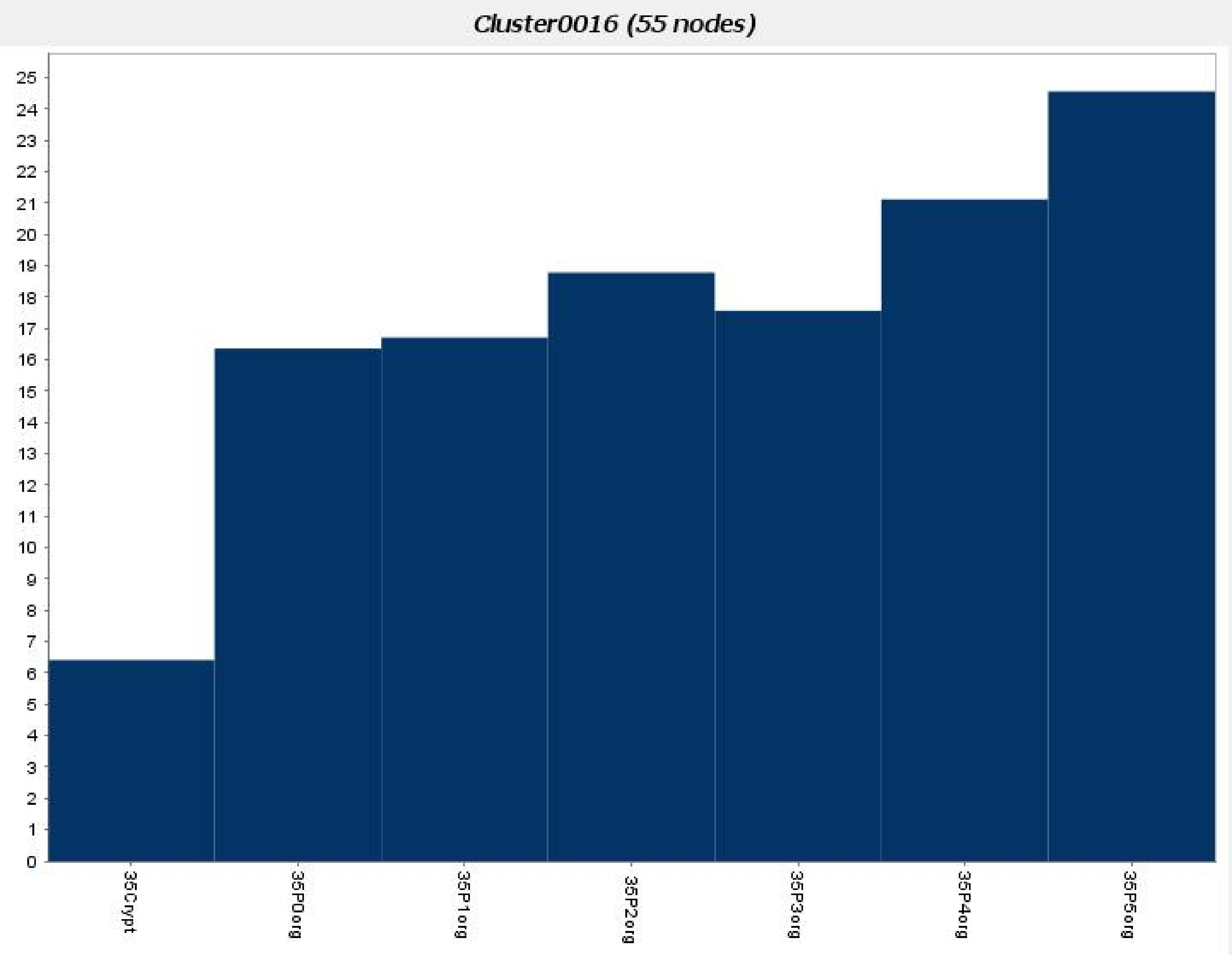
Cluster0016 (55 nodes)
25
24
23
22
21
20
19
18
17
16
15
14
13
12
11
10
9
8
7
6
5
4
3
2
1
0
35Crypt
35P0org
35P1org
35P2org
35P3org
35P4org
35P5org

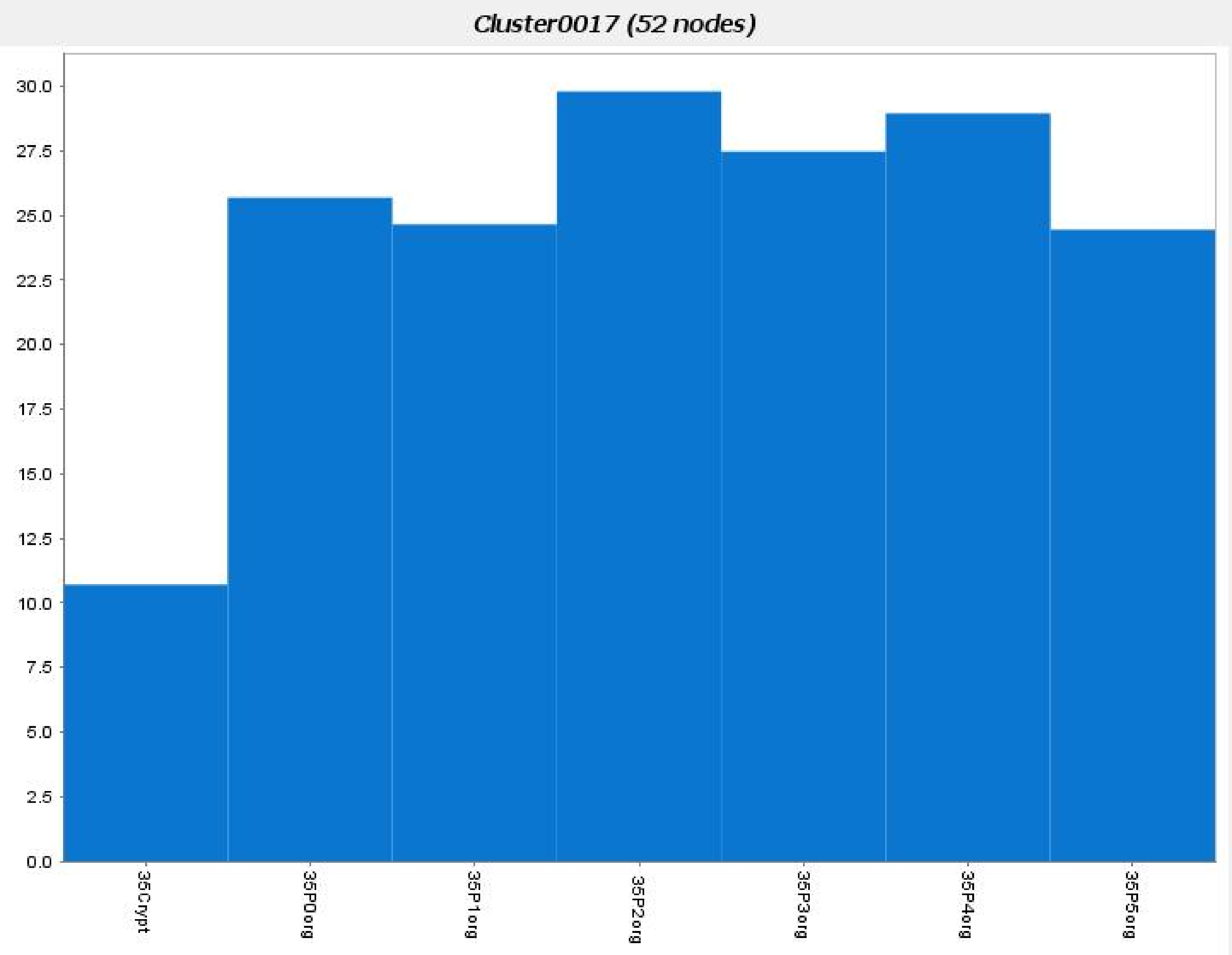

Cluster0017 (52 nodes)
30.0
27.5
25.0
22.5
20.0
17.5
15.0
12.5
10.0
7.5
5.0
2.5
0.0
35Crypt
35P0org
35P1org
35P2org
35P3org
35P4org
35P5org

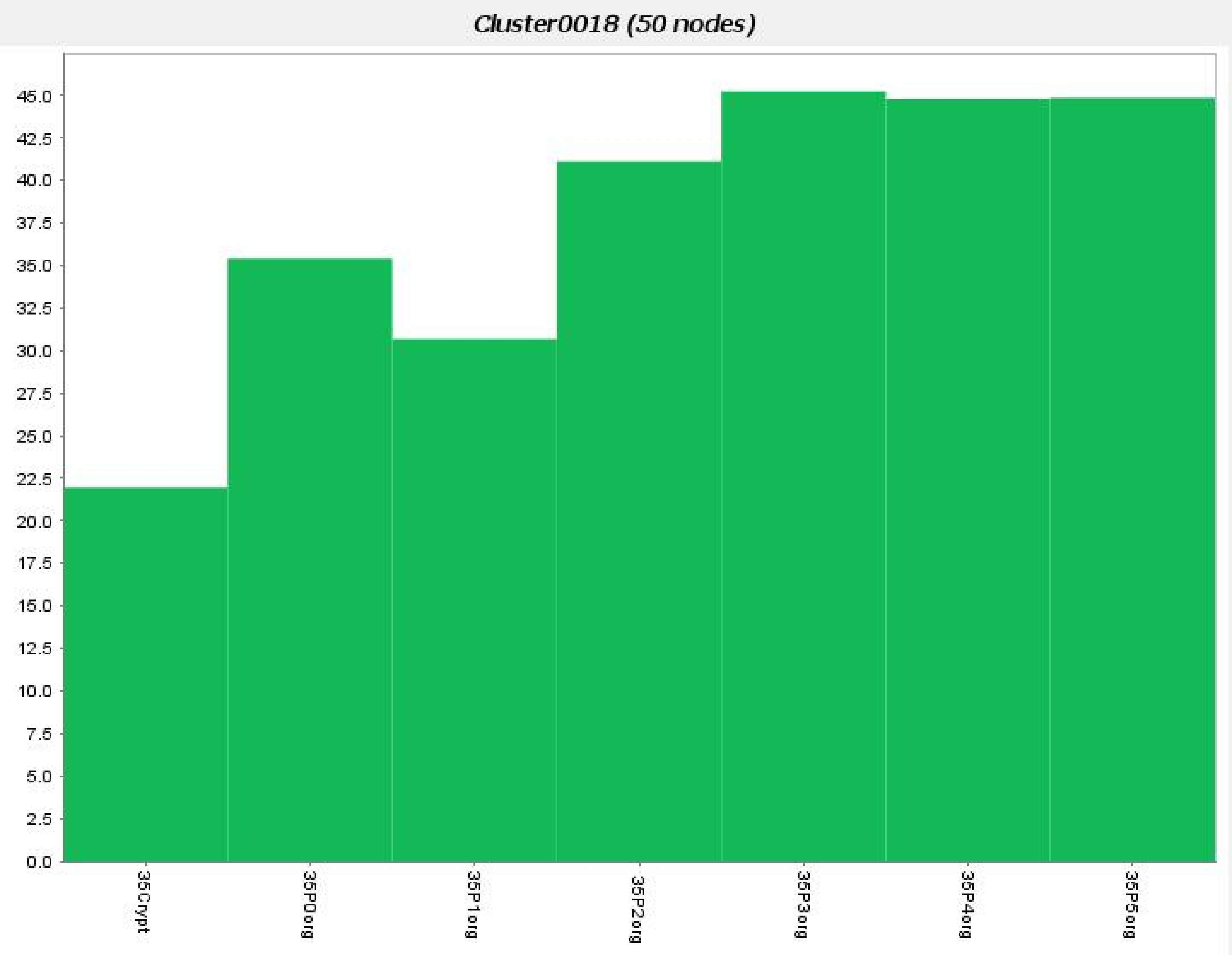
Cluster0018 (50 nodes)
45.0
42.5
40.0
37.5
35.0
32.5
30.0
27.5
25.0
22.5
20.0
17.5
15.0
12.5
10.0
7.5
5.0
2.5
0.0
35Crypt
35P0org
35P1org
35P2org
35P3org
35P4org
35P5org

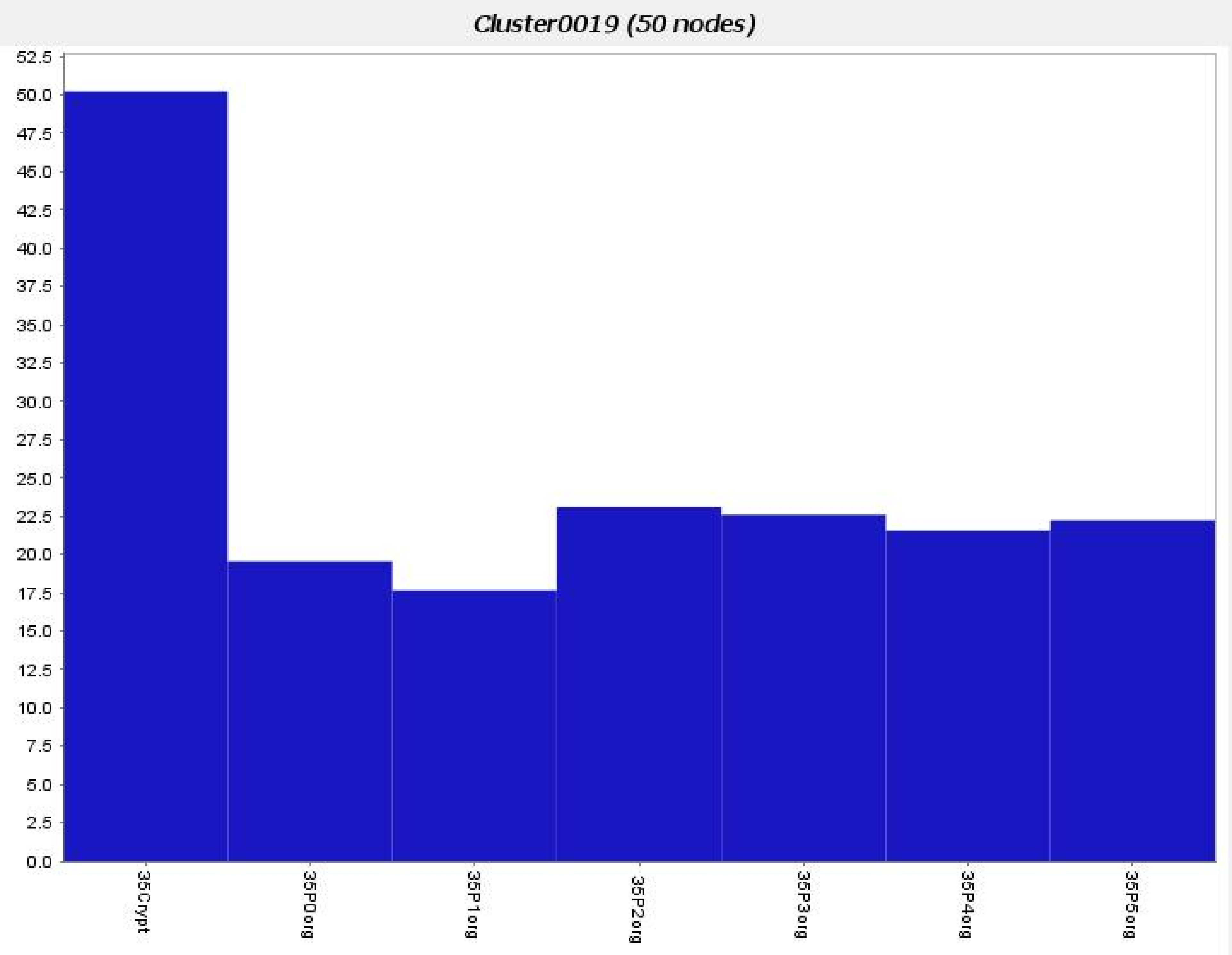

Cluster0019 (50 nodes)
52.5
50.0
47.5
45.0
42.5
40.0
37.5
35.0
32.5
30.0
27.5
25.0
22.5
20.0
17.5
15.0
12.5
10.0
7.5
5.0
2.5
0.0
35Crypt
35P0org
35P1org
35P2org
35P3org
35P4org
35P5org

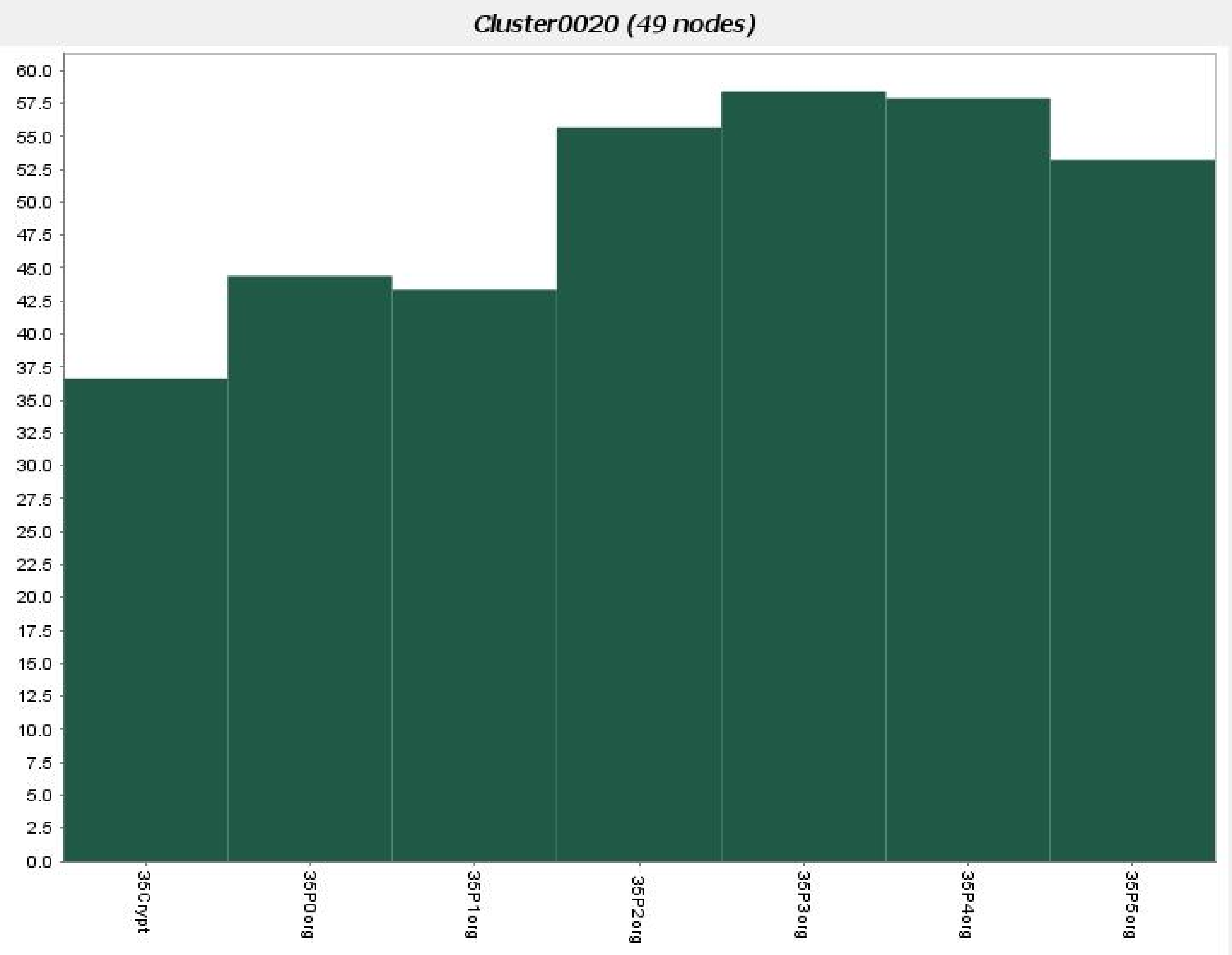
Cluster0020 (49 nodes)
60.0
57.5
55.0
52.5
50.0
47.5
45.0
42.5
40.0
37.5
35.0
32.5
30.0
27.5
25.0
22.5
20.0
17.5
15.0
12.5
10.0
7.5
5.0
2.5
0.0
35Crypt
35P0org
35P1org
35P2org
35P3org
35P4org
35P5org

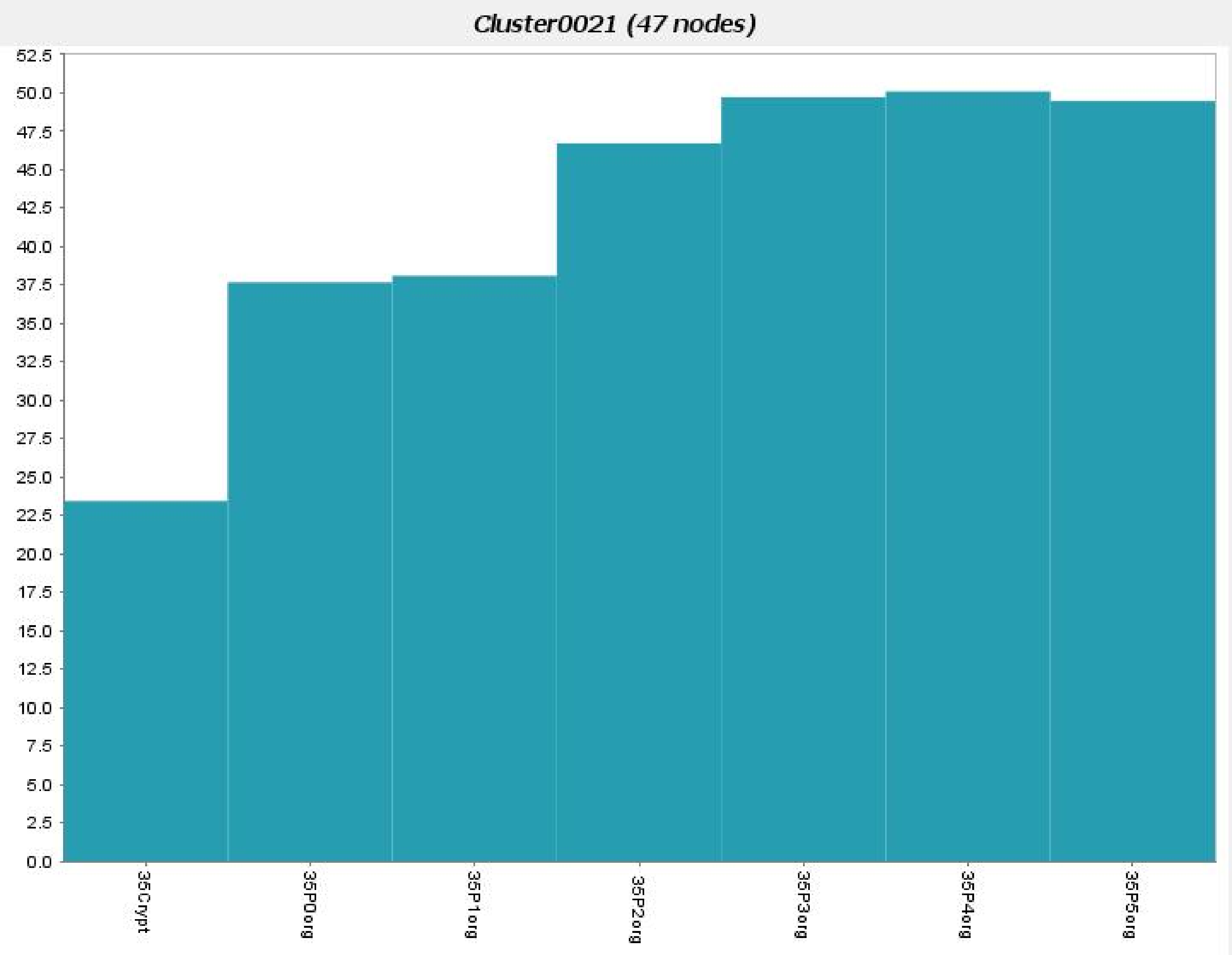
Cluster0021 (47 nodes)
52.5
50.0
47.5
45.0
42.5
40.0
37.5
35.0
32.5
30.0
27.5
25.0
22.5
20.0
17.5
15.0
12.5
10.0
7.5
5.0
2.5
0.0
35Crypt
35P0org
35P1org
35P2org
35P3org
35P4org
35P5org

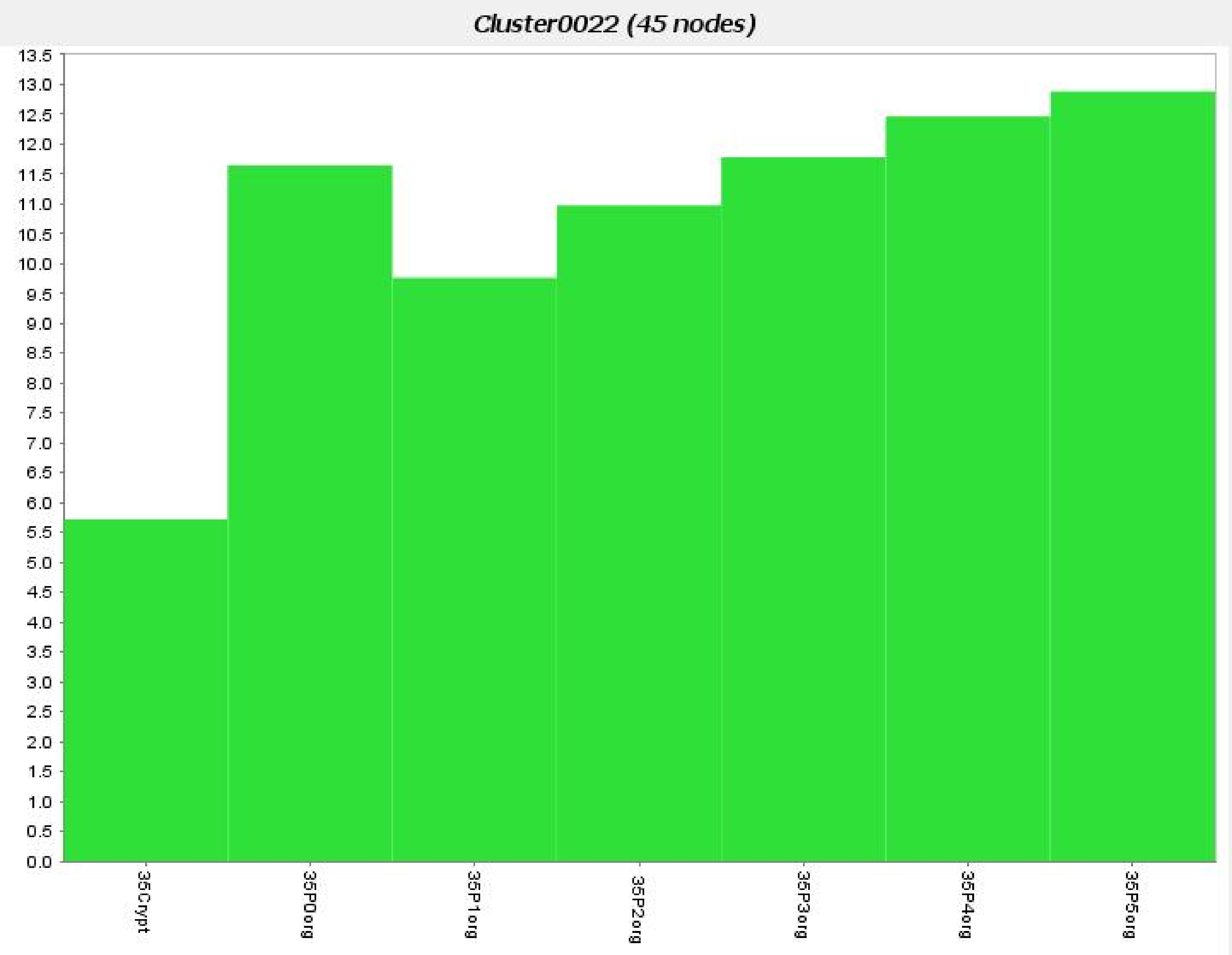

Cluster0022 (45 nodes)
13.5
13.0
12.5
12.0
11.5
11.0
10.5
10.0
9.5
9.0
8.5
8.0
7.5
7.0
6.5
6.0
5.5
5.0
4.5
4.0
3.5
3.0
2.5
2.0
1.5
1.0
0.5
0.0
35Crypt
35P0org
35P1org
35P2org
35P3org
35P4org
35P5org

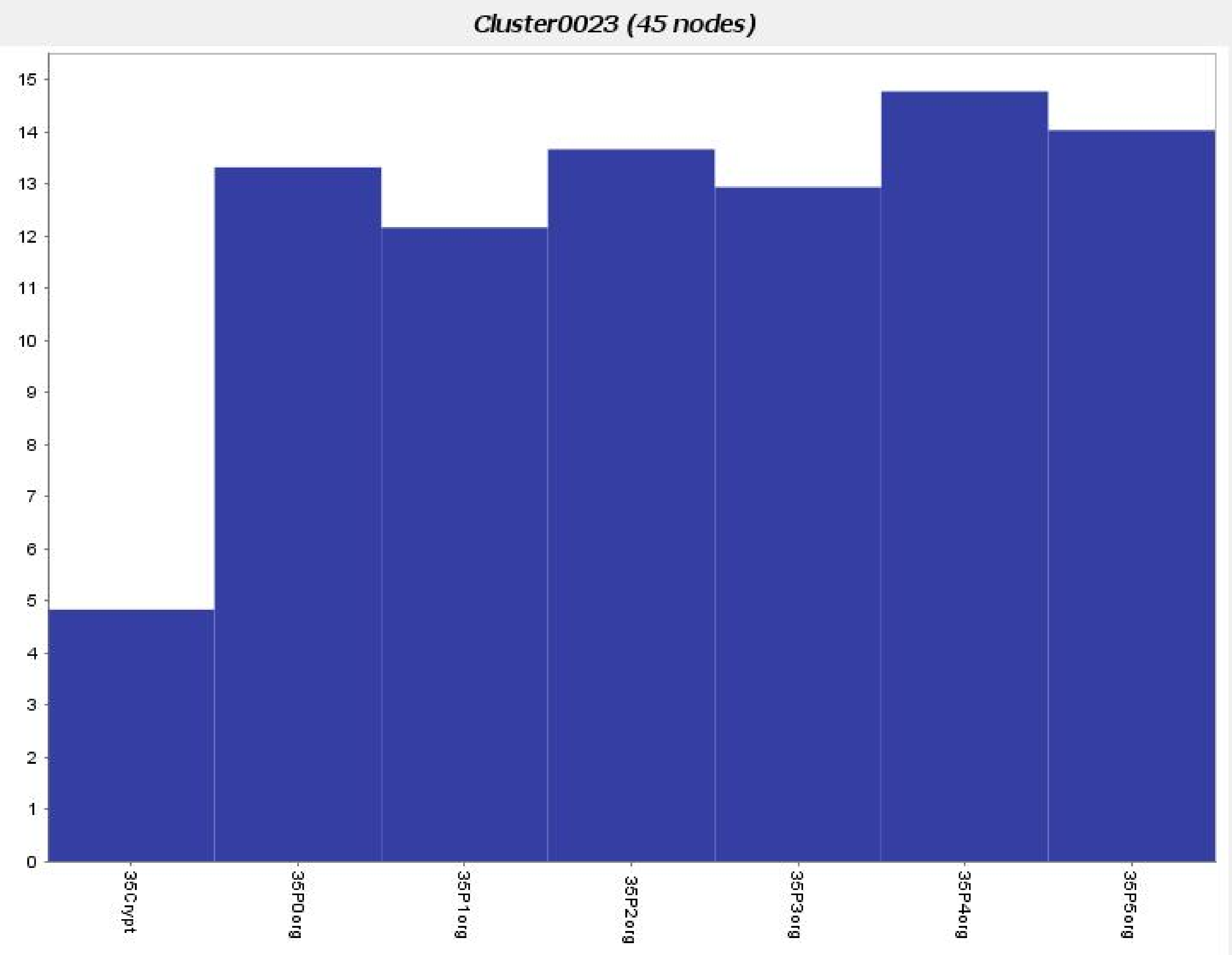
Cluster0023 (45 nodes)
0
1
2
3
4
5
6
7
8
9
10
11
12
13
14
15
35Crypt
35P0org
35P1org
35P2org
35P3org
35P4org
35P5org

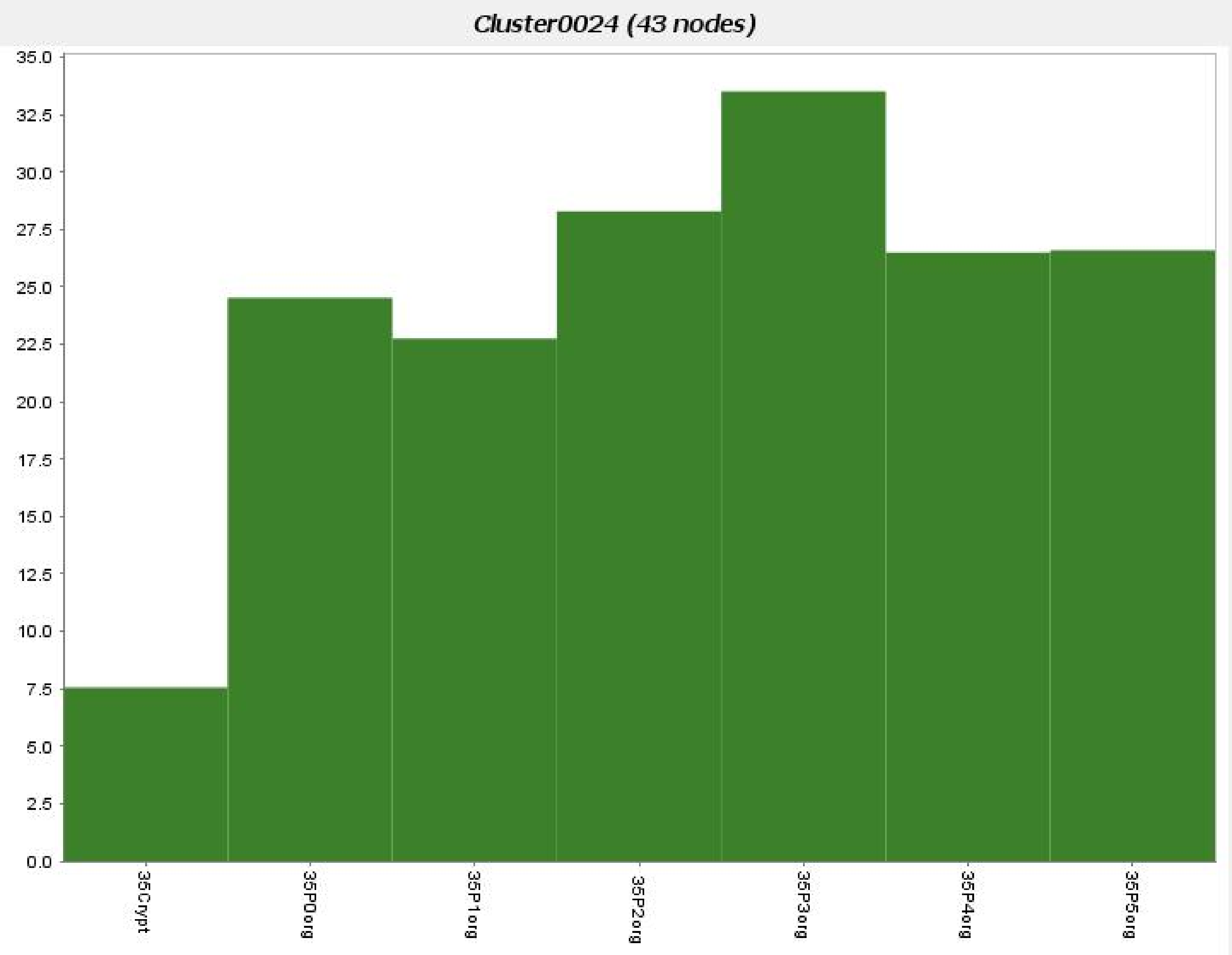
Cluster0024 (43 nodes)
35.0
32.5
30.0
27.5
25.0
22.5
20.0
17.5
15.0
12.5
10.0
7.5
5.0
2.5
0.0
35Crypt
35P0org
35P1org
35P2org
35P3org
35P4org
35P5org

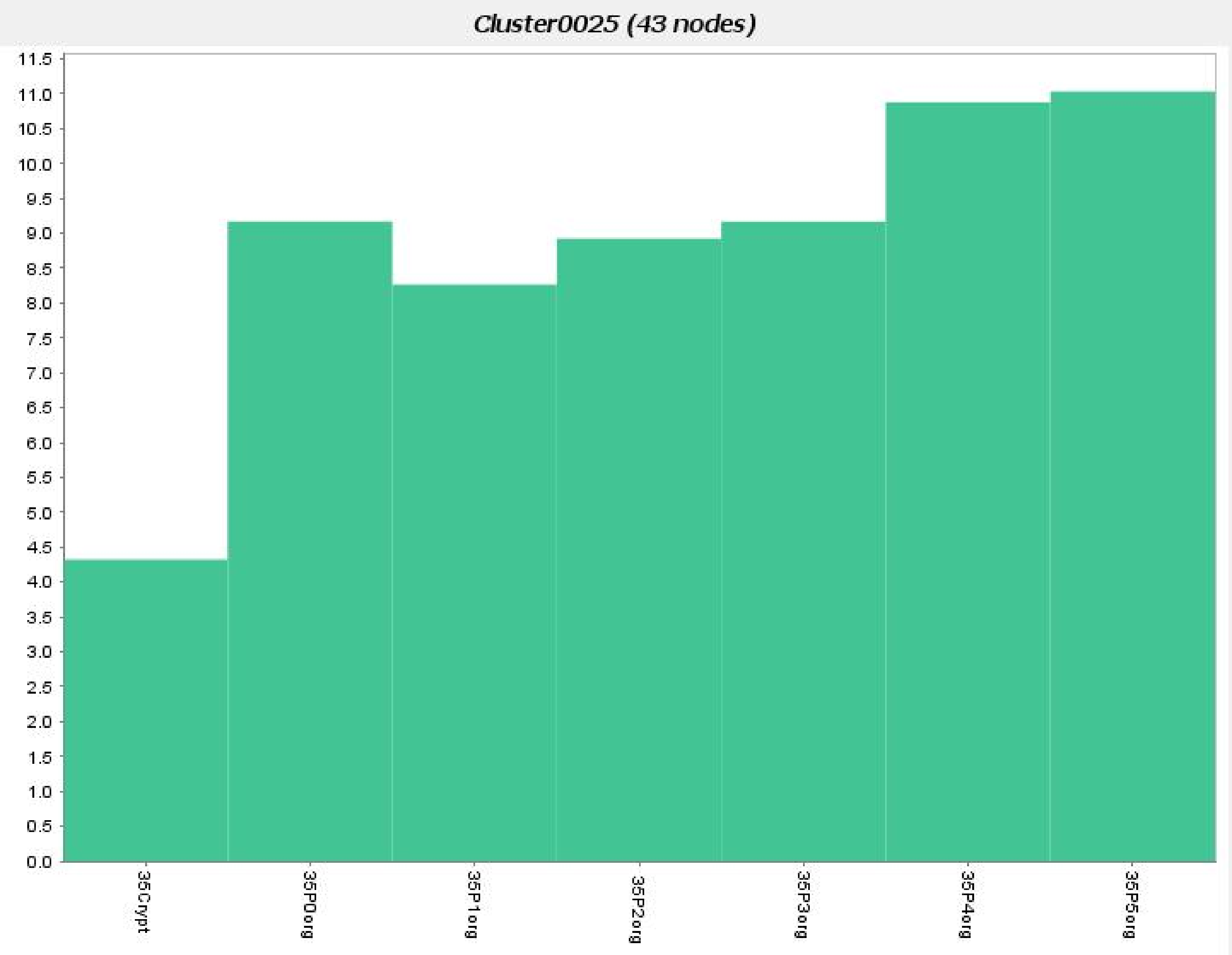
Cluster0025 (43 nodes)
11.5
11.0
10.5
10.0
9.5
9.0
8.5
8.0
7.5
7.0
6.5
6.0
5.5
5.0
4.5
4.0
3.5
3.0
2.5
2.0
1.5
1.0
0.5
0.0
35Crypt
35P0org
35P1org
35P2org
35P3org
35P4org
35P5org

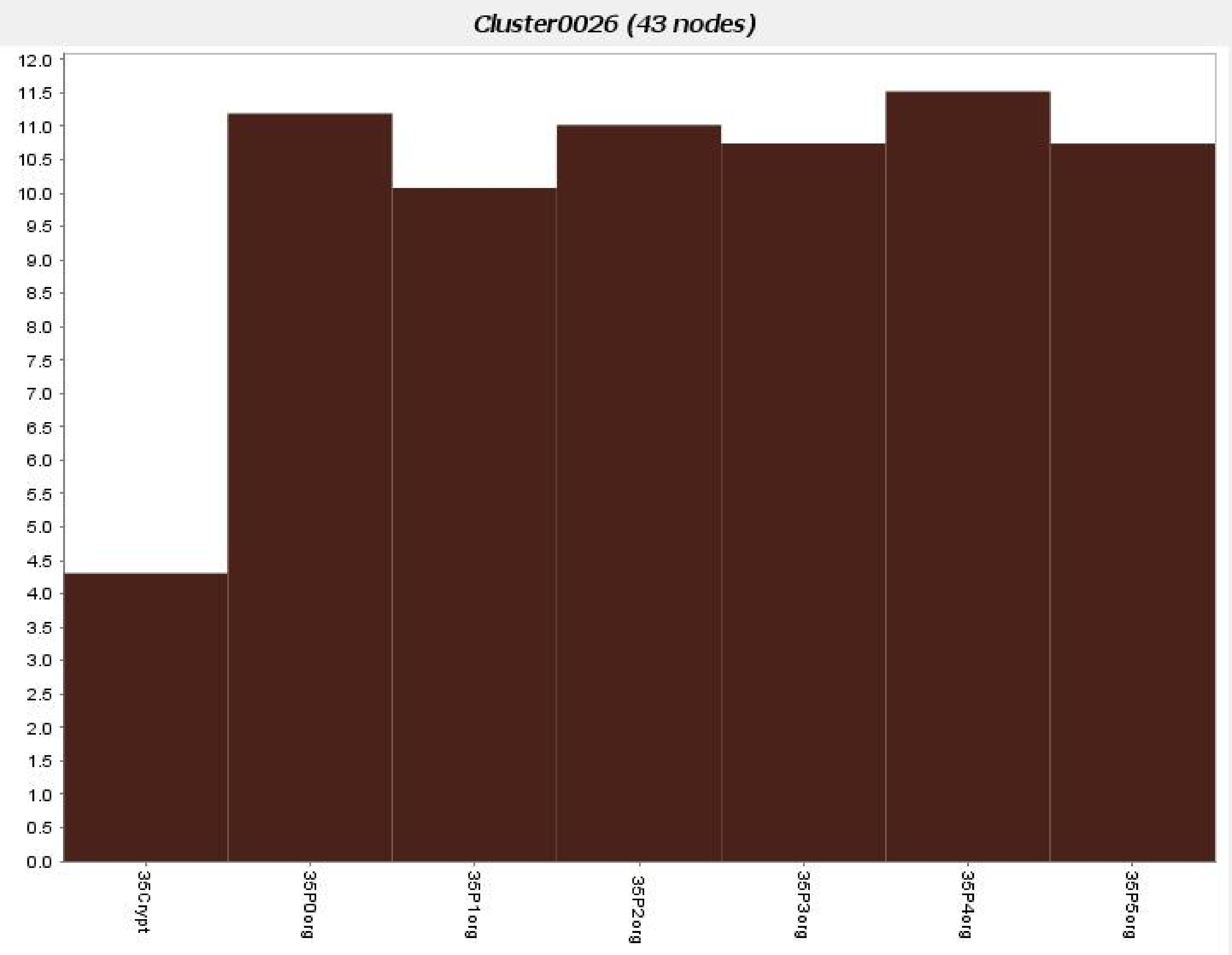

Cluster0026 (43 nodes)
12.0
11.5
11.0
10.5
10.0
9.5
9.0
8.5
8.0
7.5
7.0
6.5
6.0
5.5
5.0
4.5
4.0
3.5
3.0
2.5
2.0
1.5
1.0
0.5
0.0
35Crypt
35P0org
35P1org
35P2org
35P3org
35P4org
35P5org

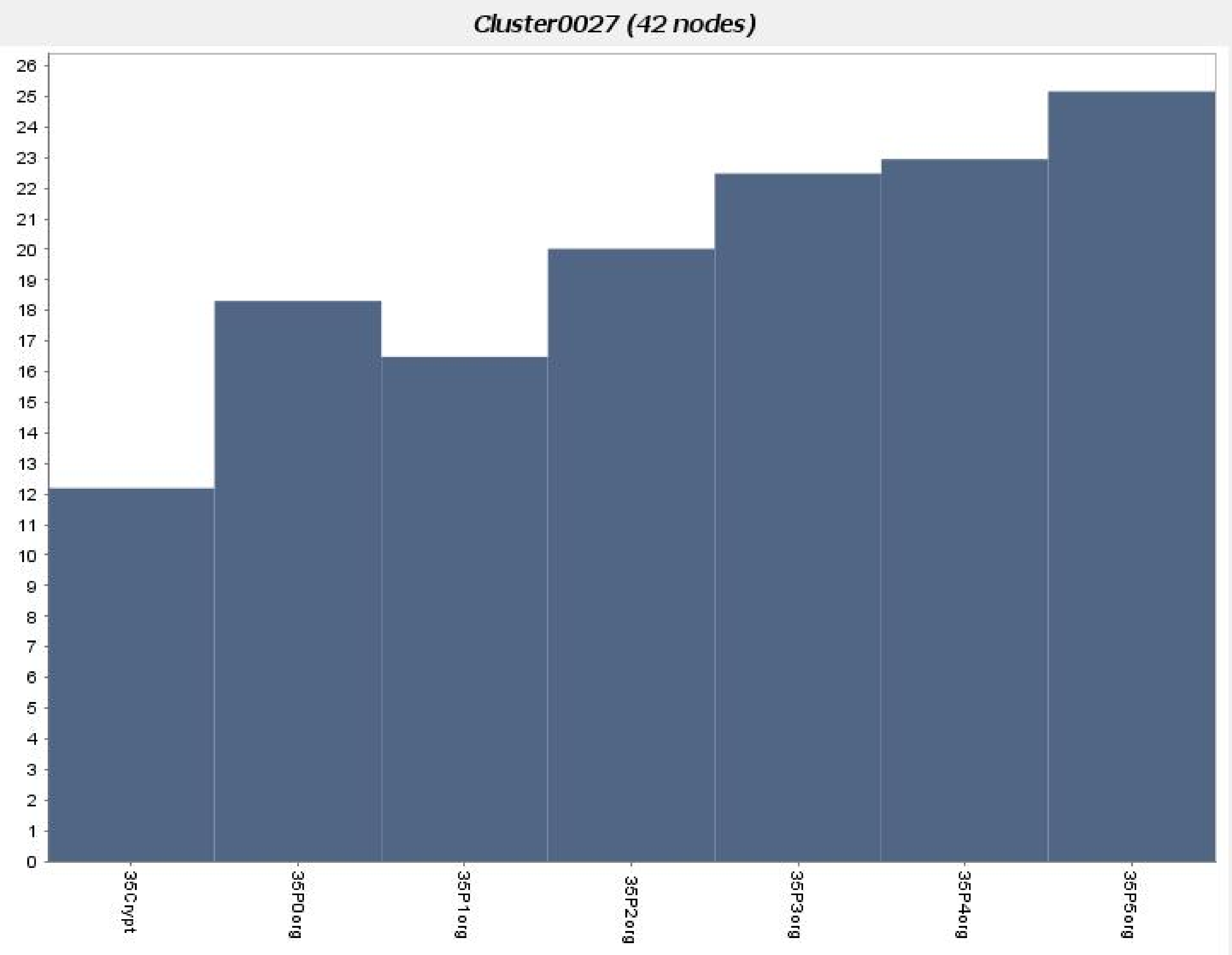

Cluster0027 (42 nodes)
0
1
2
3
4
5
6
7
8
9
10
11
12
13
14
15
16
17
18
19
20
21
22
23
24
25
26
35Crypt
35P0org
35P1org
35P2org
35P3org
35P4org
35P5org

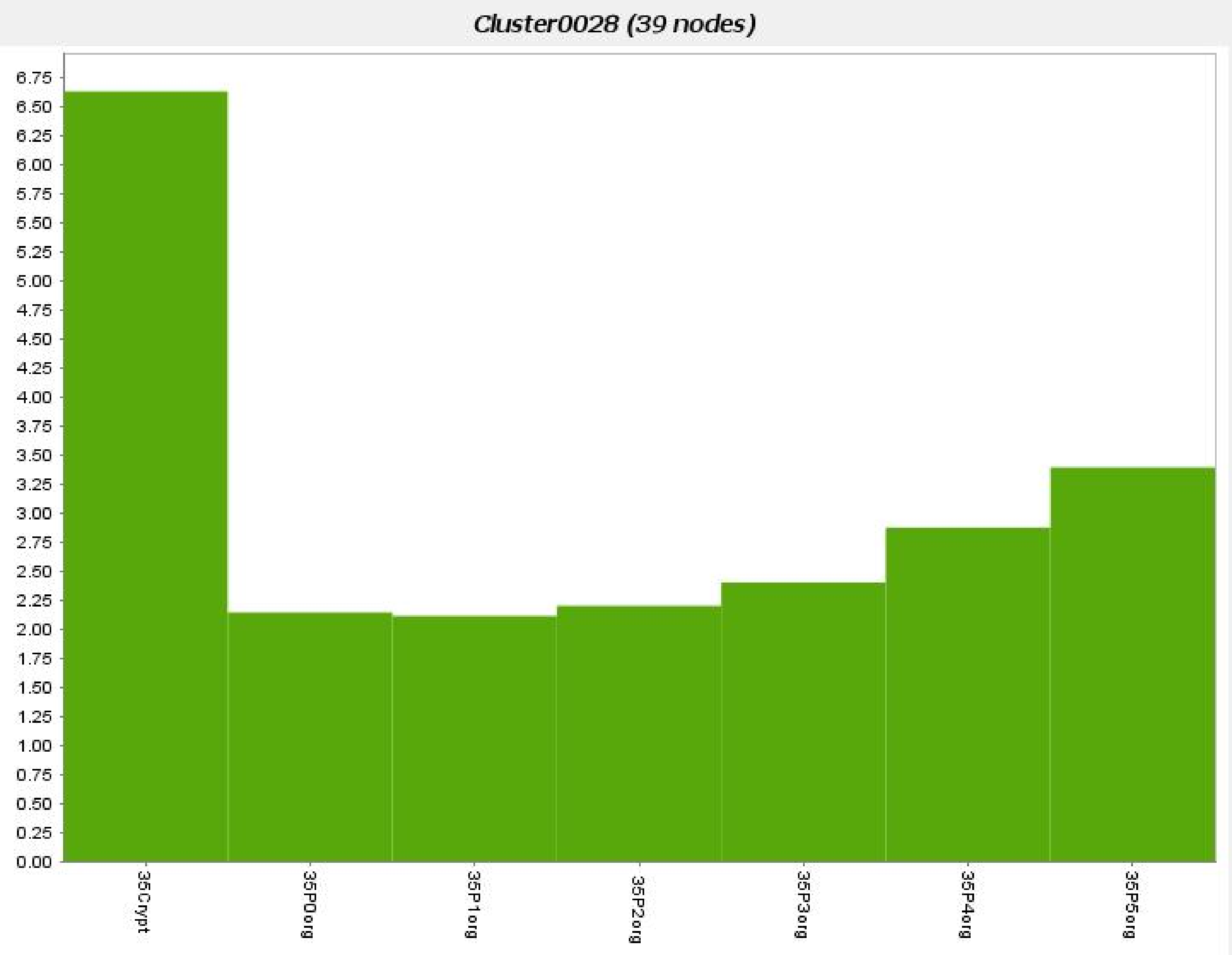

Cluster0028 (39 nodes)
6.75
6.50
6.25
6.00
5.75
5.50
5.25
5.00
4.75
4.50
4.25
4.00
3.75
3.50
3.25
3.00
2.75
2.50
2.25
2.00
1.75
1.50
1.25
1.00
0.75
0.50
0.25
0.00
35Crypt
35P0org
35P1org
35P2org
35P3org
35P4org
35P5org

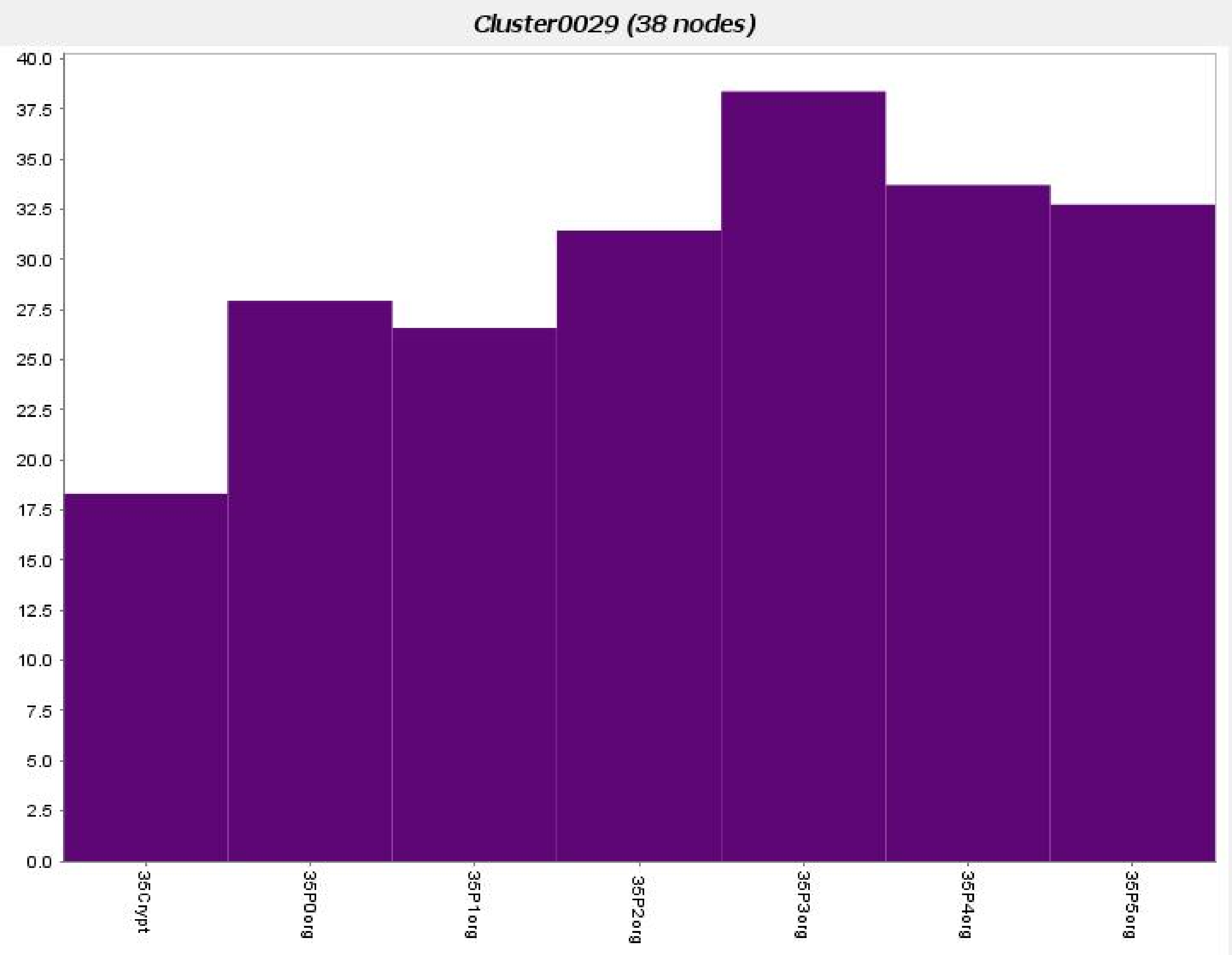

Cluster0029 (38 nodes)
40.0
37.5
35.0
32.5
30.0
27.5
25.0
22.5
20.0
17.5
15.0
12.5
10.0
7.5
5.0
2.5
0.0
35Crypt
35P0org
35P1org
35P2org
35P3org
35P4org
35P5org

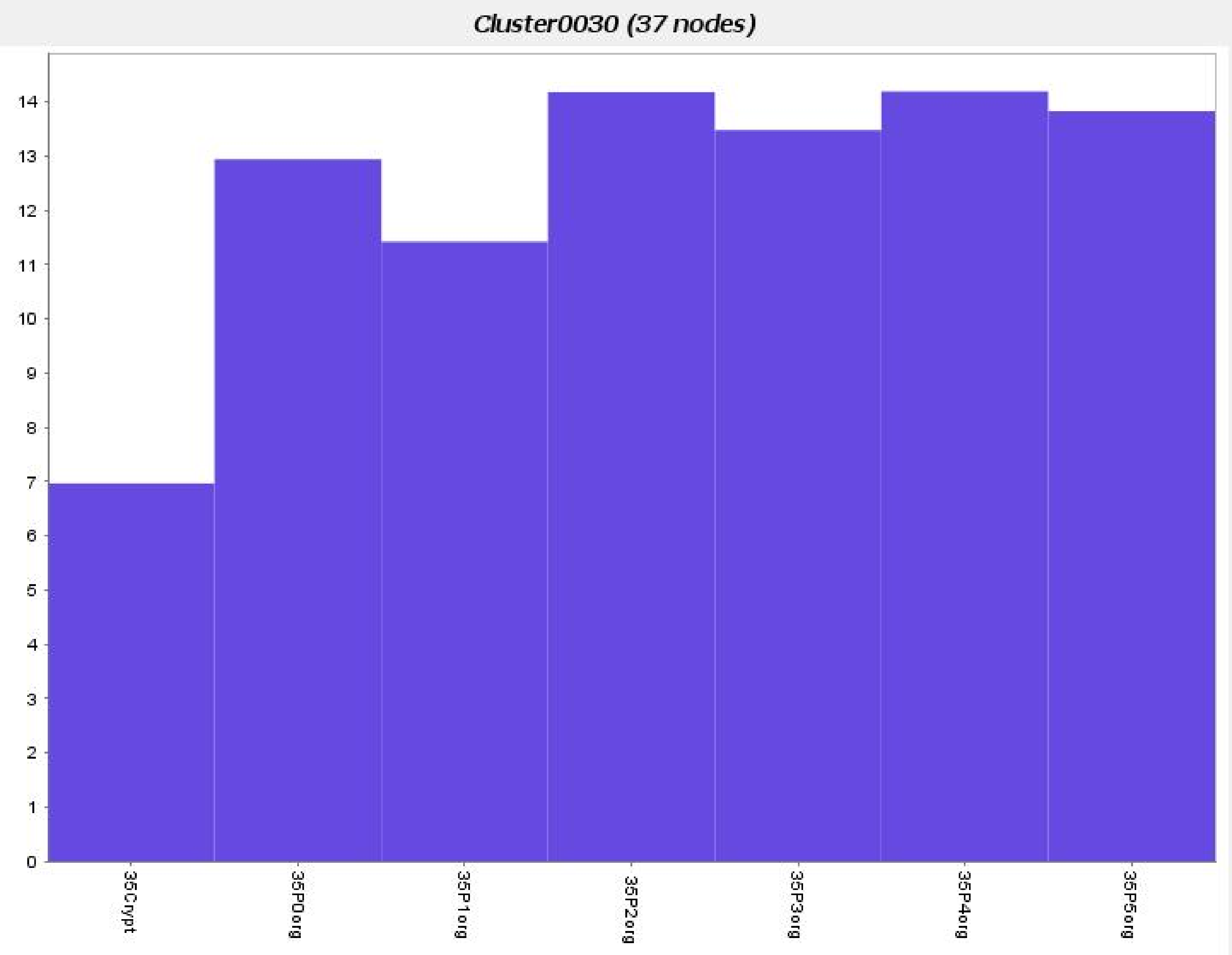

Cluster0030 (37 nodes)
14
13
12
11
10
9
8
7
6
5
4
3
2
1
0
35Crypt
35P0org
35P1org
35P2org
35P3org
35P4org
35P5org

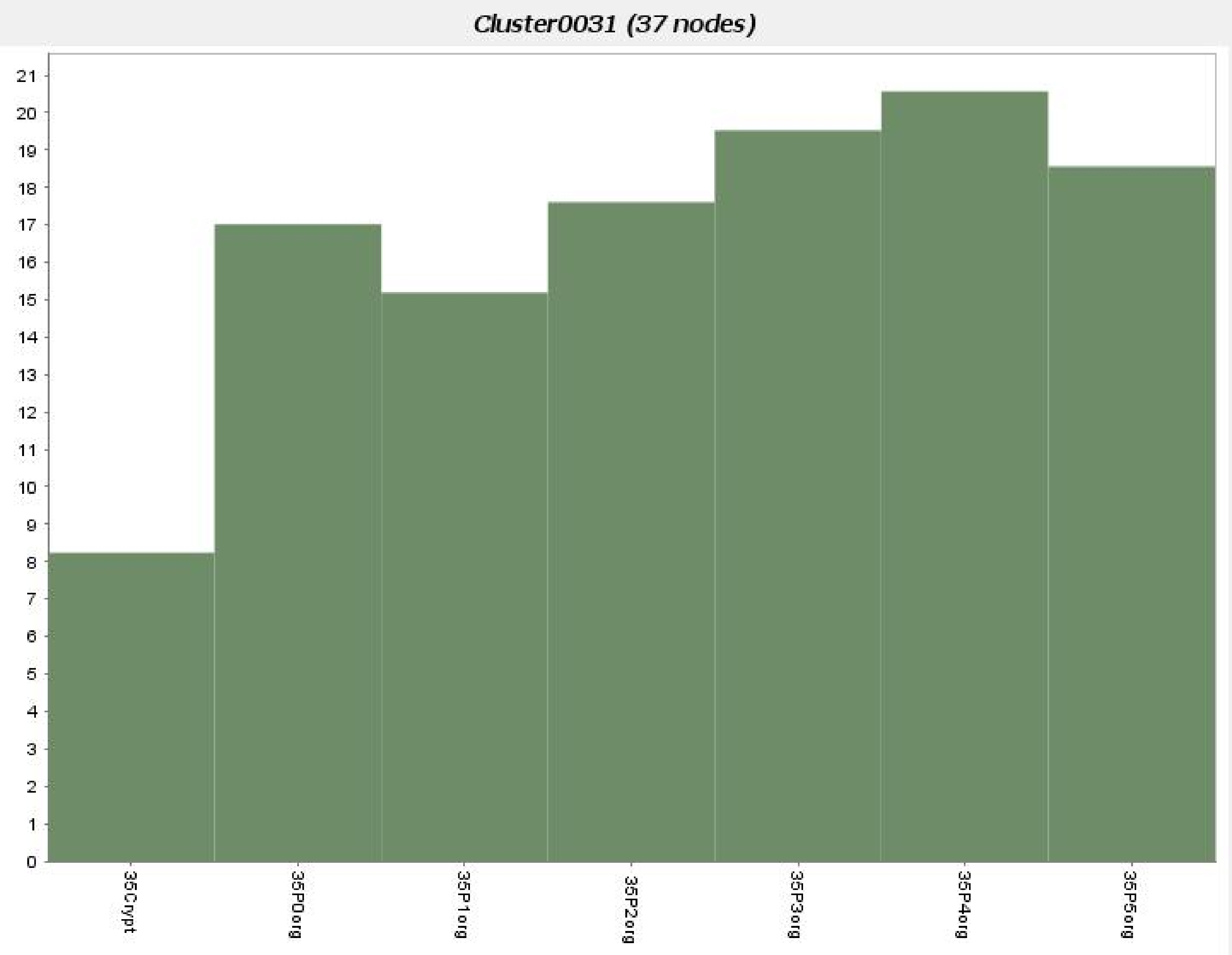
Cluster0031 (37 nodes)
21
20
19
18
17
16
15
14
13
12
11
10
9
8
7
6
5
4
3
2
1
0
35Crypt
35P0org
35P1org
35P2org
35P3org
35P4org
35P5org

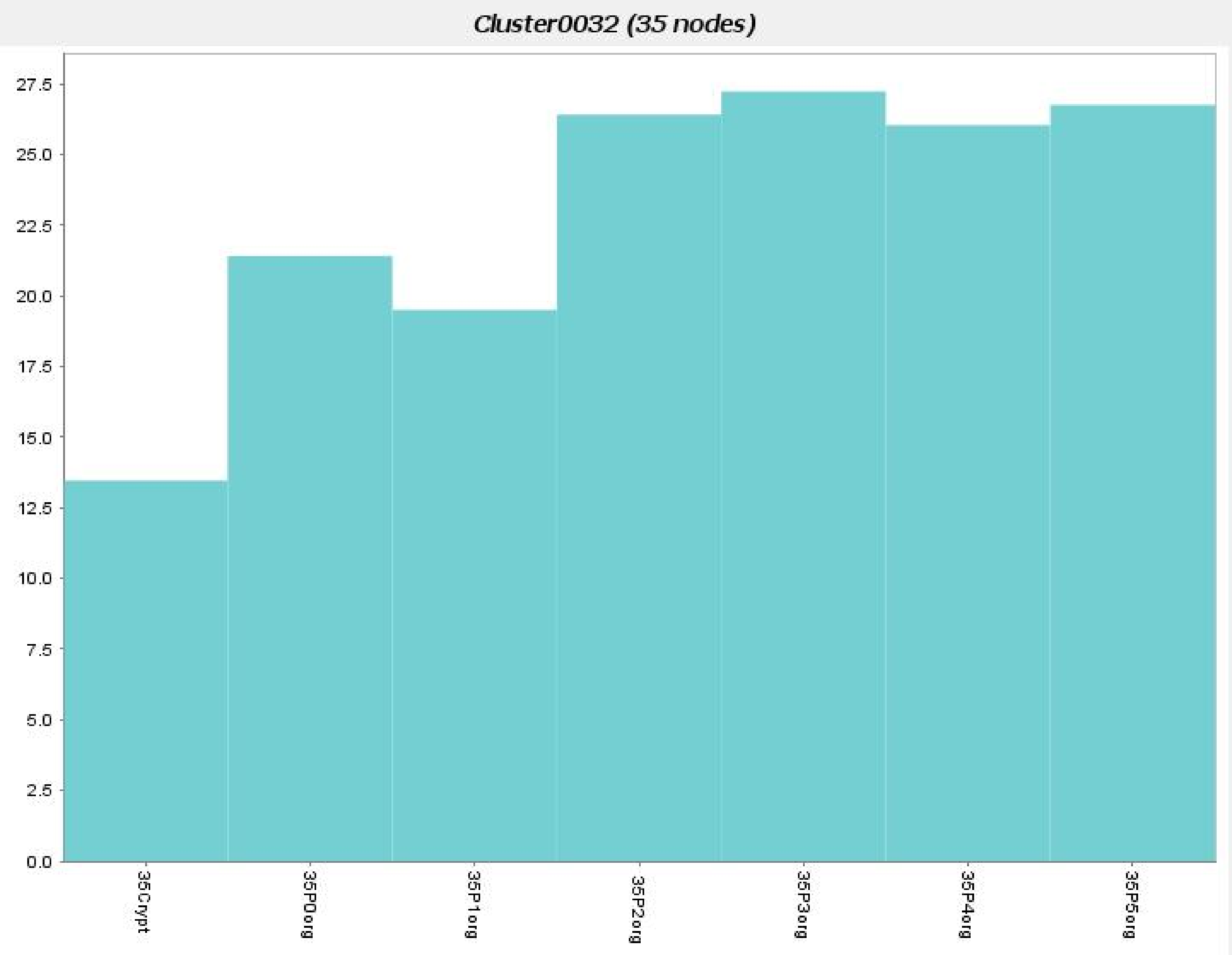

Cluster0032 (35 nodes)
27.5
25.0
22.5
20.0
17.5
15.0
12.5
10.0
7.5
5.0
2.5
0.0
35Crypt
35P0org
35P1org
35P2org
35P3org
35P4org
35P5org

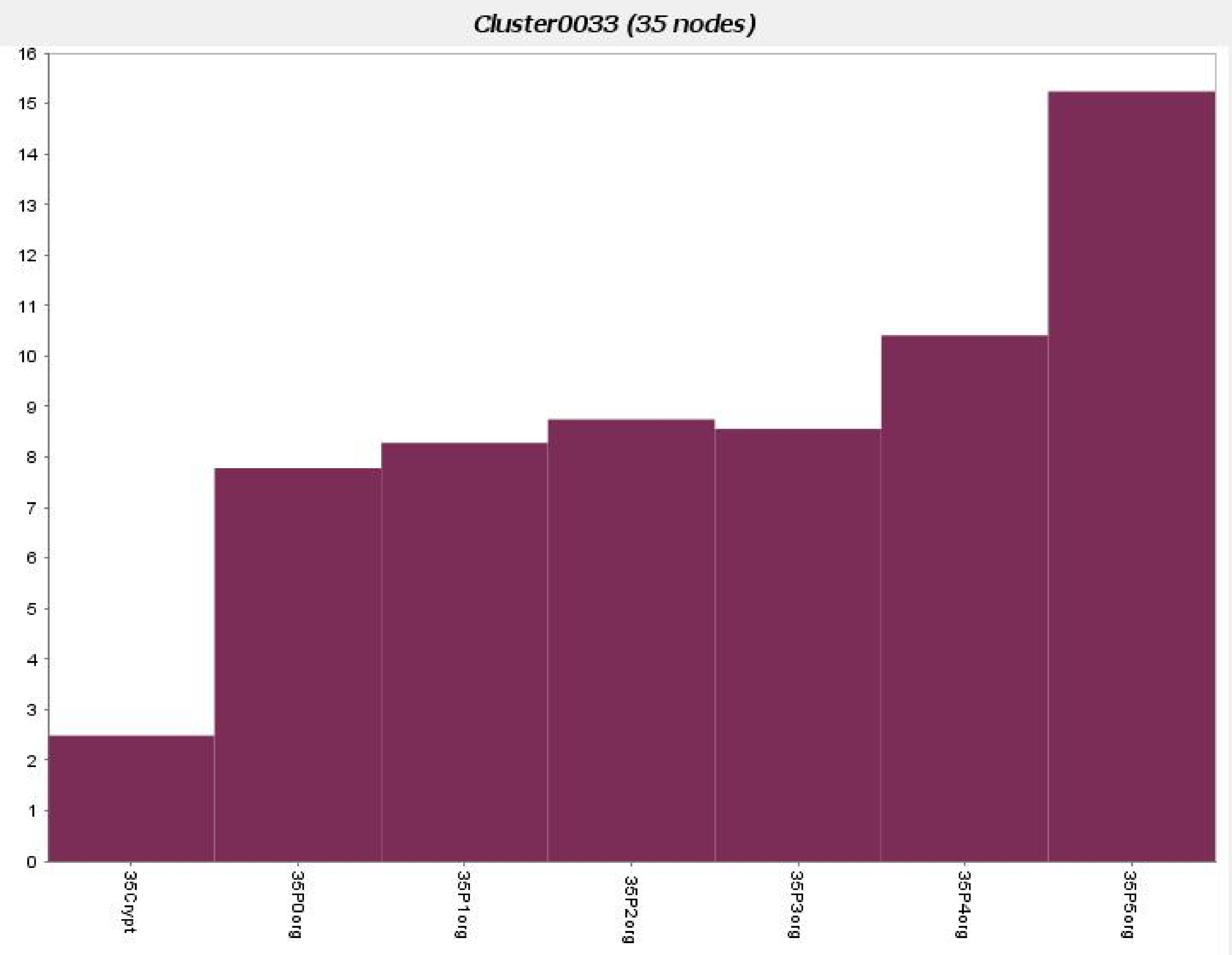

Cluster0033 (35 nodes)
16
15
14
13
12
11
10
9
8
7
6
5
4
3
2
1
0
35Crypt
35P0org
35P1org
35P2org
35P3org
35P4org
35P5org

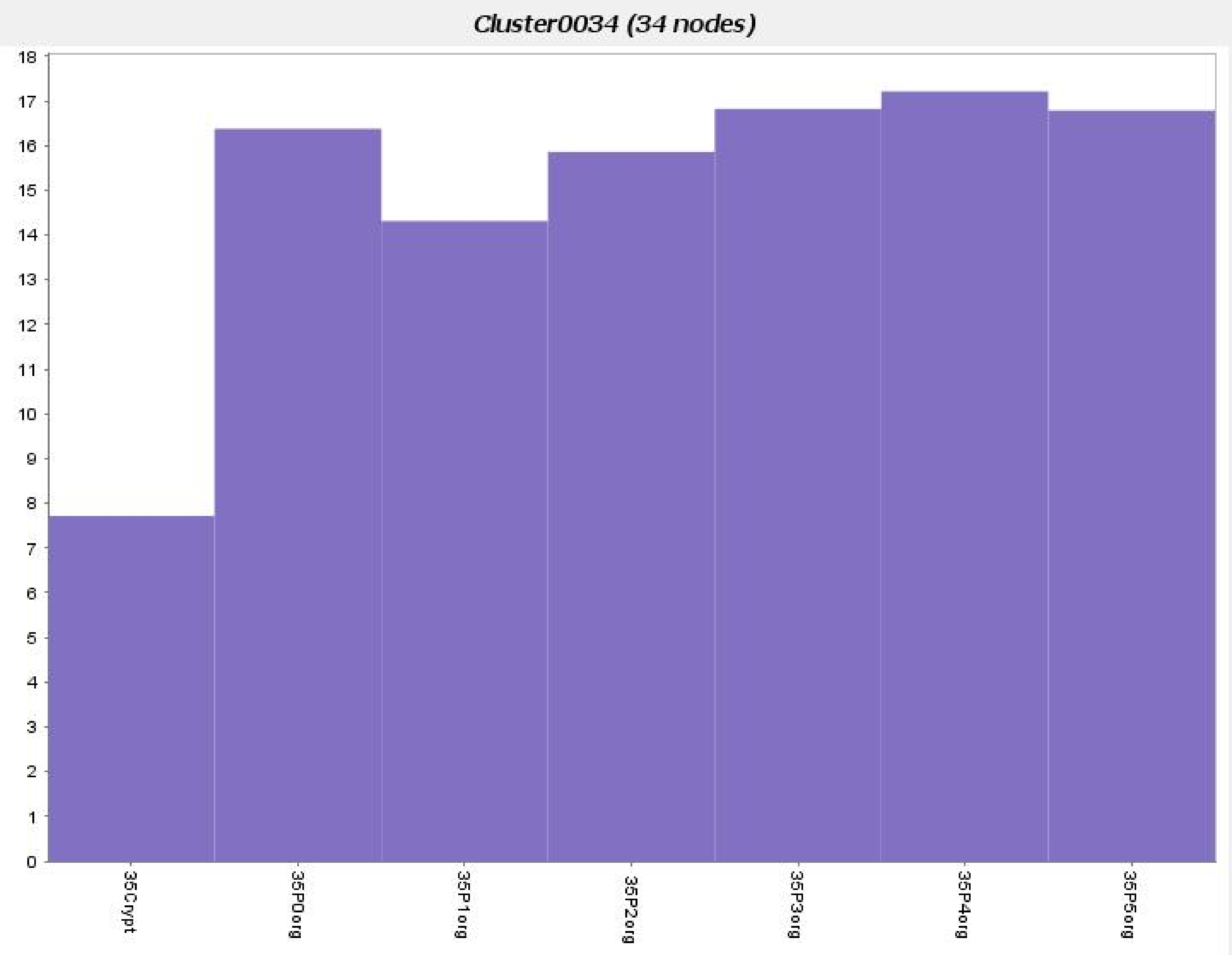

Cluster0034 (34 nodes)
18
17
16
15
14
13
12
11
10
9
8
7
6
5
4
3
2
1
0
35Crypt
35P0org
35P1org
35P2org
35P3org
35P4org
35P5org

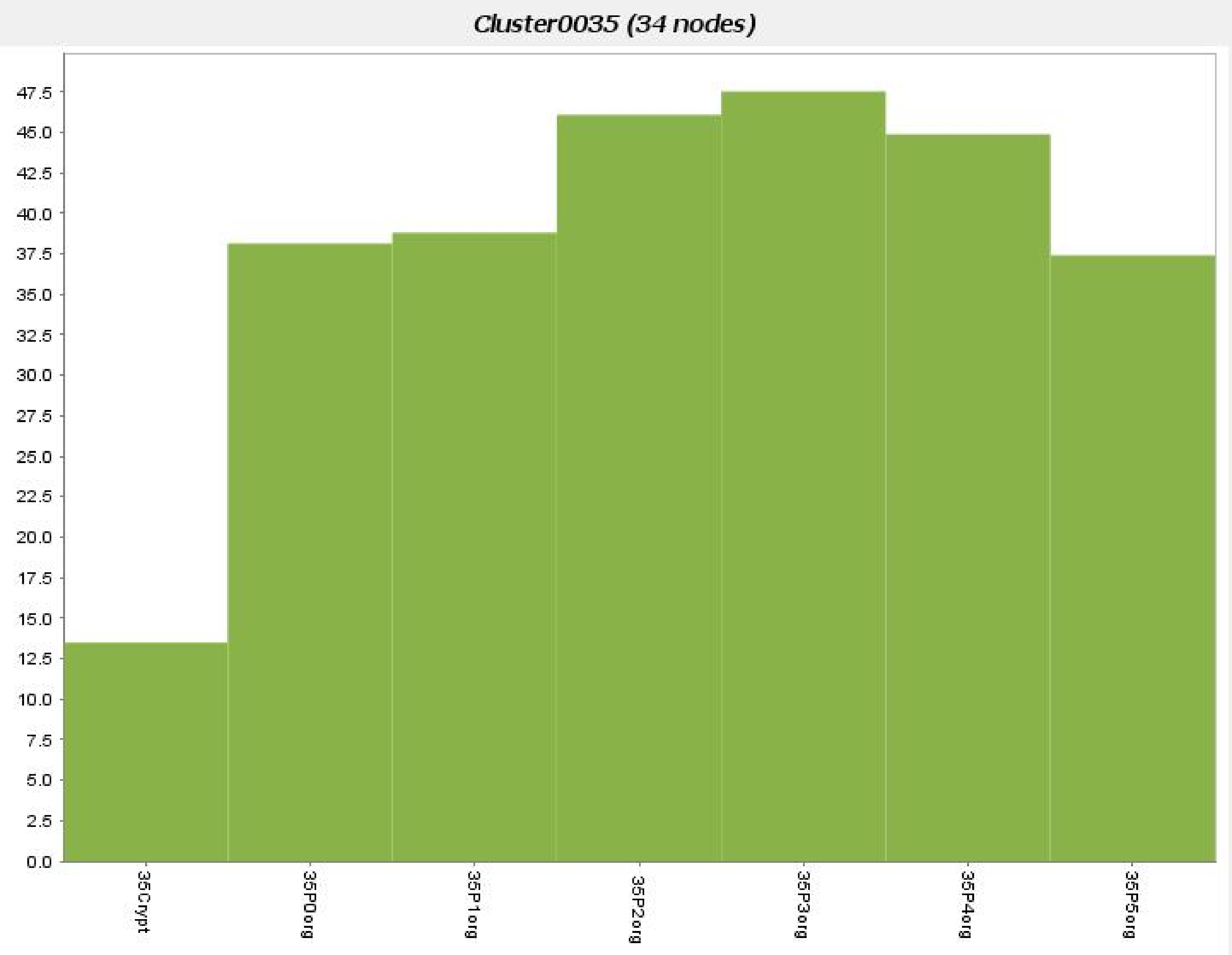

Cluster0035 (34 nodes)
47.5
45.0
42.5
40.0
37.5
35.0
32.5
30.0
27.5
25.0
22.5
20.0
17.5
15.0
12.5
10.0
7.5
5.0
2.5
0.0
35Crypt
35P0org
35P1org
35P2org
35P3org
35P4org
35P5org

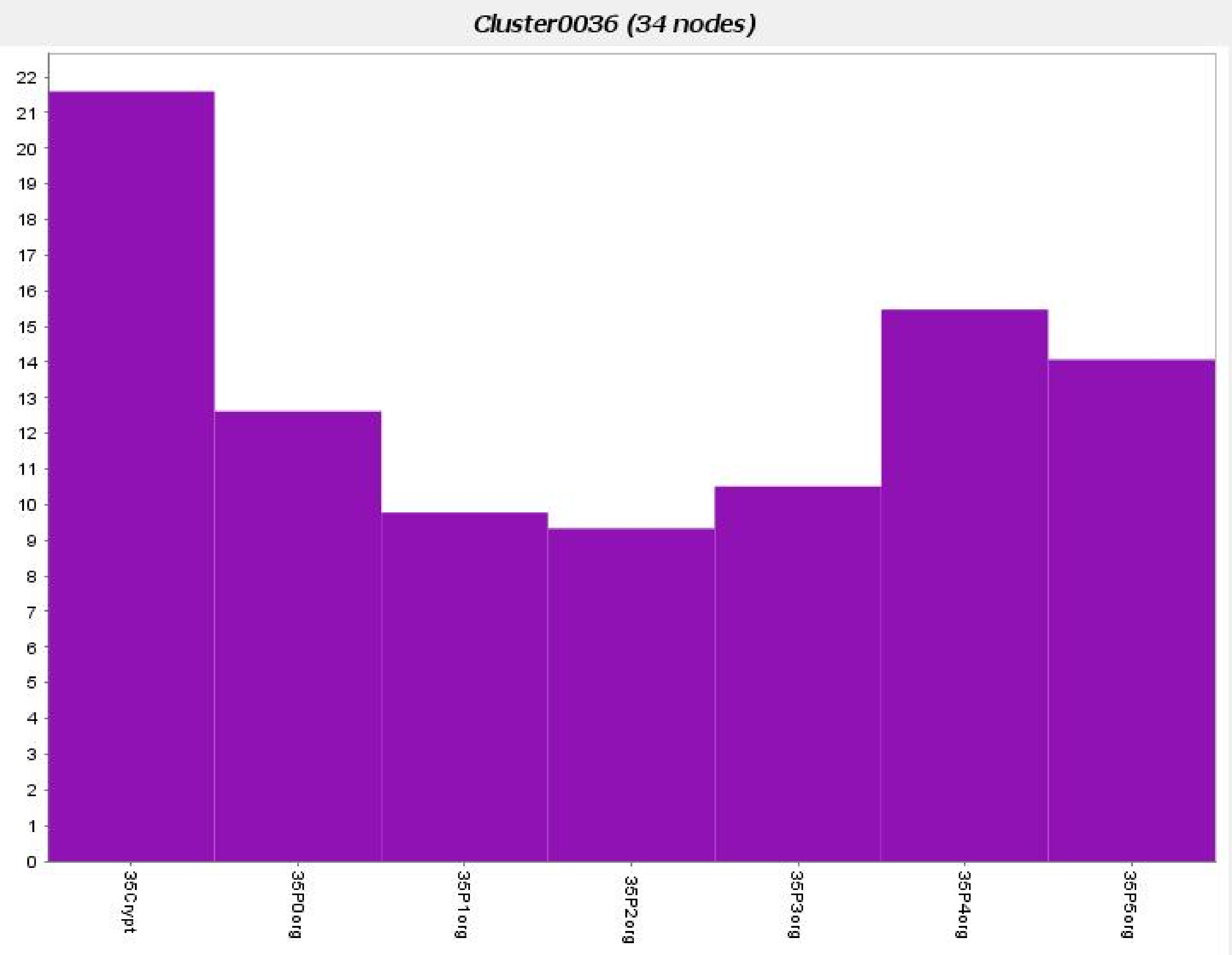

Cluster0036 (34 nodes)
22
21
20
19
18
17
16
15
14
13
12
11
10
9
8
7
6
5
4
3
2
1
0
35Crypt
35P0org
35P1org
35P2org
35P3org
35P4org
35P5org

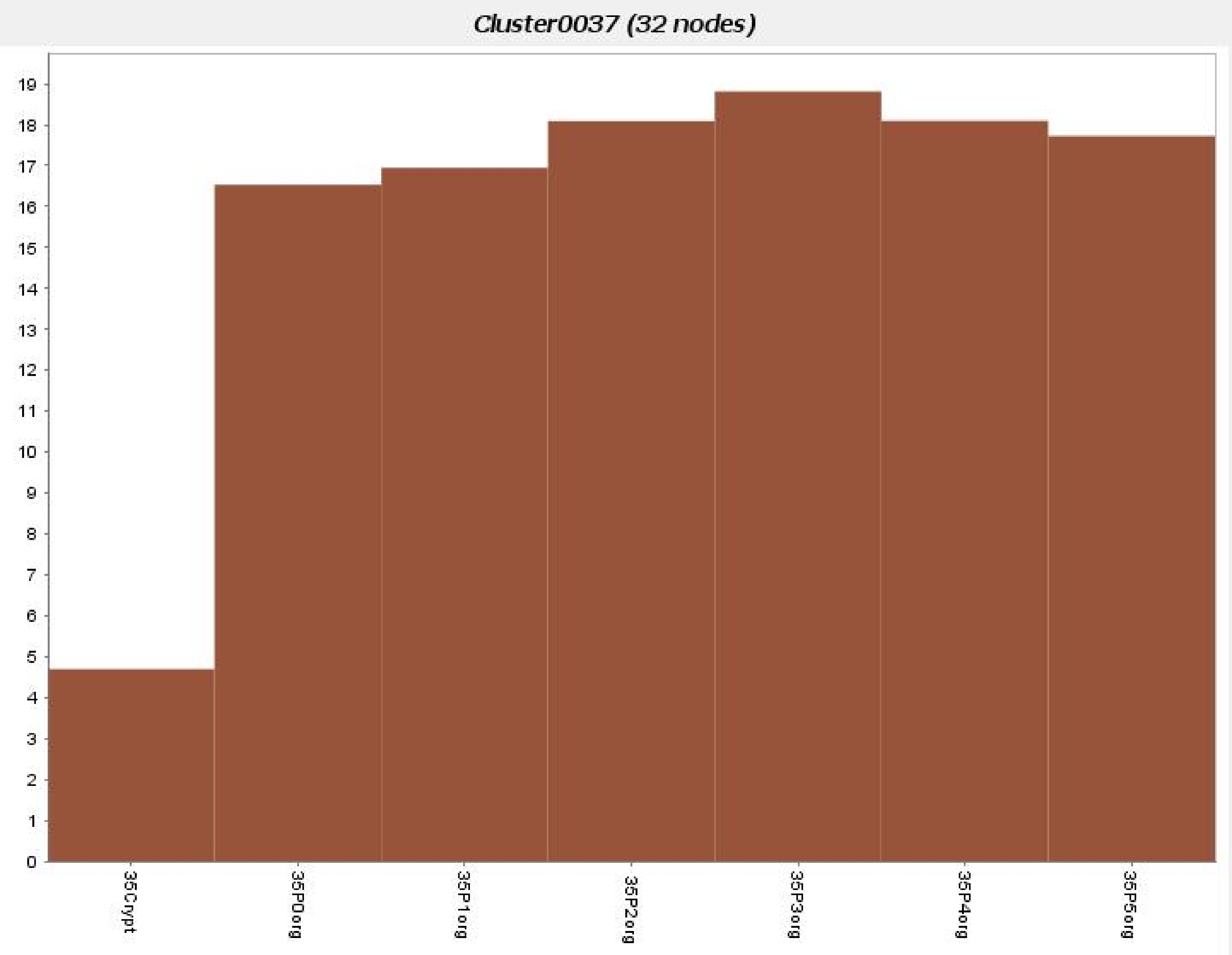

Cluster0037 (32 nodes)
0
1
2
3
4
5
6
7
8
9
10
11
12
13
14
15
16
17
18
19
35Crypt
35P0org
35P1org
35P2org
35P3org
35P4org
35P5org

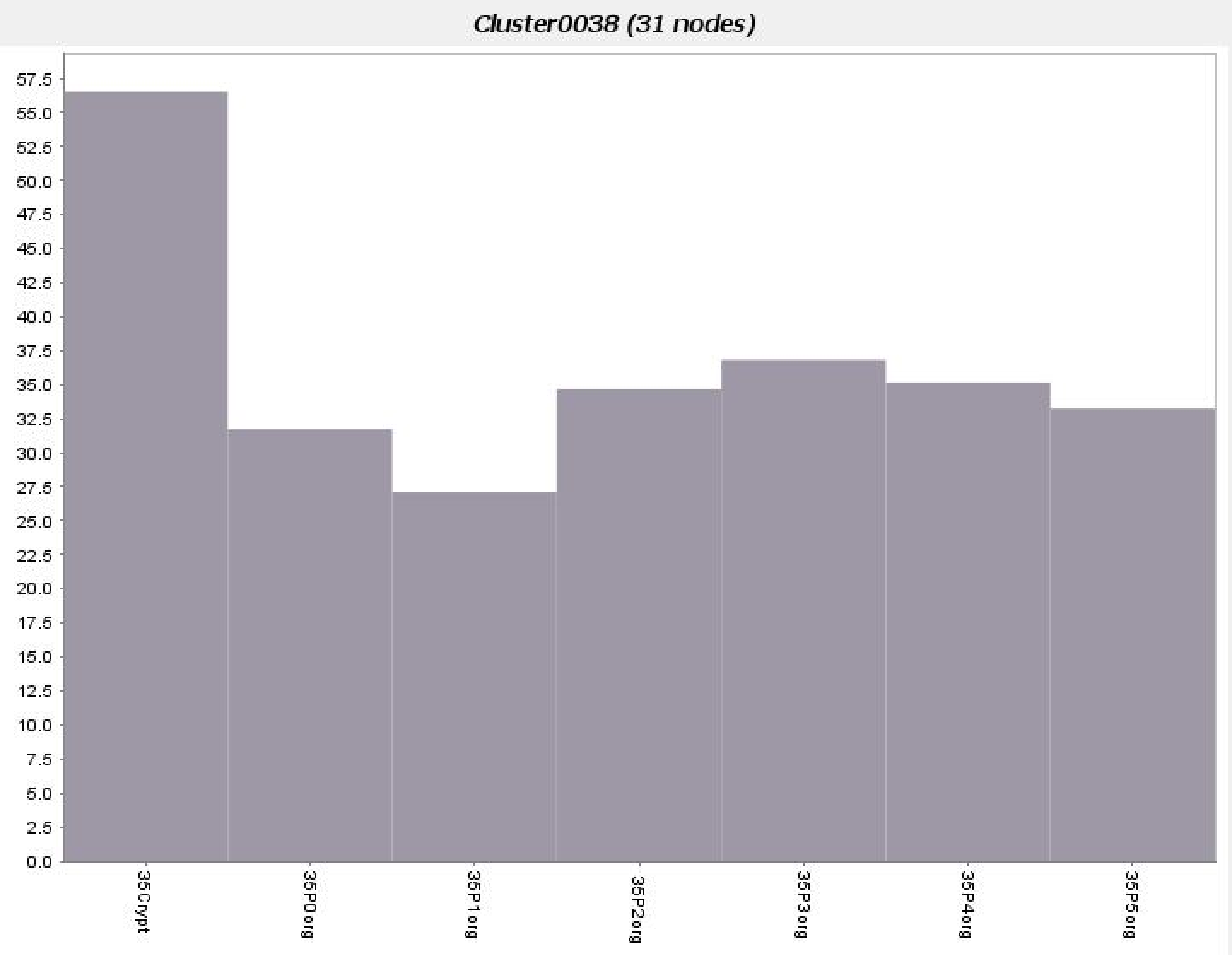
Cluster0038 (31 nodes)
57.5
55.0
52.5
50.0
47.5
45.0
42.5
40.0
37.5
35.0
32.5
30.0
27.5
25.0
22.5
20.0
17.5
15.0
12.5
10.0
7.5
5.0
2.5
0.0
35Crypt
35P0org
35P1org
35P2org
35P3org
35P4org
35P5org

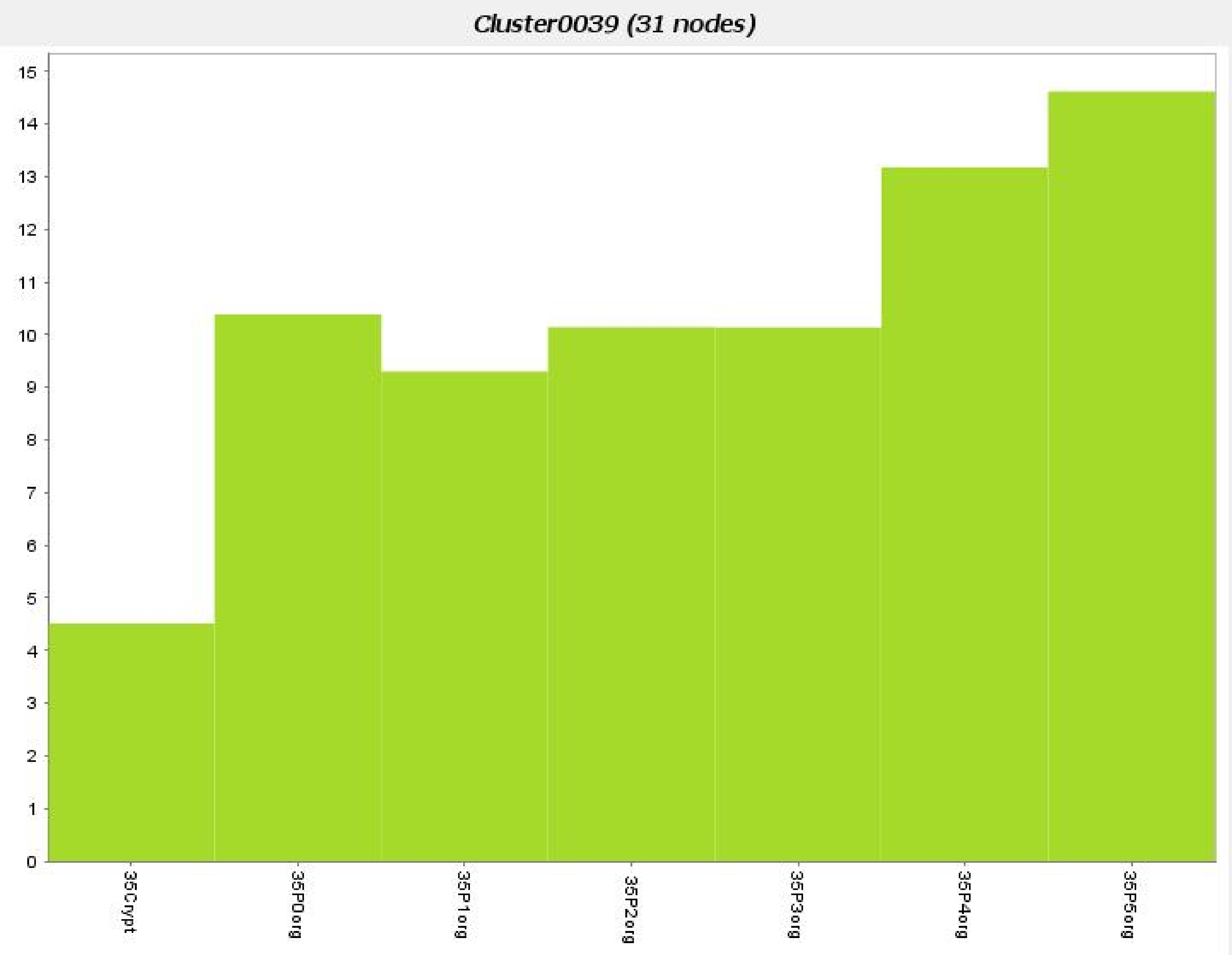
Cluster0039 (31 nodes)
0
1
2
3
4
5
6
7
8
9
10
11
12
13
14
15
35Crypt
35P0org
35P1org
35P2org
35P3org
35P4org
35P5org

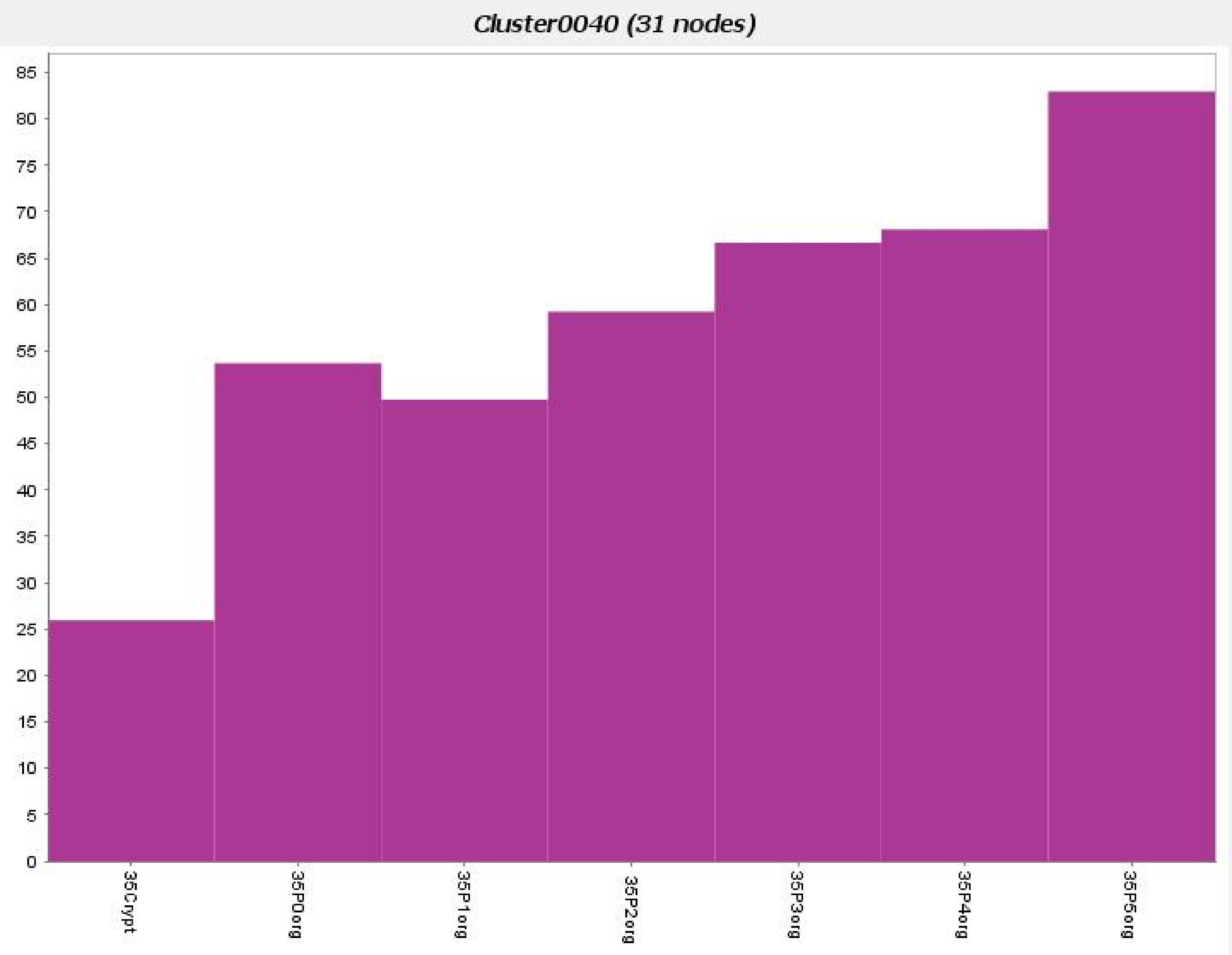
Cluster0040 (31 nodes)
85
80
75
70
65
60
55
50
45
40
35
30
25
20
15
10
5
0
35Crypt
35P0org
35P1org
35P2org
35P3org
35P4org
35P5org

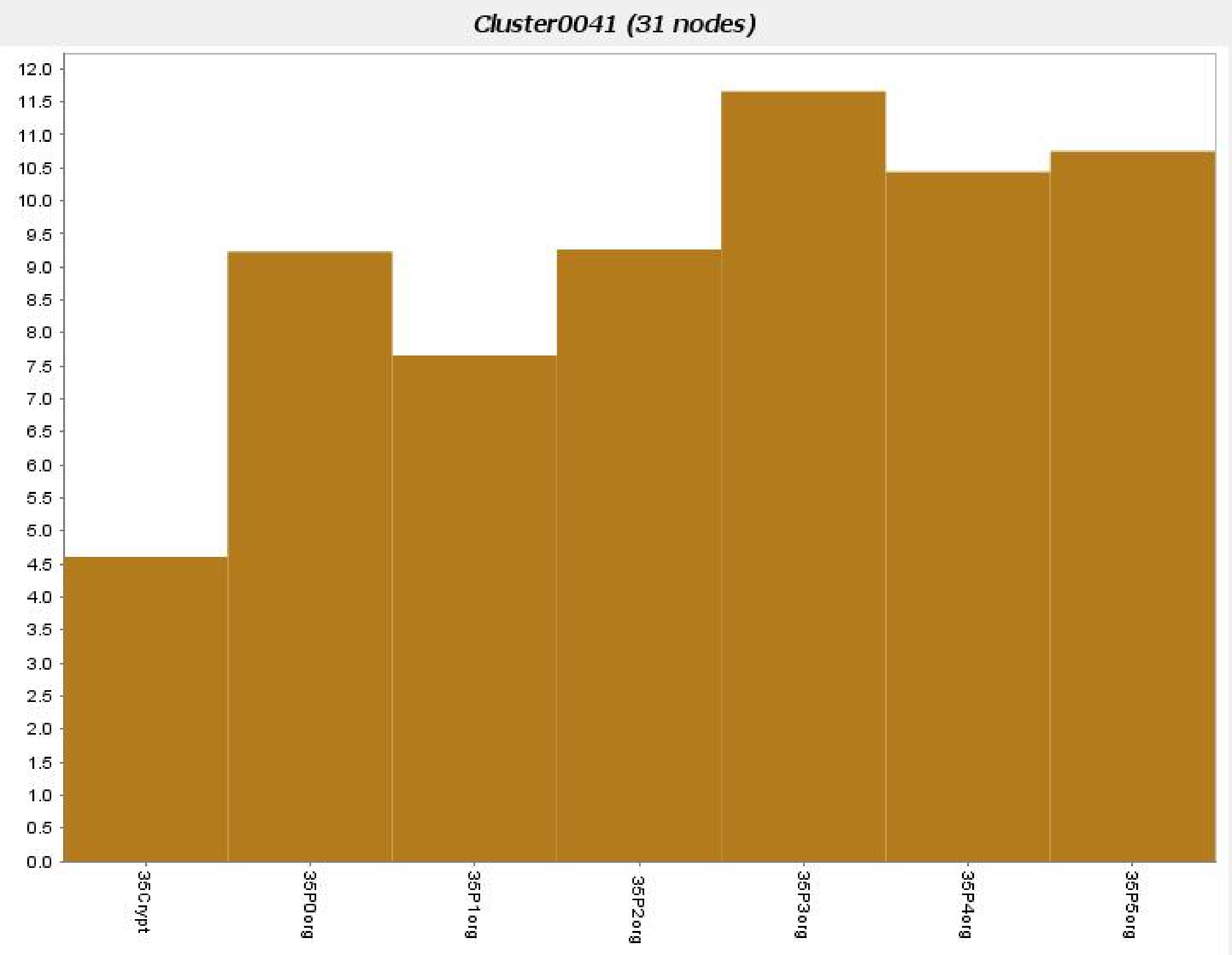
Cluster0041 (31 nodes)
12.0
11.5
11.0
10.5
10.0
9.5
9.0
8.5
8.0
7.5
7.0
6.5
6.0
5.5
5.0
4.5
4.0
3.5
3.0
2.5
2.0
1.5
1.0
0.5
0.0
35Crypt
35P0org
35P1org
35P2org
35P3org
35P4org
35P5org

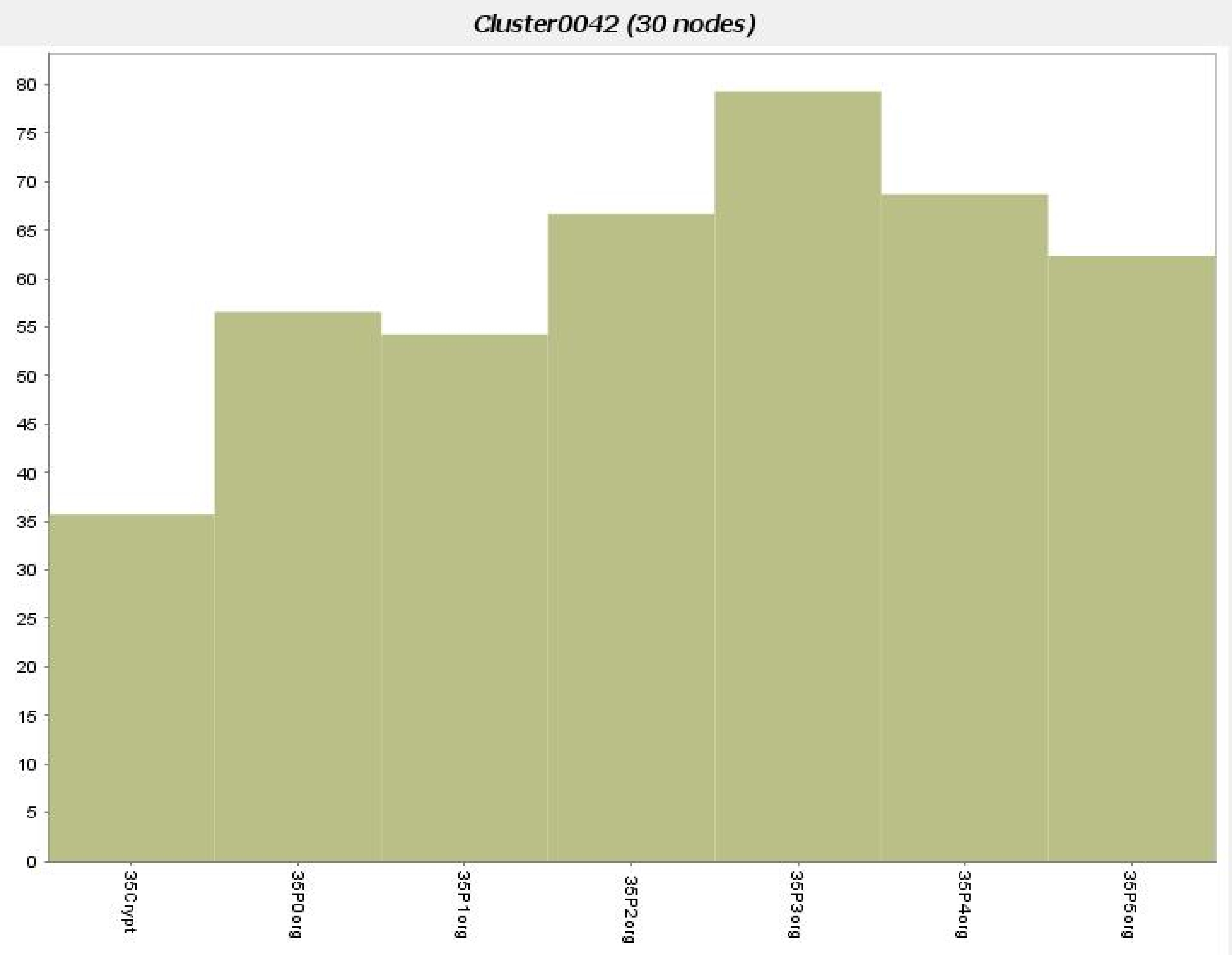
Cluster0042 (30 nodes)
0
5
10
15
20
25
30
35
40
45
50
55
60
65
70
75
80
35Crypt
35P0org
35P1org
35P2org
35P3org
35P4org
35P5org

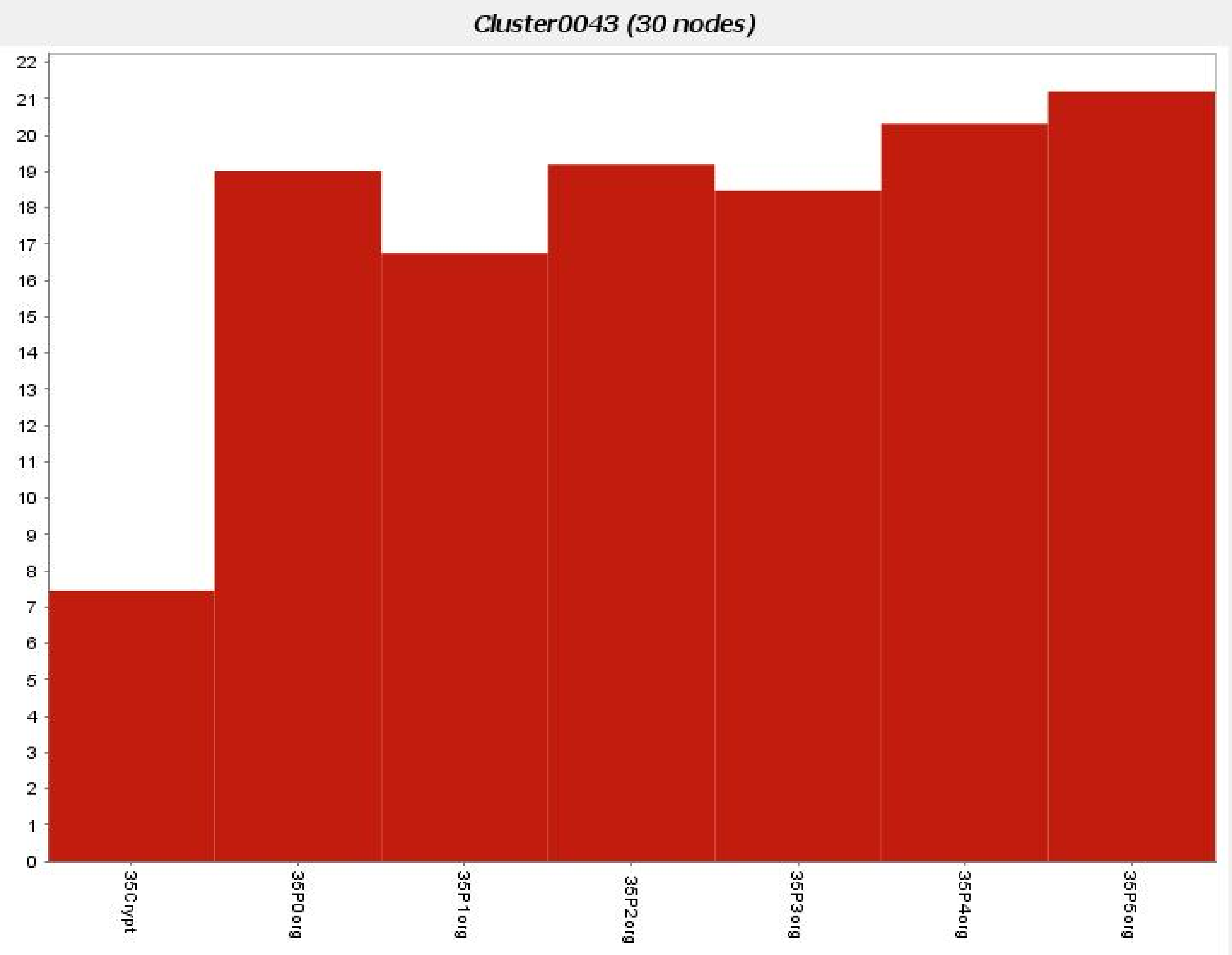
Cluster0043 (30 nodes)
0
1
2
3
4
5
6
7
8
9
10
11
12
13
14
15
16
17
18
19
20
21
22
35Crypt
35P0org
35P1org
35P2org
35P3org
35P4org
35P5org

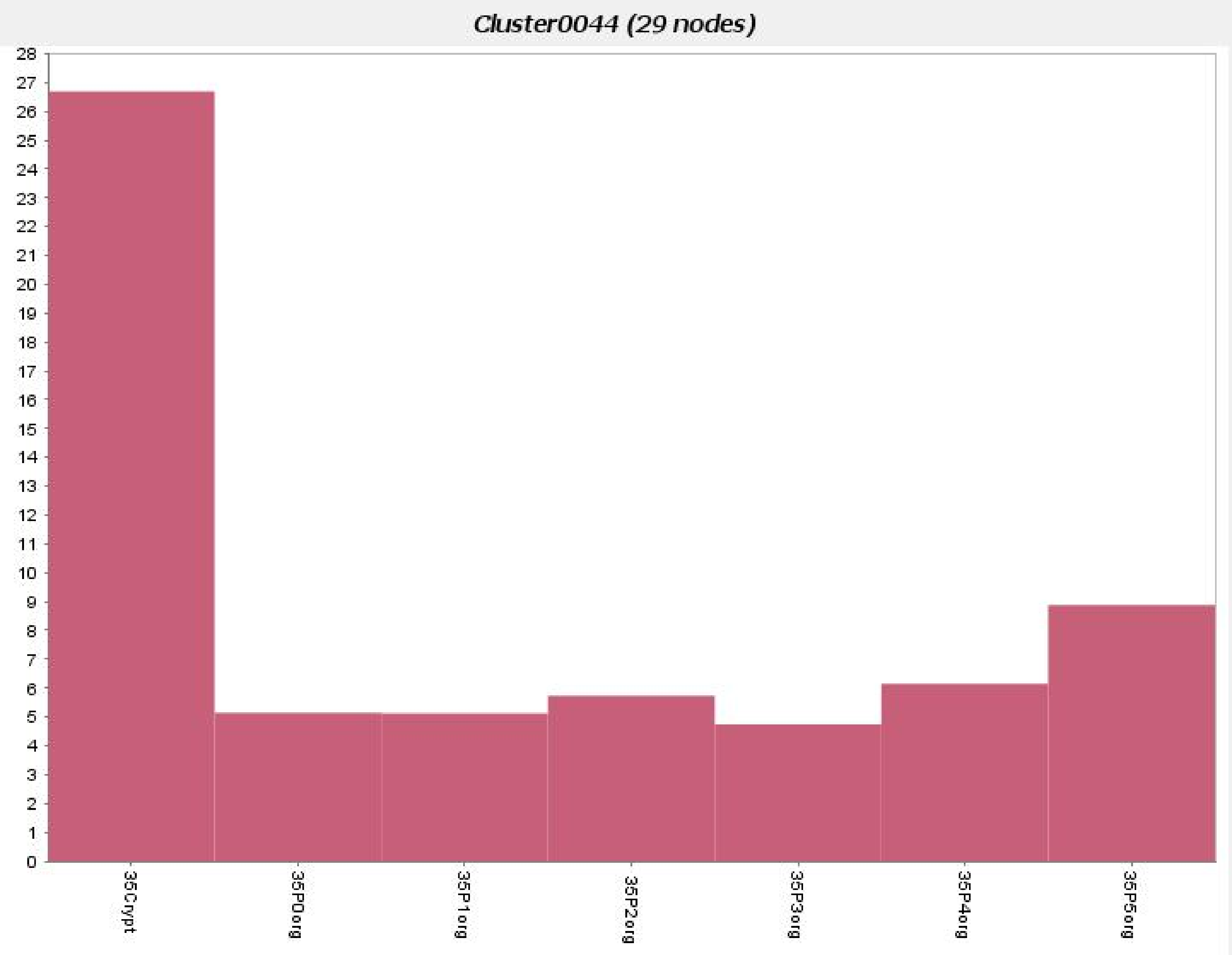

Cluster0044 (29 nodes)
28
27
26
25
24
23
22
21
20
19
18
17
16
15
14
13
12
11
10
9
8
7
6
5
4
3
2
1
0
35Crypt
35P0org
35P1org
35P2org
35P3org
35P4org
35P5org

Cluster0045 (28 nodes)

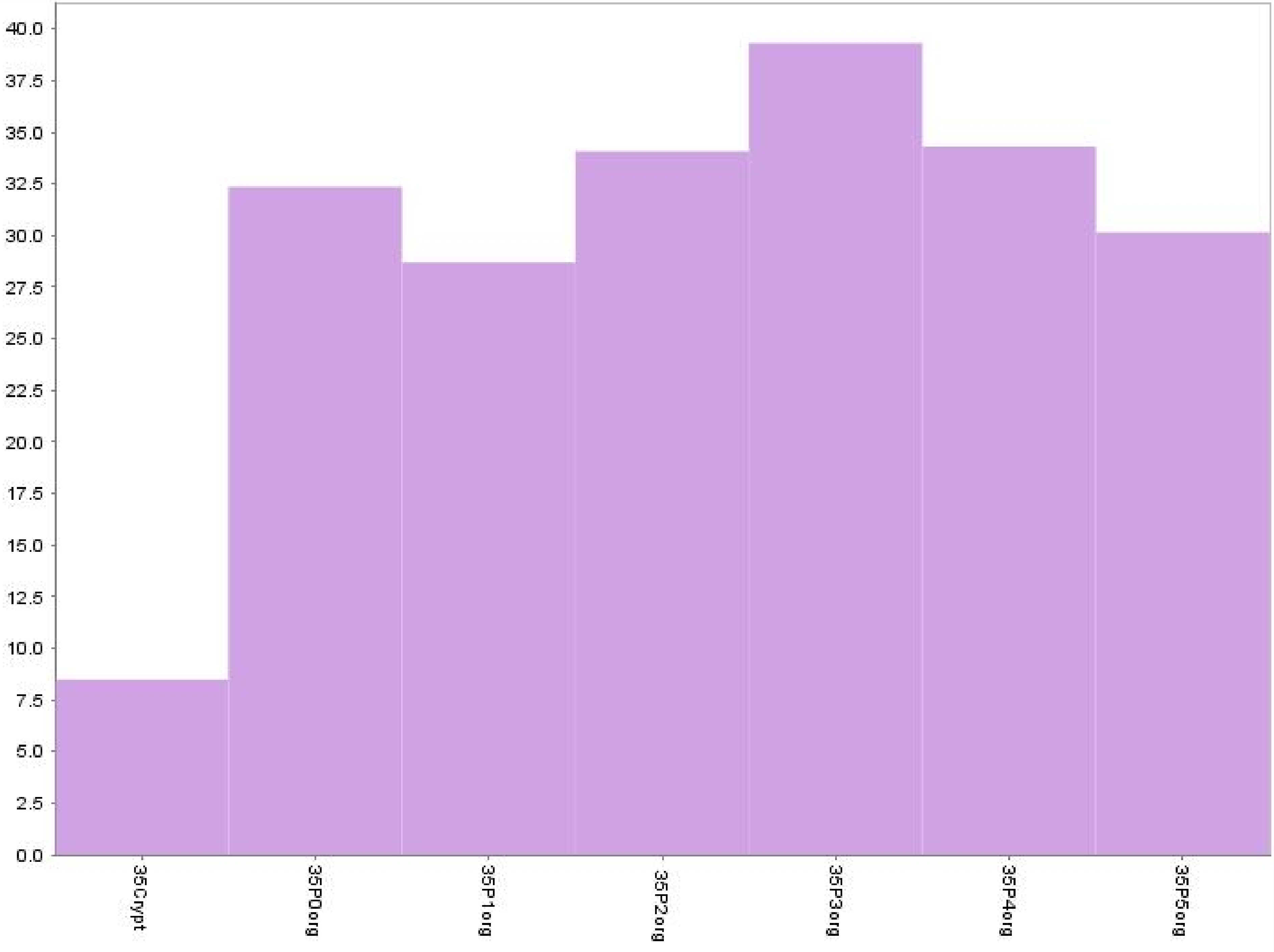

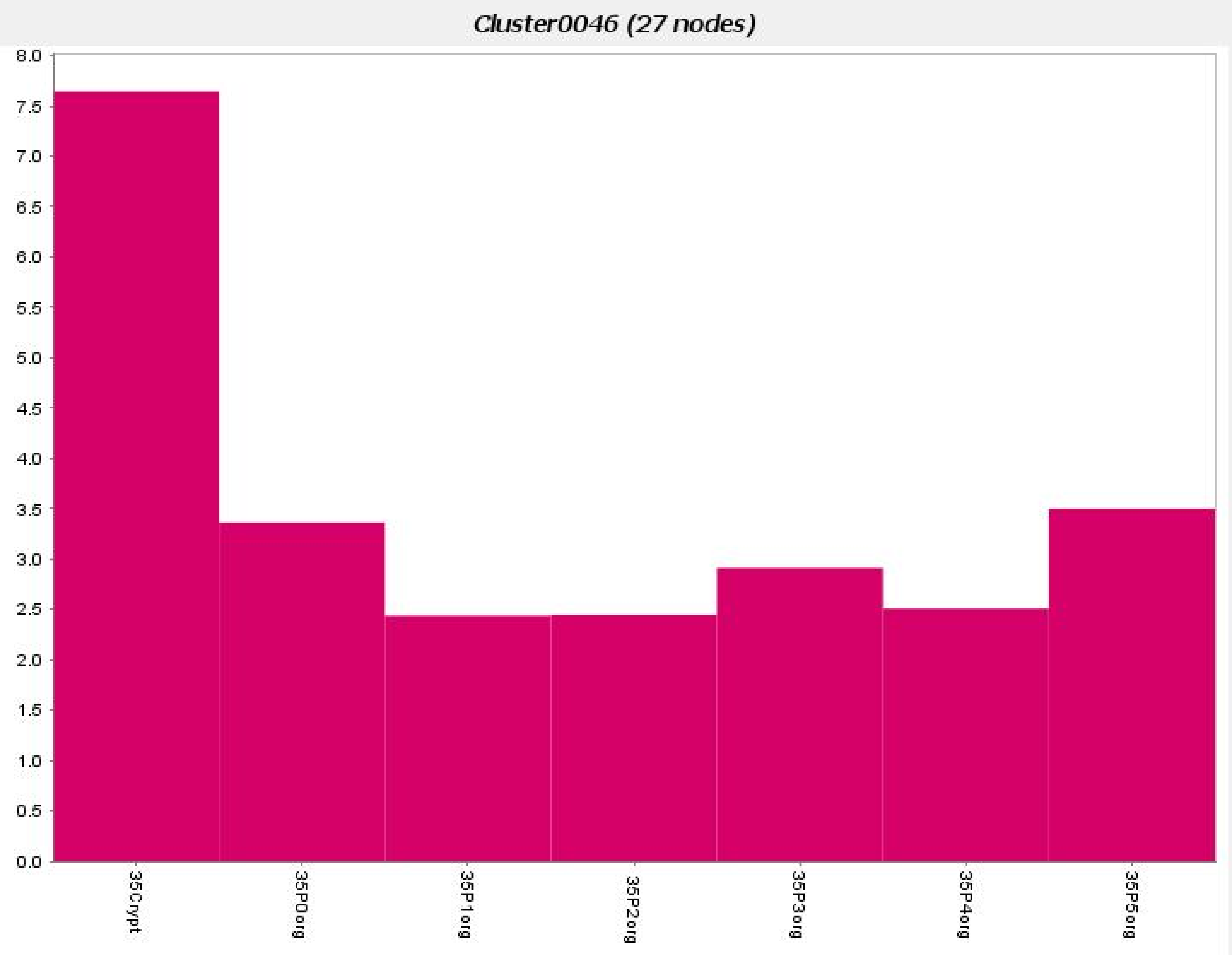
Cluster0046 (27 nodes)
8.0
7.5
7.0
6.5
6.0
5.5
5.0
4.5
4.0
3.5
3.0
2.5
2.0
1.5
1.0
0.5
0.0
35Crypt
35P0org
35P1org
35P2org
35P3org
35P4org
35P5org

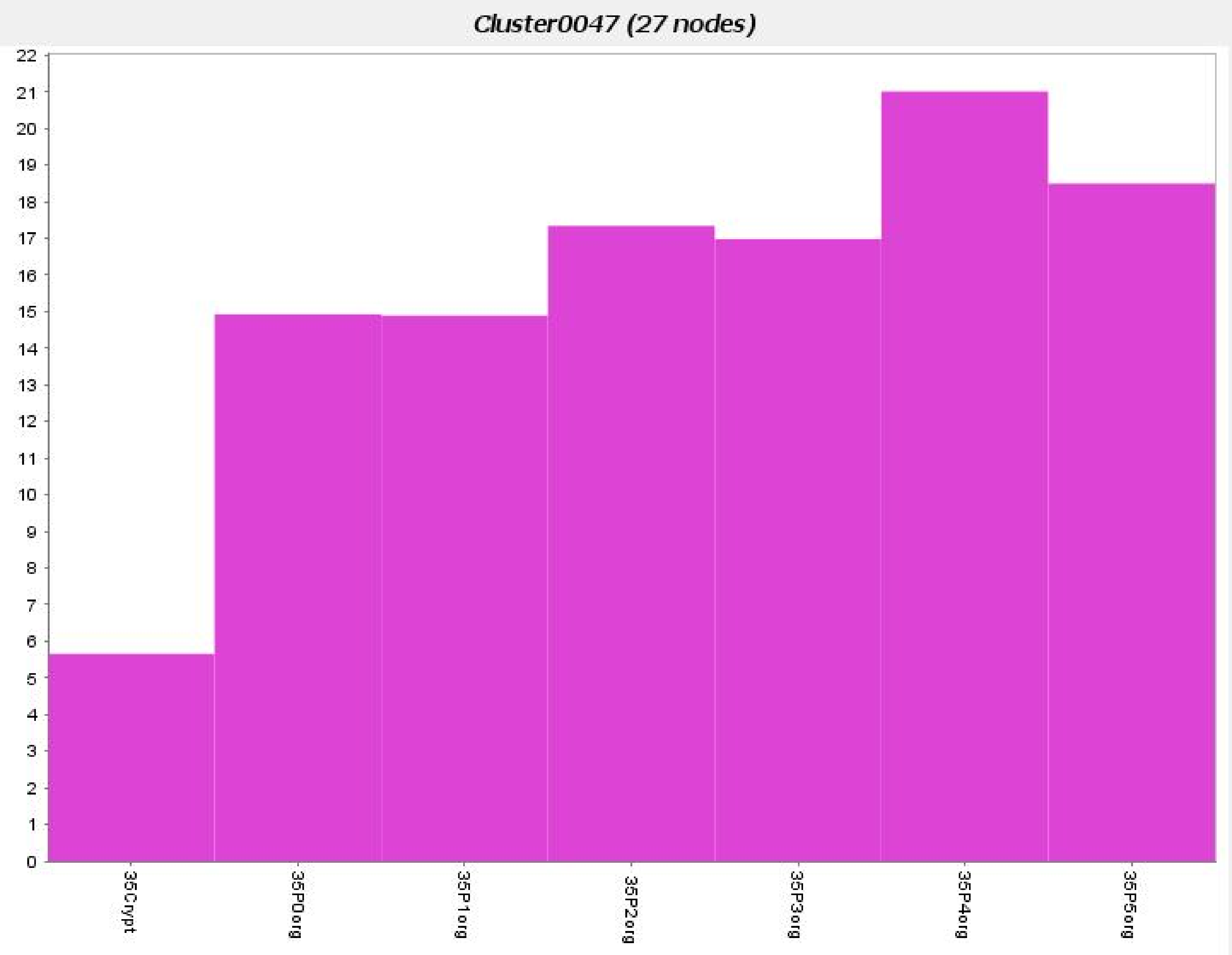

Cluster0047 (27 nodes)
22
21
20
19
18
17
16
15
14
13
12
11
10
9
8
7
6
5
4
3
2
1
0
35Crypt
35P0org
35P1org
35P2org
35P3org
35P4org
35P5org

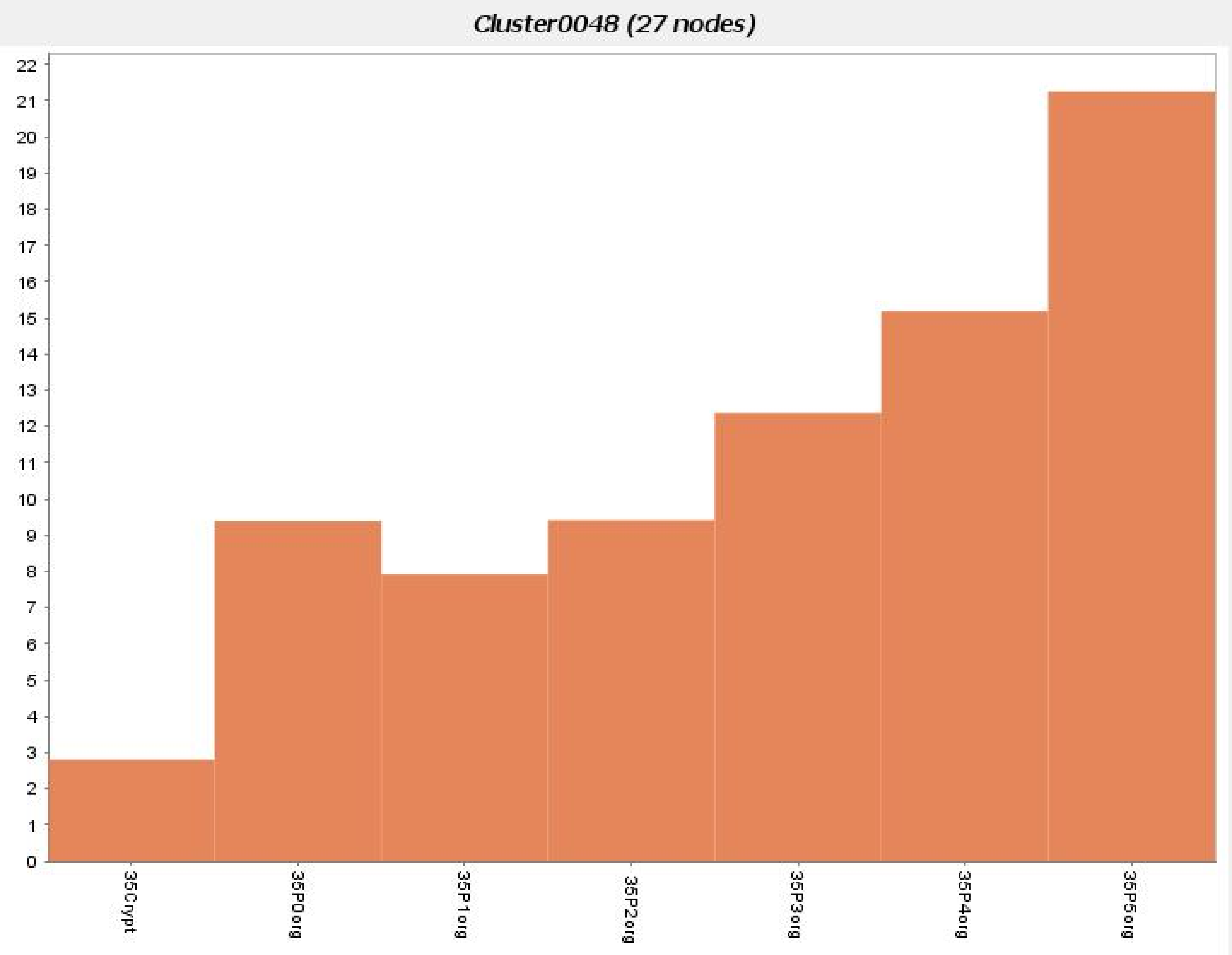

Cluster0048 (27 nodes)
22
21
20
19
18
17
16
15
14
13
12
11
10
9
8
7
6
5
4
3
2
1
0
35Crypt
35P0org
35P1org
35P2org
35P3org
35P4org
35P5org

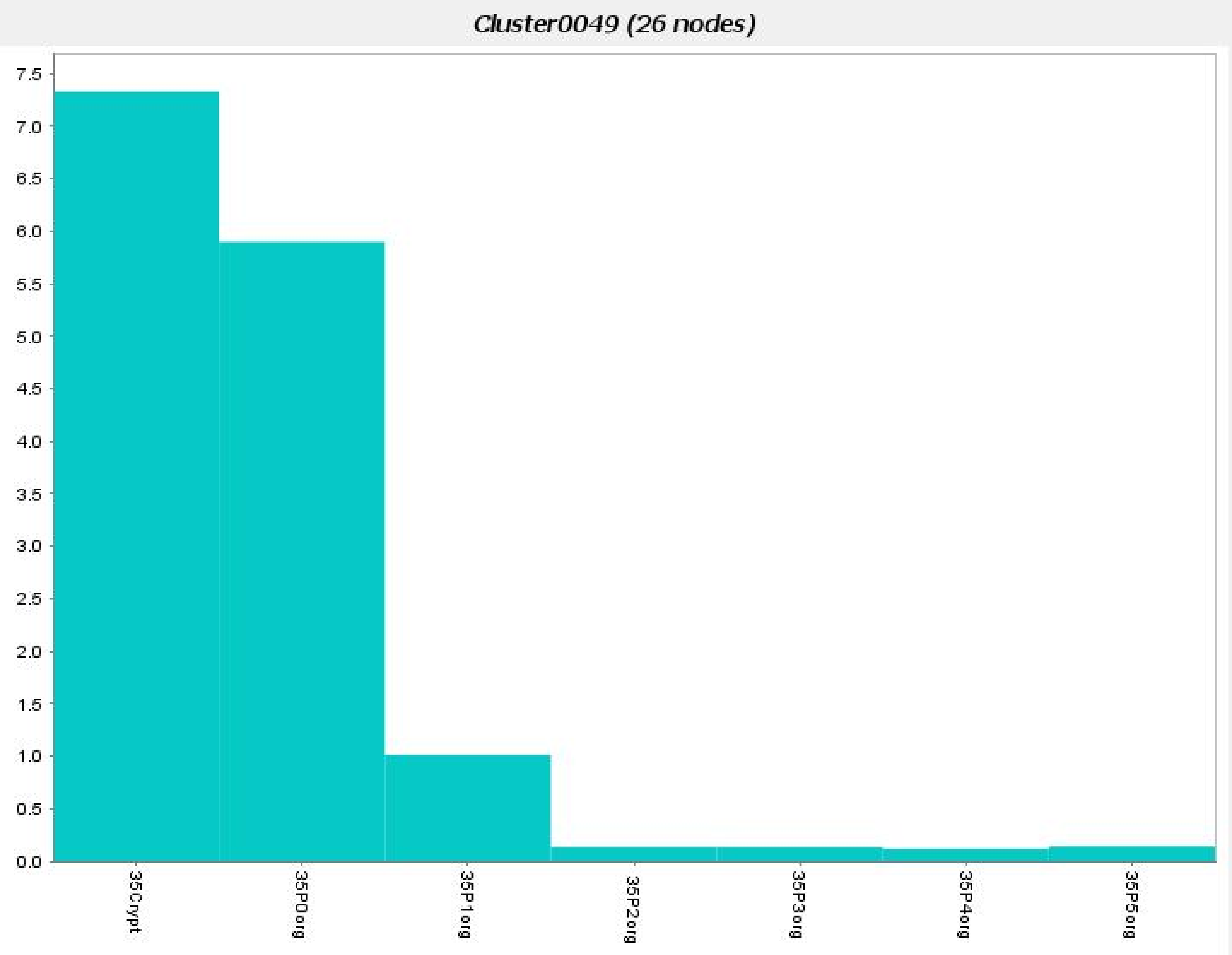
Cluster0049 (26 nodes)
7.5
7.0
6.5
6.0
5.5
5.0
4.5
4.0
3.5
3.0
2.5
2.0
1.5
1.0
0.5
0.0
35Crypt
35P0org
35P1org
35P2org
35P3org
35P4org
35P5org

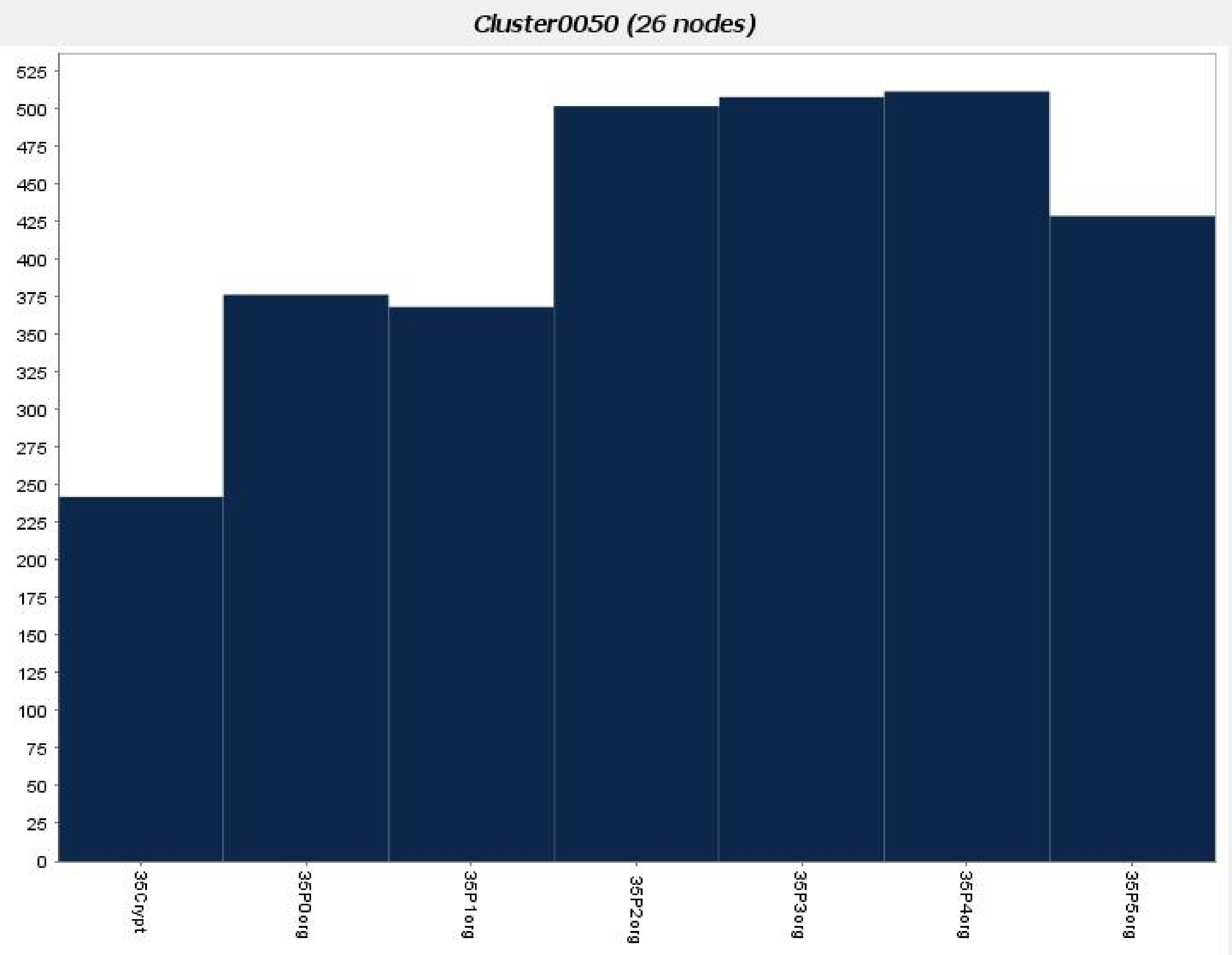

Cluster0050 (26 nodes)
525
500
475
450
425
400
375
350
325
300
275
250
225
200
175
150
125
100
75
50
25
0
35Crypt
35P0org
35P1org
35P2org
35P3org
35P4org
35P5org
