# Supplementary material for: Development of in vitro enteroids derived from bovine small intestinal crypts
Source: Vet Res. 2018 Jul 3;49:54. doi: 10.1186/s13567-018-0547-5 (PMC6029049; doi:10.1186/s13567-018-0547-5)
Supplement: Supplementary file 5 — Additional file 5. Comparison of stress-related gene expression in bovine enteroid cultures. Table comparing the relative expression level of a range of stress-related genes [13] in the enteroid cultures during serial subsequent rounds of passage. P0, freshly prepared enteroids; P1, passage 1 enteroids, etc. [file 13567_2018_547_MOESM5_ESM.pdf]

Additional File 5: Differential expression of stress-related genes

<table>
<tr><th></th><th></th><th></th><th colspan="5">Relative gene expression level in PX enteroids vs. P0 organoids</th></tr>
<tr><th>Gene symbol</th><th>Target ID</th><th>Description</th><th>P1 enteroids</th><th>P2 enteroids</th><th>P3 enteroids</th><th>P4 enteroids</th><th>P5 enteroids</th></tr>
<tr><td>ABCB4</td><td>gi|741923343|ref|XM_010804130.1|</td><td>PREDICTED: Bos taurus ATP-binding cassette, sub-family B (MDR/TAP), member 4 (ABCB4), transcript variant X1, mRNA</td><td>n.a.</td><td>n.a.</td><td>n.a.</td><td>n.a.</td><td>n.a.</td></tr>
<tr><td>ABCB4</td><td>gi|982921203|ref|XM_010804131.2|</td><td>PREDICTED: Bos taurus ATP-binding cassette, sub-family B (MDR/TAP), member 4 (ABCB4), transcript variant X2, mRNA</td><td>n.a.</td><td>n.a.</td><td>n.a.</td><td>n.a.</td><td>n.a.</td></tr>
<tr><td>ABCB4</td><td>gi|982921204|ref|XM_015468706.1|</td><td>PREDICTED: Bos taurus ATP-binding cassette, sub-family B (MDR/TAP), member 4 (ABCB4), transcript variant X3, mRNA</td><td>n.a.</td><td>n.a.</td><td>n.a.</td><td>n.a.</td><td>n.a.</td></tr>
<tr><td>ABCB4</td><td>gi|982921206|ref|XM_015468707.1|</td><td>PREDICTED: Bos taurus ATP-binding cassette, sub-family B (MDR/TAP), member 4 (ABCB4), transcript variant X4, mRNA</td><td>n.a.</td><td>n.a.</td><td>n.a.</td><td>n.a.</td><td>n.a.</td></tr>
<tr><td>ABCB4</td><td>gi|982921208|ref|XM_005205302.3|</td><td>PREDICTED: Bos taurus ATP-binding cassette, sub-family B (MDR/TAP), member 4 (ABCB4), transcript variant X5, mRNA</td><td>n.a.</td><td>n.a.</td><td>n.a.</td><td>n.a.</td><td>n.a.</td></tr>
<tr><td>ABCB4</td><td>gi|982921210|ref|XM_015468709.1|</td><td>PREDICTED: Bos taurus ATP-binding cassette, sub-family B (MDR/TAP), member 4 (ABCB4), transcript variant X6, mRNA</td><td>n.a.</td><td>n.a.</td><td>n.a.</td><td>n.a.</td><td>n.a.</td></tr>
<tr><td>ABCB4</td><td>gi|982921212|ref|XM_015468710.1|</td><td>PREDICTED: Bos taurus ATP-binding cassette, sub-family B (MDR/TAP), member 4 (ABCB4), transcript variant X7, mRNA</td><td>n.a.</td><td>n.a.</td><td>n.a.</td><td>n.a.</td><td>n.a.</td></tr>
<tr><td>ABCB4</td><td>gi|982921214|ref|XM_005205304.3|</td><td>PREDICTED: Bos taurus ATP-binding cassette, sub-family B (MDR/TAP), member 4 (ABCB4), transcript variant X8, mRNA</td><td>n.a.</td><td>n.a.</td><td>n.a.</td><td>n.a.</td><td>n.a.</td></tr>
<tr><td>ABCB4</td><td>gi|982921215|ref|XM_005205305.3|</td><td>PREDICTED: Bos taurus ATP-binding cassette, sub-family B (MDR/TAP), member 4 (ABCB4), transcript variant X9, mRNA</td><td>n.a.</td><td>n.a.</td><td>n.a.</td><td>n.a.</td><td>n.a.</td></tr>
<tr><td>APEX1</td><td>gi|223278399|ref|NM_176609.3|</td><td>Bos taurus APEX nuclease (multifunctional DNA repair enzyme) 1 (APEX1), mRNA</td><td>0.941004161</td><td>1.218538713</td><td>1.102082215</td><td>0.960643136</td><td>0.854576193</td><td>n</td></tr>
<tr><td>ATM</td><td>gi|329664119|ref|NM_001205935.1|</td><td>Bos taurus ATM serine/threonine kinase (ATM), mRNA</td><td>0.614095638</td><td>1.239859288</td><td>0.937575196</td><td>1.092183135</td><td>0.989668562</td></tr>
<tr><td>ATM</td><td>gi|982945403|ref|XM_010812321.2|</td><td>PREDICTED: Bos taurus ATM serine/threonine kinase (ATM), transcript variant X1, mRNA</td><td>n.a.</td><td>n.a.</td><td>n.a.</td><td>n.a.</td><td>n.a.</td></tr>
<tr><td>ATM</td><td>gi|982945404|ref|XM_010812322.2|</td><td>PREDICTED: Bos taurus ATM serine/threonine kinase (ATM), transcript variant X2, mRNA</td><td>0.906421419</td><td>1.014013288</td><td>1.11273023</td><td>1.093799876</td><td>1.174491696</td></tr>
<tr><td>ATM</td><td>gi|982945405|ref|XM_010812323.2|</td><td>PREDICTED: Bos taurus ATM serine/threonine kinase (ATM), transcript variant X3, mRNA</td><td>1.129486826</td><td>1.390236937</td><td>0.907886399</td><td>1.334693463</td><td>0.760738893</td></tr>
<tr><td>ATM</td><td>gi|982945406|ref|XM_010812324.2|</td><td>PREDICTED: Bos taurus ATM serine/threonine kinase (ATM), transcript variant X4, mRNA</td><td>1.037419226</td><td>1.216673781</td><td>1.108224767</td><td>1.042114249</td><td>0.681959519</td></tr>
<tr><td>ATM</td><td>gi|982945407|ref|XM_010812325.2|</td><td>PREDICTED: Bos taurus ATM serine/threonine kinase (ATM), transcript variant X5, mRNA</td><td>0.905940741</td><td>1.42829218</td><td>0.998973224</td><td>0.760958912</td><td>1.934796479</td></tr>
<tr><td>ATM</td><td>gi|982945408|ref|XM_005215785.3|</td><td>PREDICTED: Bos taurus ATM serine/threonine kinase (ATM), transcript variant X6, mRNA</td><td>0.934635255</td><td>0.972819367</td><td>0.991617626</td><td>1.050223942</td><td>1.121755153</td></tr>
<tr><td>BRCA1</td><td>gi|30466259|ref|NM_178573.1|</td><td>Bos taurus breast cancer 1, early onset (BRCA1), mRNA</td><td>0.987004883</td><td>1.044260865</td><td>1.074959595</td><td>0.992371639</td><td>0.905436293</td></tr>
<tr><td>BRCA1</td><td>gi|982957762|ref|XM_005220676.3|</td><td>PREDICTED: Bos taurus breast cancer 1 (BRCA1), transcript variant X2, mRNA</td><td>0.783053026</td><td>1.323566554</td><td>1.037284062</td><td>0.994343077</td><td>0.782486876</td></tr>
<tr><td>BRCA1</td><td>gi|982957763|ref|XM_005220678.3|</td><td>PREDICTED: Bos taurus breast cancer 1 (BRCA1), transcript variant X3, mRNA</td><td>0.581986689</td><td>1.1209527</td><td>1.171468297</td><td>1.277743096</td><td>1.081355441</td></tr>
<tr><td>BRCA1</td><td>gi|982957764|ref|XM_015458822.1|</td><td>PREDICTED: Bos taurus breast cancer 1 (BRCA1), transcript variant X4, mRNA</td><td>n.a.</td><td>n.a.</td><td>n.a.</td><td>n.a.</td><td>n.a.</td></tr>
<tr><td>BRCA1</td><td>gi|982957766|ref|XM_005220679.3|</td><td>PREDICTED: Bos taurus breast cancer 1 (BRCA1), transcript variant X5, mRNA</td><td>0.967481196</td><td>1.169317092</td><td>1.067929535</td><td>0.945017519</td><td>0.977018236</td></tr>
<tr><td>BRCA2</td><td>gi|982940963|ref|XM_002691807.4|</td><td>PREDICTED: Bos taurus breast cancer 2 (BRCA2), transcript variant X1, mRNA</td><td>0.68758031</td><td>0.914749592</td><td>1.003389542</td><td>1.048778475</td><td>0.840848561</td></tr>
<tr><td>BRCA2</td><td>gi|982940965|ref|XM_010810770.2|</td><td>PREDICTED: Bos taurus breast cancer 2 (BRCA2), transcript variant X2, mRNA</td><td>0.736326236</td><td>1.183361899</td><td>0.951288219</td><td>1.363235151</td><td>1.043404781</td></tr>
<tr><td>BRCA2</td><td>gi|982991784|ref|XM_015465653.1|</td><td>PREDICTED: Bos taurus breast cancer 2 (BRCA2), transcript variant X3, mRNA</td><td>0.86317146</td><td>1.024222298</td><td>0.994157827</td><td>1.462236012</td><td>0.979945445</td></tr>
<tr><td>CANX</td><td>gi|157785566|ref|NM_001105612.1|</td><td>Bos taurus calnexin (CANX), mRNA</td><td>0.916143335</td><td>1.25955077</td><td>1.106197682</td><td>0.990320785</td><td>0.98528275</td></tr>
<tr><td>CAT</td><td>gi|402693375|ref|NM_001035386.2|</td><td>Bos taurus catalase (CAT), mRNA</td><td>0.879520819</td><td>1.28425293</td><td>1.103907745</td><td>0.919406966</td><td>0.912187961</td></tr>
<tr><td>CCT2</td><td>gi|77735434|ref|NM_001034239.1|</td><td>Bos taurus chaperonin containing TCP1, subunit 2 (beta) (CCT2), mRNA</td><td>1.021073764</td><td>1.120156594</td><td>1.097878638</td><td>0.982465601</td><td>1.023716214</td></tr>
<tr><td>CCT3</td><td>gi|402692562|ref|NM_001017934.3|</td><td>Bos taurus chaperonin containing TCP1, subunit 3 (gamma) (CCT3), mRNA</td><td>0.9732394</td><td>1.177788383</td><td>1.083467635</td><td>1.012361968</td><td>1.004478479</td></tr>
<tr><td>CCT4</td><td>gi|84000360|ref|NM_001038194.1|</td><td>Bos taurus chaperonin containing TCP1, subunit 4 (delta) (CCT4), mRNA</td><td>0.995029551</td><td>1.161214607</td><td>1.119943228</td><td>0.960211739</td><td>0.922657423</td></tr>
<tr><td>CCT5</td><td>gi|274325363|ref|NM_001034595.2|</td><td>Bos taurus chaperonin containing TCP1, subunit 5 (epsilon) (CCT5), mRNA</td><td>1.073220665</td><td>1.097720761</td><td>1.087498356</td><td>1.019665765</td><td>1.026254339</td></tr>
<tr><td>CCT6A</td><td>gi|148233485|ref|NM_001034542.2|</td><td>Bos taurus chaperonin containing TCP1, subunit 6A (zeta 1) (CCT6A), mRNA</td><td>1.032463291</td><td>1.053841288</td><td>1.080741383</td><td>1.036377475</td><td>0.994316415</td></tr>
<tr><td>CCT6B</td><td>gi|402744928|ref|NM_001034642.2|</td><td>Bos taurus chaperonin containing TCP1, subunit 6B (zeta 2) (CCT6B), mRNA</td><td>0.681037215</td><td>1.292493862</td><td>1.374321196</td><td>1.225848108</td><td>0.74146284</td></tr>
<tr><td>CCT6B</td><td>gi|982956086|ref|XM_005220022.3|</td><td>PREDICTED: Bos taurus chaperonin containing TCP1 subunit 6B (CCT6B), transcript variant X1, mRNA</td><td>n.a.</td><td>n.a.</td><td>n.a.</td><td>n.a.</td><td>n.a.</td></tr>
<tr><td>CCT6B</td><td>gi|982956087|ref|XM_005220024.3|</td><td>PREDICTED: Bos taurus chaperonin containing TCP1 subunit 6B (CCT6B), transcript variant X2, mRNA</td><td>n.a.</td><td>n.a.</td><td>n.a.</td><td>n.a.</td><td>n.a.</td></tr>
<tr><td>CCT6B</td><td>gi|982956089|ref|XM_010815775.2|</td><td>PREDICTED: Bos taurus chaperonin containing TCP1 subunit 6B (CCT6B), transcript variant X3, mRNA</td><td>n.a.</td><td>n.a.</td><td>n.a.</td><td>n.a.</td><td>n.a.</td></tr>
<tr><td>CCT6B</td><td>gi|982956090|ref|XM_015458544.1|</td><td>PREDICTED: Bos taurus chaperonin containing TCP1 subunit 6B (CCT6B), transcript variant X4, mRNA</td><td>n.a.</td><td>n.a.</td><td>n.a.</td><td>n.a.</td><td>n.a.</td></tr>
<tr><td>CCT6B</td><td>gi|982956093|ref|XM_015458545.1|</td><td>PREDICTED: Bos taurus chaperonin containing TCP1 subunit 6B (CCT6B), transcript variant X5, mRNA</td><td>n.a.</td><td>n.a.</td><td>n.a.</td><td>n.a.</td><td>n.a.</td></tr>
<tr><td>CCT7</td><td>gi|402692506|ref|NM_001046171.2|</td><td>Bos taurus chaperonin containing TCP1, subunit 7 (eta) (CCT7), mRNA</td><td>1.006891526</td><td>1.127686063</td><td>1.083682503</td><td>0.964893743</td><td>1.006084386</td></tr>
<tr><td>CCT8</td><td>gi|75812921|ref|NM_001033609.1|</td><td>Bos taurus chaperonin containing TCP1, subunit 8 (theta) (CCT8), mRNA</td><td>0.998205914</td><td>1.12318961</td><td>1.099425139</td><td>0.986212878</td><td>1.027009542</td></tr>
<tr><td>CCT8</td><td>gi|982909446|ref|XM_005201119.3|</td><td>PREDICTED: Bos taurus chaperonin containing TCP1 subunit 8 (CCT8), transcript variant X1, mRNA</td><td>0.821227862</td><td>1.081922544</td><td>1.348590066</td><td>1.03409237</td><td>0.874509293</td></tr>
<tr><td>CDKN1A</td><td>gi|402692179|ref|NM_001098958.2|</td><td>Bos taurus cyclin-dependent kinase inhibitor 1A (p21, Cip1) (CDKN1A), mRNA</td><td>0.665424068</td><td>1.618652958</td><td>1.467499816</td><td>0.553772382</td><td>1.033420244</td></tr>
<tr><td>CDKN1A</td><td>gi|982963239|ref|XM_005223326.3|</td><td>PREDICTED: Bos taurus cyclin-dependent kinase inhibitor 1A (p21, Cip1) (CDKN1A), transcript variant X1, mRNA</td><td>0.733788884</td><td>1.587107836</td><td>1.354858865</td><td>0.6711378</td><td>1.054940655</td></tr>
<tr><td>CLGN</td><td>gi|402691836|ref|NM_001034205.2|</td><td>Bos taurus calmegin (CLGN), mRNA</td><td>n.a.</td><td>n.a.</td><td>n.a.</td><td>n.a.</td><td>n.a.</td></tr>
<tr><td>CRYBA1</td><td>gi|402692289|ref|NM_174523.3|</td><td>Bos taurus crystallin, beta A1 (CRYBA1), mRNA</td><td>n.a.</td><td>n.a.</td><td>n.a.</td><td>n.a.</td><td>n.a.</td></tr>
<tr><td>CRYBA2</td><td>gi|31341762|ref|NM_174524.2|</td><td>Bos taurus crystallin, beta A2 (CRYBA2), mRNA</td><td>n.a.</td><td>n.a.</td><td>n.a.</td><td>n.a.</td><td>n.a.</td></tr>
<tr><td>CRYGA</td><td>gi|343780969|ref|NM_001243570.1|</td><td>Bos taurus crystallin, gamma A (CRYGA), mRNA</td><td>n.a.</td><td>n.a.</td><td>n.a.</td><td>n.a.</td><td>n.a.</td></tr>
<tr><td>CRYZ</td><td>gi|114326273|ref|NM_174025.3|</td><td>Bos taurus crystallin, zeta (quinone reductase) (CRYZ), mRNA</td><td>1.047774101</td><td>1.186069033</td><td>1.022079164</td><td>1.023456293</td><td>0.841724335</td></tr>
<tr><td>CYP17A1</td><td>gi|451958132|ref|NM_174304.2|</td><td>Bos taurus cytochrome P450, family 17, subfamily A, polypeptide 1 (CYP17A1), mRNA</td><td>n.a.</td><td>n.a.</td><td>n.a.</td><td>n.a.</td><td>n.a.</td></tr>
<tr><td>CYP1A2</td><td>gi|982960435|ref|XM_010817139.2|</td><td>PREDICTED: Bos taurus cytochrome P450, family 1, subfamily A, polypeptide 2 (CYP1A2), transcript variant X1, mRNA</td><td>n.a.</td><td>n.a.</td><td>n.a.</td><td>n.a.</td><td>n.a.</td></tr>
<tr><td>CYP24A1</td><td>gi|300794134|ref|NM_001191417.1|</td><td>Bos taurus cytochrome P450, family 24, subfamily A, polypeptide 1 (CYP24A1), nuclear gene encoding mitochondrial protein, mRN</td><td>n.a.</td><td>n.a.</td><td>n.a.</td><td>n.a.</td><td>n.a.</td></tr>
<tr><td>CYP2E1</td><td>gi|724470721|ref|NM_174530.3|</td><td>Bos taurus cytochrome P450, family 2, subfamily E, polypeptide 1 (CYP2E1), mRNA</td><td>n.a.</td><td>n.a.</td><td>n.a.</td><td>n.a.</td><td>n.a.</td></tr>
<tr><td>CYP4B1</td><td>gi|402745168|ref|NM_001076202.2|</td><td>Bos taurus cytochrome P450, family 4, subfamily B, polypeptide 1 (CYP4B1), mRNA</td><td>n.a.</td><td>n.a.</td><td>n.a.</td><td>n.a.</td><td>n.a.</td></tr>
<tr><td>CYP7A1</td><td>gi|329664101|ref|NM_001205677.1|</td><td>Bos taurus cytochrome P450, family 7, subfamily A, polypeptide 1 (CYP7A1), mRNA</td><td>n.a.</td><td>n.a.</td><td>n.a.</td><td>n.a.</td><td>n.a.</td></tr>
<tr><td>DDIT3</td><td>gi|118151443|ref|NM_001078163.1|</td><td>Bos taurus DNA-damage-inducible transcript 3 (DDIT3), mRNA</td><td>0.90172702</td><td>1.193414253</td><td>0.80703473</td><td>0.996768982</td><td>0.98345613</td></tr>
<tr><td>DNAJC1</td><td>gi|982942340|ref|XM_015474003.1|</td><td>PREDICTED: Bos taurus DnaJ heat shock protein family (Hsp40) member C1 (DNAJC1), mRNA</td><td>0.893005052</td><td>1.165122413</td><td>1.126346439</td><td>0.992416658</td><td>0.9060385</td></tr>
<tr><td>DNTT</td><td>gi|31343510|ref|NM_177495.2|</td><td>Bos taurus DNA nucleotidylexotransferase (DNTT), mRNA</td><td>n.a.</td><td>n.a.</td><td>n.a.</td><td>n.a.</td><td>n.a.</td></tr>
<tr><td>DNTT</td><td>gi|741975692|ref|XM_005225429.2|</td><td>PREDICTED: Bos taurus DNA nucleotidylexotransferase (DNTT), transcript variant X1, mRNA</td><td>n.a.</td><td>n.a.</td><td>n.a.</td><td>n.a.</td><td>n.a.</td></tr>
<tr><td>DNTT</td><td>gi|741975693|ref|XM_005225430.2|</td><td>PREDICTED: Bos taurus DNA nucleotidylexotransferase (DNTT), transcript variant X2, mRNA</td><td>n.a.</td><td>n.a.</td><td>n.a.</td><td>n.a.</td><td>n.a.</td></tr>
<tr><td>EPHX2</td><td>gi|115495832|ref|NM_001075534.1|</td><td>Bos taurus epoxide hydrolase 2, cytoplasmic (EPHX2), mRNA</td><td>n.a.</td><td>n.a.</td><td>n.a.</td><td>n.a.</td><td>n.a.</td></tr>
<tr><td>EPHX2</td><td>gi|982933980|ref|XM_015472603.1|</td><td>PREDICTED: Bos taurus epoxide hydrolase 2 (EPHX2), transcript variant X1, mRNA</td><td>1.024419557</td><td>1.413858419</td><td>0.411515022</td><td>1.715009939</td><td>0.406260539</td></tr>
<tr><td>EPHX2</td><td>gi|982933982|ref|XM_005210301.3|</td><td>PREDICTED: Bos taurus epoxide hydrolase 2 (EPHX2), transcript variant X2, mRNA</td><td>0.3921622</td><td>2.03361109</td><td>0.630897058</td><td>0.588396134</td><td>0.91432068</td></tr>
<tr><td>ERCC1</td><td>gi|402692069|ref|NM_001076385.2|</td><td>Bos taurus excision repair cross-complementation group 1 (ERCC1), mRNA</td><td>0.90784024</td><td>1.155268573</td><td>1.09905978</td><td>1.073134808</td><td>0.923407977</td></tr>
<tr><td>ERCC1</td><td>gi|982953638|ref|XM_005219335.2|</td><td>PREDICTED: Bos taurus excision repair cross-complementation group 1 (ERCC1), transcript variant X1, mRNA</td><td>1.070034986</td><td>0.935667158</td><td>1.593185854</td><td>1.226714725</td><td>0.389372774</td></tr>
<tr><td>ERCC1</td><td>gi|982953640|ref|XM_010815082.2|</td><td>PREDICTED: Bos taurus excision repair cross-complementation group 1 (ERCC1), transcript variant X2, mRNA</td><td>1.136719668</td><td>1.029257106</td><td>1.02858969</td><td>1.00446962</td><td>1.08248456</td></tr>
<tr><td>ERCC1</td><td>gi|982953642|ref|XM_005219337.2|</td><td>PREDICTED: Bos taurus excision repair cross-complementation group 1 (ERCC1), transcript variant X3, mRNA</td><td>0.623046119</td><td>1.859997286</td><td>0.742357708</td><td>1.045942313</td><td>1.38308368</td></tr>
<tr><td>ERCC1</td><td>gi|982953644|ref|XM_005219336.3|</td><td>PREDICTED: Bos taurus excision repair cross-complementation group 1 (ERCC1), transcript variant X4, mRNA</td><td>0.499256298</td><td>0.83702013</td><td>1.127473356</td><td>1.035209511</td><td>1.16965879</td></tr>
<tr><td>ERCC3</td><td>gi|114050908|ref|NM_001045988.1|</td><td>Bos taurus excision repair cross-complementation group 3 (ERCC3), mRNA</td><td>0.903082082</td><td>1.254484263</td><td>0.952641509</td><td>1.063502674</td><td>0.981708453</td></tr>
<tr><td>ERCC3</td><td>gi|982912832|ref|XM_015474411.1|</td><td>PREDICTED: Bos taurus excision repair cross-complementation group 3 (ERCC3), transcript variant X1, mRNA</td><td>1.925437745</td><td>0.578681886</td><td>1.445603898</td><td>1.108966555</td><td>1.254972529</td></tr>
<tr><td>ERCC5</td><td>gi|402743313|ref|NM_001035276.2|</td><td>Bos taurus excision repair cross-complementation group 5 (ERCC5), mRNA</td><td>0.868571046</td><td>1.147413521</td><td>1.037696878</td><td>1.010099128</td><td>1.0375853</td></tr>
<tr><td>FDX1</td><td>gi|30842789|ref|NM_181011.2|</td><td>Bos taurus ferredoxin 1 (FDX1), mRNA</td><td>1.029500981</td><td>1.369137699</td><td>1.082157346</td><td>0.968388896</td><td>0.822257177</td></tr>
<tr><td>FKBP1A</td><td>gi|78365304|ref|NM_001035456.1|</td><td>Bos taurus FK506 binding protein 1A, 12kDa (FKBP1A), mRNA</td><td>0.876902889</td><td>1.294365334</td><td>1.171902961</td><td>0.904465049</td><td>1.053137854</td></tr>
<tr><td>FKBP2</td><td>gi|164448579|ref|NM_001113256.1|</td><td>Bos taurus FK506 binding protein 2, 13kDa (FKBP2), transcript variant 1, mRNA</td><td>0.872931321</td><td>1.623260855</td><td>0.944448141</td><td>1.11294892</td><td>0.919456715</td></tr>
<tr><td>FKBP2</td><td>gi|82697384|ref|NM_001037481.1|</td><td>Bos taurus FK506 binding protein 2, 13kDa (FKBP2), transcript variant 2, mRNA</td><td>1.036533544</td><td>0.943544709</td><td>1.528353815</td><td>0.478550457</td><td>1.310548265</td></tr>
<tr><td>FKBP2</td><td>gi|982972005|ref|XM_005227147.3|</td><td>PREDICTED: Bos taurus FK506 binding protein 2 (FKBP2), transcript variant X1, mRNA</td><td>1.095853946</td><td>1.019412286</td><td>1.173188993</td><td>0.757502758</td><td>0.93325536</td></tr>
<tr><td>FKBP2</td><td>gi|982972006|ref|XM_005227148.3|</td><td>PREDICTED: Bos taurus FK506 binding protein 2 (FKBP2), transcript variant X2, mRNA</td><td>1.04706259</td><td>1.209324805</td><td>1.137360531</td><td>0.887410855</td><td>1.174393328</td></tr>
<tr><td>FKBP2</td><td>gi|982972007|ref|XM_005227149.3|</td><td>PREDICTED: Bos taurus FK506 binding protein 2 (FKBP2), transcript variant X3, mRNA</td><td>1.599723023</td><td>0.834180538</td><td>1.419293191</td><td>0.438541621</td><td>1.901797548</td></tr>
<tr><td>FMO1</td><td>gi|329664185|ref|NM_001206199.1|</td><td>Bos taurus flavin containing monooxygenase 1 (FMO1), mRNA</td><td>n.a.</td><td>n.a.</td><td>n.a.</td><td>n.a.</td><td>n.a.</td></tr>
<tr><td>FMO1</td><td>gi|741954096|ref|XM_010813214.1|</td><td>PREDICTED: Bos taurus flavin containing monooxygenase 1 (FMO1), transcript variant X4, mRNA</td><td>n.a.</td><td>n.a.</td><td>n.a.</td><td>n.a.</td><td>n.a.</td></tr>
<tr><td>FMO1</td><td>gi|741954098|ref|XM_010813215.1|</td><td>PREDICTED: Bos taurus flavin containing monooxygenase 1 (FMO1), transcript variant X5, mRNA</td><td>n.a.</td><td>n.a.</td><td>n.a.</td><td>n.a.</td><td>n.a.</td></tr>
<tr><td>FMO1</td><td>gi|982947801|ref|XM_015475072.1|</td><td>PREDICTED: Bos taurus flavin containing monooxygenase 1 (FMO1), transcript variant X1, mRNA</td><td>1.072896334</td><td>0.974687932</td><td>1.198641635</td><td>0.600797598</td><td>1.000703293</td></tr>
<tr><td>FMO1</td><td>gi|982947803|ref|XM_010813211.2|</td><td>PREDICTED: Bos taurus flavin containing monooxygenase 1 (FMO1), transcript variant X2, mRNA</td><td>n.a.</td><td>n.a.</td><td>n.a.</td><td>n.a.</td><td>n.a.</td></tr>
<tr><td>FMO1</td><td>gi|982947804|ref|XM_010813212.2|</td><td>PREDICTED: Bos taurus flavin containing monooxygenase 1 (FMO1), transcript variant X3, mRNA</td><td>n.a.</td><td>n.a.</td><td>n.a.</td><td>n.a.</td><td>n.a.</td></tr>
<tr><td>FMO1</td><td>gi|982947805|ref|XM_005216896.3|</td><td>PREDICTED: Bos taurus flavin containing monooxygenase 1 (FMO1), transcript variant X6, mRNA</td><td>0.758015812</td><td>1.033047466</td><td>0.865325084</td><td>1.477272435</td><td>0.855692217</td></tr>
<tr><td>FMO1</td><td>gi|982947806|ref|XM_010813213.2|</td><td>PREDICTED: Bos taurus flavin containing monooxygenase 1 (FMO1), transcript variant X7, mRNA</td><td>n.a.</td><td>n.a.</td><td>n.a.</td><td>n.a.</td><td>n.a.</td></tr>
<tr><td>FMO1</td><td>gi|982947807|ref|XM_015475073.1|</td><td>PREDICTED: Bos taurus flavin containing monooxygenase 1 (FMO1), transcript variant X8, mRNA</td><td>n.a.</td><td>n.a.</td><td>n.a.</td><td>n.a.</td><td>n.a.</td></tr>
<tr><td>FMO1</td><td>gi|982947809|ref|XM_015475074.1|</td><td>PREDICTED: Bos taurus flavin containing monooxygenase 1 (FMO1), transcript variant X9, mRNA</td><td>n.a.</td><td>n.a.</td><td>n.a.</td><td>n.a.</td><td>n.a.</td></tr>
<tr><td>GADD45A</td><td>gi|77735448|ref|NM_001034247.1|</td><td>Bos taurus growth arrest and DNA-damage-inducible, alpha (GADD45A), mRNA</td><td>1.048970702</td><td>1.490980612</td><td>0.94416656</td><td>1.048439369</td><td>0.890355445</td></tr>
<tr><td>GTF2H1</td><td>gi|114052005|ref|NM_001046150.1|</td><td>Bos taurus general transcription factor IIH, polypeptide 1, 62kDa (GTF2H1), mRNA</td><td>0.949252416</td><td>1.143807523</td><td>0.997050036</td><td>1.075893588</td><td>0.922555731</td></tr>
<tr><td>HIVEP1</td><td>gi|982964805|ref|XM_010818608.2|</td><td>PREDICTED: Bos taurus human immunodeficiency virus type I enhancer binding protein 1 (HIVEP1), mRNA</td><td>0.762546793</td><td>1.098617906</td><td>0.945126376</td><td>1.133187861</td><td>1.029490425</td></tr>
<tr><td>HMOX1</td><td>gi|62460519|ref|NM_001014912.1|</td><td>Bos taurus heme oxygenase (decycling) 1 (HMOX1), mRNA</td><td>0.723575142</td><td>1.296855414</td><td>1.069529045</td><td>0.838147455</td><td>1.100550816</td></tr>
<tr><td>HSP90B1</td><td>gi|31340703|ref|NM_174700.2|</td><td>Bos taurus heat shock protein 90kDa beta (Grp94), member 1 (HSP90B1), mRNA</td><td>0.974219987</td><td>1.134115413</td><td>1.155180821</td><td>1.037795947</td><td>1.068687047</td></tr>
<tr><td>HSPA4L</td><td>gi|982949857|ref|XM_015475394.1|</td><td>PREDICTED: Bos taurus heat shock protein family A (Hsp70) member 4 like (HSPA4L), mRNA</td><td>0.776978292</td><td>0.882656614</td><td>1.36423037</td><td>1.219763921</td><td>0.790914816</td></tr>
<tr><td>HYOU1</td><td>gi|332634821|ref|NM_001206910.1|</td><td>Bos taurus hypoxia up-regulated 1 (HYOU1), transcript variant 1, mRNA</td><td>0.955338948</td><td>1.010301652</td><td>1.214385091</td><td>1.01385853</td><td>0.988338149</td></tr>
<tr><td>HYOU1</td><td>gi|332634825|ref|NM_001206911.1|</td><td>Bos taurus hypoxia up-regulated 1 (HYOU1), transcript variant 2, mRNA</td><td>1.062148884</td><td>1.13847415</td><td>0.878780086</td><td>1.230018923</td><td>1.066141174</td></tr>
<tr><td>HYOU1</td><td>gi|982945695|ref|XM_005215905.3|</td><td>PREDICTED: Bos taurus hypoxia up-regulated 1 (HYOU1), transcript variant X1, mRNA</td><td>1.25937452</td><td>2.057562561</td><td>0.822101875</td><td>1.236737142</td><td>1.213105447</td></tr>
<tr><td>HYOU1</td><td>gi|982945696|ref|XM_005215906.3|</td><td>PREDICTED: Bos taurus hypoxia up-regulated 1 (HYOU1), transcript variant X2, mRNA</td><td>0.566368627</td><td>0.492898226</td><td>1.880454582</td><td>0.706831379</td><td>0.986686865</td></tr>
<tr><td>HYOU1</td><td>gi|982945697|ref|XM_005215907.3|</td><td>PREDICTED: Bos taurus hypoxia up-regulated 1 (HYOU1), transcript variant X3, mRNA</td><td>0.833062246</td><td>0.461908243</td><td>0.880861757</td><td>1.72253702</td><td>0.851877811</td></tr>
<tr><td>HYOU1</td><td>gi|982945698|ref|XM_010812420.2|</td><td>PREDICTED: Bos taurus hypoxia up-regulated 1 (HYOU1), transcript variant X4, mRNA</td><td>1.172769285</td><td>0.797407959</td><td>2.130757504</td><td>0.685963502</td><td>1.328596533</td></tr>
<tr><td>KIF5B</td><td>gi|300797793|ref|NM_001192365.1|</td><td>Bos taurus kinesin family member 5B (KIF5B), mRNA</td><td>1.000217433</td><td>1.122885388</td><td>1.02848397</td><td>1.001380898</td><td>1.01604255</td></tr>
<tr><td>KIF5B</td><td>gi|982942232|ref|XM_005214188.3|</td><td>PREDICTED: Bos taurus kinesin family member 5B (KIF5B), transcript variant X1, mRNA</td><td>0.968282782</td><td>1.093270965</td><td>1.03720533</td><td>1.034064909</td><td>1.064316735</td></tr>
<tr><td>LIG1</td><td>gi|156523207|ref|NM_001102548.1|</td><td>Bos taurus ligase I, DNA, ATP-dependent (LIG1), mRNA</td><td>0.888807364</td><td>1.347963622</td><td>1.117393072</td><td>0.951202999</td><td>0.90308867</td></tr>
<tr><td>LIG1</td><td>gi|982953293|ref|XM_005219131.3|</td><td>PREDICTED: Bos taurus ligase I, DNA, ATP-dependent (LIG1), transcript variant X3, mRNA</td><td>0.993258862</td><td>1.087328226</td><td>1.181624725</td><td>0.909283186</td><td>0.907596555</td></tr>
<tr><td>LIG1</td><td>gi|982953296|ref|XM_010814982.2|</td><td>PREDICTED: Bos taurus ligase I, DNA, ATP-dependent (LIG1), transcript variant X4, mRNA</td><td>0.797065799</td><td>1.15756104</td><td>1.254576452</td><td>1.04846715</td><td>1.044351503</td></tr>
<tr><td>LIG3</td><td>gi|402691813|ref|NM_001038107.2|</td><td>Bos taurus ligase III, DNA, ATP-dependent (LIG3), mRNA</td><td>0.965340281</td><td>1.019183533</td><td>1.200794548</td><td>0.904374506</td><td>1.321958949</td></tr>
<tr><td>LIG3</td><td>gi|741962130|ref|XM_010815767.1|</td><td>PREDICTED: Bos taurus ligase III, DNA, ATP-dependent (LIG3), transcript variant X1, mRNA</td><td>0.614725437</td><td>1.066669392</td><td>1.332895335</td><td>0.955173915</td><td>1.358735524</td></tr>
<tr><td>LIG3</td><td>gi|741962132|ref|XM_005220003.2|</td><td>PREDICTED: Bos taurus ligase III, DNA, ATP-dependent (LIG3), transcript variant X2, mRNA</td><td>0.958729169</td><td>1.108602983</td><td>1.003514517</td><td>1.104582631</td><td>1.031874561</td></tr>
<tr><td>MAP2K3</td><td>gi|139949237|ref|NM_001083693.1|</td><td>Bos taurus mitogen-activated protein kinase kinase 3 (MAP2K3), mRNA</td><td>0.896605525</td><td>1.146872781</td><td>1.016204027</td><td>1.083193068</td><td>1.141907277</td></tr>
<tr><td>MAP2K4</td><td>gi|149642560|ref|NM_001099038.1|</td><td>Bos taurus mitogen-activated protein kinase kinase 4 (MAP2K4), mRNA</td><td>0.651152976</td><td>0.706227737</td><td>1.504723715</td><td>1.148377184</td><td>0.696847935</td></tr>
<tr><td>MAP2K4</td><td>gi|528995214|ref|XM_005220370.1|</td><td>PREDICTED: Bos taurus mitogen-activated protein kinase kinase 4 (MAP2K4), transcript variant X2, mRNA</td><td>0.873307461</td><td>1.521612062</td><td>0.68495937</td><td>1.479303427</td><td>1.125846323</td></tr>
<tr><td>MAP2K4</td><td>gi|982957263|ref|XM_005220369.3|</td><td>PREDICTED: Bos taurus mitogen-activated protein kinase kinase 4 (MAP2K4), transcript variant X1, mRNA</td><td>0.822655354</td><td>1.217455643</td><td>0.95172032</td><td>0.984482166</td><td>0.958976829</td></tr>
<tr><td>MAPK10</td><td>gi|982928780|ref|XM_010806290.2|</td><td>PREDICTED: Bos taurus mitogen-activated protein kinase 10 (MAPK10), transcript variant X2, mRNA</td><td>n.a.</td><td>n.a.</td><td>n.a.</td><td>n.a.</td><td>n.a.</td></tr>
<tr><td>MAPK10</td><td>gi|982928781|ref|XM_005208195.3|</td><td>PREDICTED: Bos taurus mitogen-activated protein kinase 10 (MAPK10), transcript variant X3, mRNA</td><td>n.a.</td><td>n.a.</td><td>n.a.</td><td>n.a.</td><td>n.a.</td></tr>
<tr><td>MAPK10</td><td>gi|982928784|ref|XM_010806292.2|</td><td>PREDICTED: Bos taurus mitogen-activated protein kinase 10 (MAPK10), transcript variant X6, mRNA</td><td>n.a.</td><td>n.a.</td><td>n.a.</td><td>n.a.</td><td>n.a.</td></tr>
<tr><td>MAPK12</td><td>gi|149642960|ref|NM_001098953.1|</td><td>Bos taurus mitogen-activated protein kinase 12 (MAPK12), mRNA</td><td>n.a.</td><td>n.a.</td><td>n.a.</td><td>n.a.</td><td>n.a.</td></tr>
<tr><td>MAPK12</td><td>gi|982927598|ref|XM_005207515.3|</td><td>PREDICTED: Bos taurus mitogen-activated protein kinase 12 (MAPK12), transcript variant X1, mRNA</td><td>n.a.</td><td>n.a.</td><td>n.a.</td><td>n.a.</td><td>n.a.</td></tr>
<tr><td>MAPK12</td><td>gi|982927599|ref|XM_015471458.1|</td><td>PREDICTED: Bos taurus mitogen-activated protein kinase 12 (MAPK12), transcript variant X2, mRNA</td><td>n.a.</td><td>n.a.</td><td>n.a.</td><td>n.a.</td><td>n.a.</td></tr>
<tr><td>MAPK12</td><td>gi|982927601|ref|XM_015471459.1|</td><td>PREDICTED: Bos taurus mitogen-activated protein kinase 12 (MAPK12), transcript variant X3, mRNA</td><td>n.a.</td><td>n.a.</td><td>n.a.</td><td>n.a.</td><td>n.a.</td></tr>
<tr><td>MAPK14</td><td>gi|156120994|ref|NM_001102174.1|</td><td>Bos taurus mitogen-activated protein kinase 14 (MAPK14), mRNA</td><td>0.840618456</td><td>1.29022208</td><td>1.032811686</td><td>0.919864892</td><td>0.8867139</td></tr>
<tr><td>MAPK14</td><td>gi|741970296|ref|XM_010818123.1|</td><td>PREDICTED: Bos taurus mitogen-activated protein kinase 14 (MAPK14), transcript variant X1, mRNA</td><td>n.a.</td><td>n.a.</td><td>n.a.</td><td>n.a.</td><td>n.a.</td></tr>
<tr><td>MAPK14</td><td>gi|741970300|ref|XM_010818125.1|</td><td>PREDICTED: Bos taurus mitogen-activated protein kinase 14 (MAPK14), transcript variant X5, mRNA</td><td>0.726727824</td><td>0.959330785</td><td>1.078242407</td><td>0.762027276</td><td>3.291872032</td></tr>
<tr><td>MAPK14</td><td>gi|982963263|ref|XM_015459772.1|</td><td>PREDICTED: Bos taurus mitogen-activated protein kinase 14 (MAPK14), transcript variant X2, mRNA</td><td>n.a.</td><td>n.a.</td><td>n.a.</td><td>n.a.</td><td>n.a.</td></tr>
<tr><td>MAPK14</td><td>gi|982963266|ref|XM_010818124.2|</td><td>PREDICTED: Bos taurus mitogen-activated protein kinase 14 (MAPK14), transcript variant X3, mRNA</td><td>0.866547676</td><td>1.354357956</td><td>0.869378016</td><td>1.236431065</td><td>0.930497827</td></tr>
<tr><td>MAPK14</td><td>gi|982963268|ref|XM_015459773.1|</td><td>PREDICTED: Bos taurus mitogen-activated protein kinase 14 (MAPK14), transcript variant X4, mRNA</td><td>0.83933054</td><td>1.325940423</td><td>0.926537209</td><td>1.055097615</td><td>0.92238596</td></tr>
<tr><td>MAPKAPK2</td><td>gi|528979330|ref|XM_002693890.3|</td><td>PREDICTED: Bos taurus mitogen-activated protein kinase-activated protein kinase 2 (MAPKAPK2), transcript variant X1, mRNA</td><td>0.899013978</td><td>1.134810631</td><td>0.854874928</td><td>1.151019635</td><td>0.976459462</td></tr>
<tr><td>MAPKAPK2</td><td>gi|982995960|ref|XM_015466677.1|</td><td>PREDICTED: Bos taurus mitogen-activated protein kinase-activated protein kinase 2 (MAPKAPK2), transcript variant X2, mRNA</td><td>1.06623818</td><td>1.107891975</td><td>1.264959574</td><td>0.856277465</td><td>0.756096844</td></tr>
</table>

<table>
<tr><th></th><th></th><th></th><th></th><th></th><th></th><th></th><th></th></tr>
<tr><td>MDM2</td><td>gi|741926373|ref|XM_010805086.1|</td><td>PREDICTED: Bos taurus MDM2 proto-oncogene, E3 ubiquitin protein ligase (MDM2), transcript variant X3, mRNA</td><td>0.994711412</td><td>1.111988722</td><td>0.9510194</td><td>1.103895196</td><td>0.962389421</td></tr>
<tr><td>MPG</td><td>gi|982966004|ref|XM_010800800.2|</td><td>PREDICTED: Bos taurus N-methylpurine DNA glycosylase (MPG), mRNA</td><td>1.035656171</td><td>1.183916381</td><td>1.212524113</td><td>0.822876536</td><td>0.906911187</td></tr>
<tr><td>MSH2</td><td>gi|77736114|ref|NM_001034584.1|</td><td>Bos taurus mutS homolog 2 (MSH2), mRNA</td><td>0.937165829</td><td>1.154104887</td><td>1.057713177</td><td>1.000061037</td><td>0.868543196</td></tr>
<tr><td>MSH3</td><td>gi|156121254|ref|NM_001102305.1|</td><td>Bos taurus mutS homolog 3 (MSH3), mRNA</td><td>0.94471325</td><td>1.105616346</td><td>0.98908078</td><td>1.061060736</td><td>0.895480106</td></tr>
<tr><td>MSH6</td><td>gi|300794941|ref|NM_001192737.1|</td><td>Bos taurus mutS homolog 6 (MSH6), mRNA</td><td>0.57322294</td><td>1.439894285</td><td>1.380142271</td><td>1.030779817</td><td>0.917491495</td></tr>
<tr><td>MYO1A</td><td>gi|402745680|ref|NM_174395.3|</td><td>Bos taurus myosin IA (MYO1A), mRNA</td><td>1.24341986</td><td>2.073783016</td><td>0.348078712</td><td>1.361289113</td><td>0.844586021</td></tr>
<tr><td>MYO1A</td><td>gi|982925555|ref|XM_015471065.1|</td><td>PREDICTED: Bos taurus myosin IA (MYO1A), transcript variant X1, mRNA</td><td>n.a.</td><td>n.a.</td><td>n.a.</td><td>n.a.</td><td>n.a.</td></tr>
<tr><td>MYO1A</td><td>gi|982925558|ref|XM_005206510.3|</td><td>PREDICTED: Bos taurus myosin IA (MYO1A), transcript variant X2, mRNA</td><td>n.a.</td><td>n.a.</td><td>n.a.</td><td>n.a.</td><td>n.a.</td></tr>
<tr><td>MYO1A</td><td>gi|982925559|ref|XM_015471066.1|</td><td>PREDICTED: Bos taurus myosin IA (MYO1A), transcript variant X3, mRNA</td><td>1.698521469</td><td>2.199563227</td><td>0.359191914</td><td>1.231714118</td><td>0.884197596</td></tr>
<tr><td>MYO1A</td><td>gi|982925562|ref|XM_015471067.1|</td><td>PREDICTED: Bos taurus myosin IA (MYO1A), transcript variant X4, mRNA</td><td>n.a.</td><td>n.a.</td><td>n.a.</td><td>n.a.</td><td>n.a.</td></tr>
<tr><td>NEDD8</td><td>gi|75832073|ref|NM_174764.3|</td><td>Bos taurus neural precursor cell expressed, developmentally down-regulated 8 (NEDD8), mRNA</td><td>0.941996123</td><td>1.148648782</td><td>1.081308036</td><td>1.018561258</td><td>0.971635334</td></tr>
<tr><td>NPM1</td><td>gi|982959144|ref|XM_005221418.3|</td><td>PREDICTED: Bos taurus nucleophosmin (nucleolar phosphoprotein B23, numatrin) (NPM1), transcript variant X1, mRNA</td><td>0.943187828</td><td>1.239151157</td><td>1.163760786</td><td>0.991481674</td><td>0.868870856</td></tr>
<tr><td>NQO1</td><td>gi|77736016|ref|NM_001034535.1|</td><td>Bos taurus NAD(P)H dehydrogenase, quinone 1 (NQO1), mRNA</td><td>1.373471705</td><td>1.054210867</td><td>0.957674271</td><td>0.999391191</td><td>1.375543535</td></tr>
<tr><td>NUDT1</td><td>gi|741975375|ref|XM_002698230.4|</td><td>PREDICTED: Bos taurus nudix hydrolase 1 (NUDT1), transcript variant X4, mRNA</td><td>0.927423887</td><td>1.217718039</td><td>1.148819717</td><td>0.569635984</td><td>1.073892473</td></tr>
<tr><td>NUDT1</td><td>gi|982967661|ref|XM_015460537.1|</td><td>PREDICTED: Bos taurus nudix hydrolase 1 (NUDT1), transcript variant X1, mRNA</td><td>0.754532358</td><td>0.683175591</td><td>1.383637431</td><td>1.064351468</td><td>1.203655087</td></tr>
<tr><td>NUDT1</td><td>gi|982967663|ref|XM_015460538.1|</td><td>PREDICTED: Bos taurus nudix hydrolase 1 (NUDT1), transcript variant X2, mRNA</td><td>n.a.</td><td>n.a.</td><td>n.a.</td><td>n.a.</td><td>n.a.</td></tr>
<tr><td>NUDT1</td><td>gi|982967665|ref|XM_002698231.5|</td><td>PREDICTED: Bos taurus nudix hydrolase 1 (NUDT1), transcript variant X3, mRNA</td><td>1.060544739</td><td>1.092396155</td><td>0.979167629</td><td>1.256858754</td><td>0.714085158</td></tr>
<tr><td>ODC1</td><td>gi|32129301|ref|NM_174130.2|</td><td>Bos taurus ornithine decarboxylase 1 (ODC1), mRNA</td><td>1.576610269</td><td>0.563899739</td><td>1.146081406</td><td>1.429584116</td><td>0.822809813</td></tr>
<tr><td>ODC1</td><td>gi|982939264|ref|XM_015473527.1|</td><td>PREDICTED: Bos taurus ornithine decarboxylase 1 (ODC1), transcript variant X1, mRNA</td><td>0.969057429</td><td>1.095646333</td><td>1.071736011</td><td>0.945184556</td><td>0.905667794</td></tr>
<tr><td>ODC1</td><td>gi|982939266|ref|XM_005212918.2|</td><td>PREDICTED: Bos taurus ornithine decarboxylase 1 (ODC1), transcript variant X2, mRNA</td><td>0.906377509</td><td>1.153924078</td><td>1.125413785</td><td>0.878573521</td><td>0.825973407</td></tr>
<tr><td>ODC1</td><td>gi|982939267|ref|XM_010810165.2|</td><td>PREDICTED: Bos taurus ornithine decarboxylase 1 (ODC1), transcript variant X3, mRNA</td><td>0.953260462</td><td>1.010532053</td><td>1.177467506</td><td>0.932460985</td><td>1.061246729</td></tr>
<tr><td>ODC1</td><td>gi|982939268|ref|XM_005212919.3|</td><td>PREDICTED: Bos taurus ornithine decarboxylase 1 (ODC1), transcript variant X4, mRNA</td><td>1.115985034</td><td>1.048576968</td><td>0.953827226</td><td>1.074475425</td><td>0.901959142</td></tr>
<tr><td>OGG1</td><td>gi|161760648|ref|NM_001080285.2|</td><td>Bos taurus 8-oxoguanine DNA glycosylase (OGG1), mRNA</td><td>1.016276491</td><td>1.082045383</td><td>1.053445033</td><td>1.021959824</td><td>0.95296451</td></tr>
<tr><td>PARP1</td><td>gi|31340848|ref|NM_174751.2|</td><td>Bos taurus poly (ADP-ribose) polymerase 1 (PARP1), mRNA</td><td>0.841444081</td><td>1.173815071</td><td>0.962986268</td><td>1.113328501</td><td>0.843475061</td></tr>
<tr><td>PARP1</td><td>gi|982947667|ref|XM_005216796.3|</td><td>PREDICTED: Bos taurus poly(ADP-ribose) polymerase 1 (PARP1), transcript variant X1, mRNA</td><td>0.82609714</td><td>1.206251933</td><td>0.997823079</td><td>1.079833069</td><td>0.893837229</td></tr>
<tr><td>PCNA</td><td>gi|77735938|ref|NM_001034494.1|</td><td>Bos taurus proliferating cell nuclear antigen (PCNA), mRNA</td><td>0.955761286</td><td>1.127808272</td><td>1.157219113</td><td>0.991995595</td><td>0.890437077</td></tr>
<tr><td>PDIA3</td><td>gi|148230373|ref|NM_174333.3|</td><td>Bos taurus protein disulfide isomerase family A, member 3 (PDIA3), mRNA</td><td>0.846261256</td><td>1.187906668</td><td>1.119674102</td><td>1.019620564</td><td>0.972291964</td></tr>
<tr><td>PDIA4</td><td>gi|402744406|ref|NM_001045879.2|</td><td>Bos taurus protein disulfide isomerase family A, member 4 (PDIA4), mRNA</td><td>0.961469647</td><td>1.204070069</td><td>1.168899038</td><td>0.975946605</td><td>1.045909882</td></tr>
<tr><td>PMS2</td><td>gi|329664143|ref|NM_001205938.1|</td><td>Bos taurus PMS2 postmeiotic segregation increased 2 (S. cerevisiae) (PMS2), mRNA</td><td>0.863261125</td><td>1.148062368</td><td>1.013258785</td><td>1.118158155</td><td>0.871333279</td></tr>
<tr><td>POLA1</td><td>gi|329663229|ref|NM_001206065.1|</td><td>Bos taurus polymerase (DNA directed), alpha 1, catalytic subunit (POLA1), mRNA</td><td>0.668477292</td><td>1.500329801</td><td>0.961017155</td><td>0.875747651</td><td>1.322396719</td></tr>
<tr><td>POLA1</td><td>gi|982974544|ref|XM_005228356.3|</td><td>PREDICTED: Bos taurus polymerase (DNA directed), alpha 1, catalytic subunit (POLA1), transcript variant X1, mRNA</td><td>0.913137535</td><td>1.186984887</td><td>0.928153776</td><td>1.077403723</td><td>0.730555199</td></tr>
<tr><td>POLA1</td><td>gi|982974545|ref|XM_015461765.1|</td><td>PREDICTED: Bos taurus polymerase (DNA directed), alpha 1, catalytic subunit (POLA1), transcript variant X2, mRNA</td><td>0.84899228</td><td>0.891932004</td><td>1.393388532</td><td>0.898093573</td><td>1.31698992</td></tr>
<tr><td>POLA1</td><td>gi|982974548|ref|XM_005228359.3|</td><td>PREDICTED: Bos taurus polymerase (DNA directed), alpha 1, catalytic subunit (POLA1), transcript variant X3, mRNA</td><td>0.758614303</td><td>0.676167659</td><td>1.725528707</td><td>0.893270635</td><td>1.556323526</td></tr>
<tr><td>POLA1</td><td>gi|982974549|ref|XM_005228360.3|</td><td>PREDICTED: Bos taurus polymerase (DNA directed), alpha 1, catalytic subunit (POLA1), transcript variant X4, mRNA</td><td>0.997937244</td><td>0.930003591</td><td>0.954610681</td><td>1.347290893</td><td>0.995894049</td></tr>
<tr><td>POLA1</td><td>gi|982974550|ref|XM_015461766.1|</td><td>PREDICTED: Bos taurus polymerase (DNA directed), alpha 1, catalytic subunit (POLA1), transcript variant X5, mRNA</td><td>1.370377387</td><td>1.00600603</td><td>2.428314318</td><td>0.958525426</td><td>0.575931056</td></tr>
<tr><td>POLD1</td><td>gi|31342028|ref|NM_174427.2|</td><td>Bos taurus polymerase (DNA directed), delta 1, catalytic subunit (POLD1), mRNA</td><td>0.991833836</td><td>1.171029272</td><td>1.113469435</td><td>0.909290574</td><td>0.889237511</td></tr>
<tr><td>PON1</td><td>gi|402744865|ref|NM_001046269.2|</td><td>Bos taurus paraoxonase 1 (PON1), mRNA</td><td>n.a.</td><td>n.a.</td><td>n.a.</td><td>n.a.</td><td>n.a.</td></tr>
<tr><td>PON2</td><td>gi|61888861|ref|NM_001013588.1|</td><td>Bos taurus paraoxonase 2 (PON2), mRNA</td><td>0.892192684</td><td>1.15777838</td><td>0.912032203</td><td>1.170340385</td><td>1.141133021</td></tr>
<tr><td>PON3</td><td>gi|115496164|ref|NM_001075479.1|</td><td>Bos taurus paraoxonase 3 (PON3), mRNA</td><td>1.153784891</td><td>1.466611446</td><td>0.452596817</td><td>1.673290767</td><td>1.11804504</td></tr>
<tr><td>PON3</td><td>gi|982920878|ref|XM_010803994.2|</td><td>PREDICTED: Bos taurus paraoxonase 3 (PON3), transcript variant X1, mRNA</td><td>n.a.</td><td>n.a.</td><td>n.a.</td><td>n.a.</td><td>n.a.</td></tr>
<tr><td>PPARA</td><td>gi|77404270|ref|NM_001034036.1|</td><td>Bos taurus peroxisome proliferator-activated receptor alpha (PPARA), mRNA</td><td>0.908813563</td><td>1.111478441</td><td>0.858821751</td><td>1.122811141</td><td>0.895188849</td></tr>
<tr><td>PPARA</td><td>gi|982927529|ref|XM_005207472.3|</td><td>PREDICTED: Bos taurus peroxisome proliferator-activated receptor alpha (PPARA), transcript variant X1, mRNA</td><td>0.842282631</td><td>1.092204836</td><td>0.990435196</td><td>0.968223044</td><td>1.066717022</td></tr>
<tr><td>PPARA</td><td>gi|982927530|ref|XM_015471442.1|</td><td>PREDICTED: Bos taurus peroxisome proliferator-activated receptor alpha (PPARA), transcript variant X2, mRNA</td><td>n.a.</td><td>n.a.</td><td>n.a.</td><td>n.a.</td><td>n.a.</td></tr>
<tr><td>PPARA</td><td>gi|982927532|ref|XM_010805796.2|</td><td>PREDICTED: Bos taurus peroxisome proliferator-activated receptor alpha (PPARA), transcript variant X3, mRNA</td><td>n.a.</td><td>n.a.</td><td>n.a.</td><td>n.a.</td><td>n.a.</td></tr>
<tr><td>PPARD</td><td>gi|139948624|ref|NM_001083636.1|</td><td>Bos taurus peroxisome proliferator-activated receptor delta (PPARD), mRNA</td><td>0.700228815</td><td>1.303122986</td><td>0.70828073</td><td>1.277438165</td><td>0.940623921</td></tr>
<tr><td>PPARG</td><td>gi|31341398|ref|NM_181024.2|</td><td>Bos taurus peroxisome proliferator-activated receptor gamma (PPARG), mRNA</td><td>2.458018265</td><td>2.052643046</td><td>0.516146152</td><td>1.400816532</td><td>1.249495005</td></tr>
<tr><td>PTGS1</td><td>gi|198282106|ref|NM_001105323.1|</td><td>Bos taurus prostaglandin-endoperoxide synthase 1 (prostaglandin G/H synthase and cyclooxygenase) (PTGS1), mRNA</td><td>n.a.</td><td>n.a.</td><td>n.a.</td><td>n.a.</td><td>n.a.</td></tr>
<tr><td>RAD23A</td><td>gi|129277536|ref|NM_001082614.1|</td><td>Bos taurus RAD23 homolog A (S. cerevisiae) (RAD23A), mRNA</td><td>1.006076694</td><td>1.098301386</td><td>1.083049274</td><td>0.85850199</td><td>1.094514856</td></tr>
<tr><td>RAD23A</td><td>gi|528954958|ref|XM_005208675.1|</td><td>PREDICTED: Bos taurus RAD23 homolog A, nucleotide excision repair protein (RAD23A), transcript variant X1, mRNA</td><td>0.977507457</td><td>1.242794424</td><td>0.991577725</td><td>1.006741989</td><td>0.963253133</td></tr>
<tr><td>RAD23B</td><td>gi|114052666|ref|NM_001046310.1|</td><td>Bos taurus RAD23 homolog B (S. cerevisiae) (RAD23B), mRNA</td><td>1.010285683</td><td>1.182813358</td><td>1.056653953</td><td>0.96710471</td><td>0.965992247</td></tr>
<tr><td>RAD50</td><td>gi|332309232|ref|NM_001206868.1|</td><td>Bos taurus RAD50 homolog (S. cerevisiae) (RAD50), mRNA</td><td>0.951306748</td><td>1.232718357</td><td>1.104708556</td><td>0.98298751</td><td>0.872715981</td></tr>
<tr><td>RAD51</td><td>gi|402692337|ref|NM_001046179.2|</td><td>Bos taurus RAD51 recombinase (RAD51), mRNA</td><td>1.032271647</td><td>1.088472587</td><td>1.020674393</td><td>0.981073884</td><td>0.852449911</td></tr>
<tr><td>RAD51</td><td>gi|982936713|ref|XM_010809172.2|</td><td>PREDICTED: Bos taurus RAD51 recombinase (RAD51), transcript variant X1, mRNA</td><td>0.836401837</td><td>1.162132088</td><td>1.050147212</td><td>0.93146424</td><td>0.828807551</td></tr>
<tr><td>RAD51</td><td>gi|982936714|ref|XM_010809173.2|</td><td>PREDICTED: Bos taurus RAD51 recombinase (RAD51), transcript variant X2, mRNA</td><td>1.116268866</td><td>1.116961472</td><td>0.981627854</td><td>1.159748936</td><td>1.044866416</td></tr>
<tr><td>RAD52</td><td>gi|982927104|ref|XM_010805644.2|</td><td>PREDICTED: Bos taurus RAD52 homolog, DNA repair protein (RAD52), transcript variant X7, mRNA</td><td>n.a.</td><td>n.a.</td><td>n.a.</td><td>n.a.</td><td>n.a.</td></tr>
<tr><td>RAD54L</td><td>gi|195539536|ref|NM_001130766.1|</td><td>Bos taurus RAD54-like (S. cerevisiae) (RAD54L), mRNA</td><td>0.716954855</td><td>1.035101942</td><td>1.100802563</td><td>1.212803239</td><td>0.854191853</td></tr>
<tr><td>RAD54L</td><td>gi|195539536|ref|NM_001130766.1|</td><td>Bos taurus RAD54-like (S. cerevisiae) (RAD54L), mRNA</td><td>0.716954855</td><td>1.035101942</td><td>1.100802563</td><td>1.212803239</td><td>0.854191853</td></tr>
<tr><td>RAD54L</td><td>gi|982919865|ref|XM_015465835.1|</td><td>PREDICTED: Bos taurus RAD54-like (S. cerevisiae) (RAD54L), transcript variant X1, mRNA</td><td>1.149675003</td><td>1.097010223</td><td>0.913015656</td><td>1.191275929</td><td>0.944057153</td></tr>
<tr><td>REG3A</td><td>gi|45430002|ref|NM_205787.1|</td><td>Bos taurus regenerating islet-derived 3 alpha (REG3A), mRNA</td><td>n.a.</td><td>n.a.</td><td>n.a.</td><td>n.a.</td><td>n.a.</td></tr>
<tr><td>REG3A</td><td>gi|982938989|ref|XM_005212745.2|</td><td>PREDICTED: Bos taurus regenerating islet-derived 3 alpha (REG3A), transcript variant X1, mRNA</td><td>n.a.</td><td>n.a.</td><td>n.a.</td><td>n.a.</td><td>n.a.</td></tr>
<tr><td>RFC1</td><td>gi|982928293|ref|XM_015471607.1|</td><td>PREDICTED: Bos taurus replication factor C subunit 1 (RFC1), transcript variant X1, mRNA</td><td>0.947618784</td><td>1.206709016</td><td>1.054135125</td><td>1.02249923</td><td>0.862121841</td></tr>
<tr><td>RFC1</td><td>gi|982983179|ref|XM_015463745.1|</td><td>PREDICTED: Bos taurus replication factor C subunit 1 (RFC1), transcript variant X2, mRNA</td><td>0.993040459</td><td>1.679116847</td><td>1.480730003</td><td>1.119564799</td><td>1.377955913</td></tr>
<tr><td>RHOB</td><td>gi|118150945|ref|NM_001077922.1|</td><td>Bos taurus ras homolog family member B (RHOB), mRNA</td><td>0.986781104</td><td>1.214160944</td><td>1.003437926</td><td>1.094330294</td><td>1.038150333</td></tr>
<tr><td>RPA2</td><td>gi|402693431|ref|NM_001045984.2|</td><td>Bos taurus replication protein A2, 32kDa (RPA2), mRNA</td><td>0.932595568</td><td>1.14784439</td><td>1.070941405</td><td>1.005428418</td><td>0.889247489</td></tr>
<tr><td>RPS29</td><td>gi|75832074|ref|NM_174804.3|</td><td>Bos taurus ribosomal protein S29 (RPS29), mRNA</td><td>0.934387153</td><td>1.338010253</td><td>1.009206516</td><td>1.040669658</td><td>0.863906635</td></tr>
<tr><td>SDF4</td><td>gi|78369297|ref|NM_001035375.1|</td><td>Bos taurus stromal cell derived factor 4 (SDF4), mRNA</td><td>0.91356942</td><td>1.290066756</td><td>0.993519792</td><td>1.063664988</td><td>0.976807283</td></tr>
<tr><td>SOD1</td><td>gi|31341527|ref|NM_174615.2|</td><td>Bos taurus superoxide dismutase 1, soluble (SOD1), mRNA</td><td>1.007005464</td><td>1.192744458</td><td>0.969604248</td><td>1.011573217</td><td>0.930967125</td></tr>
<tr><td>SOD2</td><td>gi|741938633|ref|XM_010808730.1|</td><td>PREDICTED: Bos taurus superoxide dismutase 2, mitochondrial (SOD2), transcript variant X1, mRNA</td><td>1.016365962</td><td>1.325003048</td><td>1.023179794</td><td>0.964304227</td><td>0.904935343</td></tr>
<tr><td>SOD2</td><td>gi|88853815|ref|NM_201527.2|</td><td>Bos taurus superoxide dismutase 2, mitochondrial (SOD2), mRNA</td><td>1.069242369</td><td>1.249862228</td><td>0.924860132</td><td>0.928438687</td><td>0.905395026</td></tr>
<tr><td>SOD2</td><td>gi|982988233|ref|XM_015464889.1|</td><td>PREDICTED: Bos taurus superoxide dismutase 2, mitochondrial (SOD2), transcript variant X2, mRNA</td><td>n.a.</td><td>n.a.</td><td>n.a.</td><td>n.a.</td><td>n.a.</td></tr>
<tr><td>SQSTM1</td><td>gi|110626168|ref|NM_176641.1|</td><td>Bos taurus sequestosome 1 (SQSTM1), mRNA</td><td>0.88881091</td><td>1.117330269</td><td>0.915357663</td><td>1.068129074</td><td>1.133124429</td></tr>
<tr><td>SQSTM1</td><td>gi|982929187|ref|XM_010806433.2|</td><td>PREDICTED: Bos taurus sequestosome 1 (SQSTM1), transcript variant X1, mRNA</td><td>0.858792803</td><td>1.130260375</td><td>0.947475132</td><td>1.012744386</td><td>1.093531055</td></tr>
<tr><td>TCP1</td><td>gi|84000142|ref|NM_001038086.1|</td><td>Bos taurus t-complex 1 (TCP1), mRNA</td><td>0.994845024</td><td>1.107880756</td><td>1.152307229</td><td>0.969900668</td><td>1.009080475</td></tr>
<tr><td>TOP1</td><td>gi|330417917|ref|NM_001206487.1|</td><td>Bos taurus topoisomerase (DNA) I (TOP1), mRNA</td><td>0.769949734</td><td>1.195854464</td><td>1.07086956</td><td>0.997232605</td><td>0.980797427</td></tr>
<tr><td>TOP2A</td><td>gi|741963842|ref|XM_010816273.1|</td><td>PREDICTED: Bos taurus topoisomerase (DNA) II alpha (TOP2A), transcript variant X3, mRNA</td><td>1.047727767</td><td>0.973349996</td><td>1.063315782</td><td>1.506887413</td><td>1.034233379</td></tr>
<tr><td>TOP2A</td><td>gi|982957905|ref|XM_005220781.3|</td><td>PREDICTED: Bos taurus topoisomerase (DNA) II alpha (TOP2A), transcript variant X1, mRNA</td><td>1.109173584</td><td>1.197284825</td><td>0.996910386</td><td>0.965800443</td><td>0.891698489</td></tr>
<tr><td>TOP2A</td><td>gi|982957906|ref|XM_015458846.1|</td><td>PREDICTED: Bos taurus topoisomerase (DNA) II alpha (TOP2A), transcript variant X2, mRNA</td><td>1.067748981</td><td>1.059893746</td><td>1.027794514</td><td>1.067185652</td><td>0.867469241</td></tr>
<tr><td>TST</td><td>gi|402692518|ref|NM_177489.3|</td><td>Bos taurus thiosulfate sulfurtransferase (rhodanese) (TST), mRNA</td><td>0.986364185</td><td>0.9130316</td><td>1.193687675</td><td>0.922446281</td><td>1.018196896</td></tr>
<tr><td>TYRP1</td><td>gi|149944623|ref|NM_174480.3|</td><td>Bos taurus tyrosinase-related protein 1 (TYRP1), mRNA</td><td>n.a.</td><td>n.a.</td><td>n.a.</td><td>n.a.</td><td>n.a.</td></tr>
<tr><td>TYRP1</td><td>gi|982933190|ref|XM_015472442.1|</td><td>PREDICTED: Bos taurus tyrosinase-related protein 1 (TYRP1), transcript variant X1, mRNA</td><td>n.a.</td><td>n.a.</td><td>n.a.</td><td>n.a.</td><td>n.a.</td></tr>
<tr><td>TYRP1</td><td>gi|982933192|ref|XM_015472443.1|</td><td>PREDICTED: Bos taurus tyrosinase-related protein 1 (TYRP1), transcript variant X2, mRNA</td><td>n.a.</td><td>n.a.</td><td>n.a.</td><td>n.a.</td><td>n.a.</td></tr>
<tr><td>UBB</td><td>gi|31342776|ref|NM_174133.2|</td><td>Bos taurus ubiquitin B (UBB), mRNA</td><td>0.861145882</td><td>1.270945105</td><td>1.05383897</td><td>0.967213005</td><td>0.930971918</td></tr>
<tr><td>UNG</td><td>gi|402692189|ref|NM_001075513.2|</td><td>Bos taurus uracil-DNA glycosylase (UNG), mRNA</td><td>0.935297125</td><td>1.186350831</td><td>1.059739394</td><td>1.02343935</td><td>0.781797462</td></tr>
<tr><td>UNG</td><td>gi|741956450|ref|XM_005217795.2|</td><td>PREDICTED: Bos taurus uracil DNA glycosylase (UNG), transcript variant X1, mRNA</td><td>1.03404206</td><td>1.245195621</td><td>1.399836885</td><td>0.662258586</td><td>0.77148264</td></tr>
<tr><td>VIM</td><td>gi|110347569|ref|NM_173969.3|</td><td>Bos taurus vimentin (VIM), mRNA</td><td>0.162272337</td><td>0.07494613</td><td>2.088121131</td><td>0.675222653</td><td>0.892752558</td></tr>
<tr><td>XPA</td><td>gi|982933710|ref|XR_001500856.1|</td><td>PREDICTED: Bos taurus xeroderma pigmentosum, complementation group A (XPA), transcript variant X1, misc_RNA</td><td>0.845196017</td><td>1.224055511</td><td>1.022933136</td><td>1.012393798</td><td>0.907976106</td></tr>
<tr><td>XPC</td><td>gi|329663903|ref|NM_001205908.1|</td><td>Bos taurus xeroderma pigmentosum, complementation group C (XPC), mRNA</td><td>0.834719756</td><td>1.040268693</td><td>1.087356068</td><td>1.136110374</td><td>0.966061763</td></tr>
<tr><td>XRCC1</td><td>gi|982953840|ref|XM_005219401.3|</td><td>PREDICTED: Bos taurus X-ray repair complementing defective repair in Chinese hamster cells 1 (XRCC1), transcript variant X1, mRN</td><td>1.089995535</td><td>1.538319214</td><td>1.04197534</td><td>1.042731255</td><td>1.038320967</td></tr>
<tr><td>XRCC1</td><td>gi|982953843|ref|XM_005219402.3|</td><td>PREDICTED: Bos taurus X-ray repair complementing defective repair in Chinese hamster cells 1 (XRCC1), transcript variant X2, mRN</td><td>0.803004853</td><td>1.220880243</td><td>0.977920554</td><td>1.209948451</td><td>0.791921196</td></tr>
<tr><td>XRCC1</td><td>gi|982953845|ref|XM_002695077.4|</td><td>PREDICTED: Bos taurus X-ray repair complementing defective repair in Chinese hamster cells 1 (XRCC1), transcript variant X3, mRN</td><td>1.168081471</td><td>1.025679448</td><td>1.003897952</td><td>1.117663786</td><td>0.704454339</td></tr>
<tr><td>XRCC1</td><td>gi|982953847|ref|XM_005219403.3|</td><td>PREDICTED: Bos taurus X-ray repair complementing defective repair in Chinese hamster cells 1 (XRCC1), transcript variant X4, mRN</td><td>1.069850258</td><td>1.094366144</td><td>1.343902314</td><td>0.704038903</td><td>1.318064988</td></tr>
<tr><td>XRCC1</td><td>gi|982953849|ref|XM_010815130.2|</td><td>PREDICTED: Bos taurus X-ray repair complementing defective repair in Chinese hamster cells 1 (XRCC1), transcript variant X5, mRN</td><td>1.005089835</td><td>1.055458433</td><td>1.038304457</td><td>0.93489902</td><td>1.115826881</td></tr>
<tr><td>XRCC5</td><td>gi|156120928|ref|NM_001102141.1|</td><td>Bos taurus X-ray repair complementing defective repair in Chinese hamster cells 5 (double-strand-break rejoining) (XRCC5), mRNA</td><td>0.898508058</td><td>1.251098459</td><td>1.034708148</td><td>1.058949733</td><td>0.862435223</td></tr>
<tr><td>YWHAZ</td><td>gi|31341044|ref|NM_174814.2|</td><td>Bos taurus tyrosine 3-monooxygenase/tryptophan 5-monooxygenase activation protein, zeta (YWHAZ), mRNA</td><td>0.906099992</td><td>1.213089936</td><td>1.028323478</td><td>1.001459251</td><td>1.11102053</td></tr>
</table>
